# Supplementary material for: Infection and herbicide exposure implicate c-Abl kinase in α-Synuclein Ser129 phosphorylation
Source: Cell Commun Signal. 2025 Sep 23;23:396. doi: 10.1186/s12964-025-02399-2 (PMC12455823; doi:10.1186/s12964-025-02399-2)
Supplement: Supplementary file 1 — Supplementary Material 1: Additional file 1. SH-SY5Y cells were infected with H. pylori wild type at MOI 100. Total protein was extracted at different time points and Western blotting was performed to check phosphorylation of CagA at Y99. β-actin is used as loading control [file 12964_2025_2399_MOESM1_ESM.pdf]

Infection and herbicide exposure implicate c-Abl kinase in α-Synuclein Ser129 phosphorylation

Marzieh Ehsani [1# *], Zeyang Sun [1,2#], Alvaro Quevedo-Olmos [1], Gesa Rösler [1], Mahdi Rasa [3,4], David Holthaus [1,5], Saskia F. Erttmann [1], and Thomas F. Meyer [1,6 *]

Original pictures

For all the gels, pager ruller from Thermofischer cat#26616 was used.

Figure_1A_beta_Actin

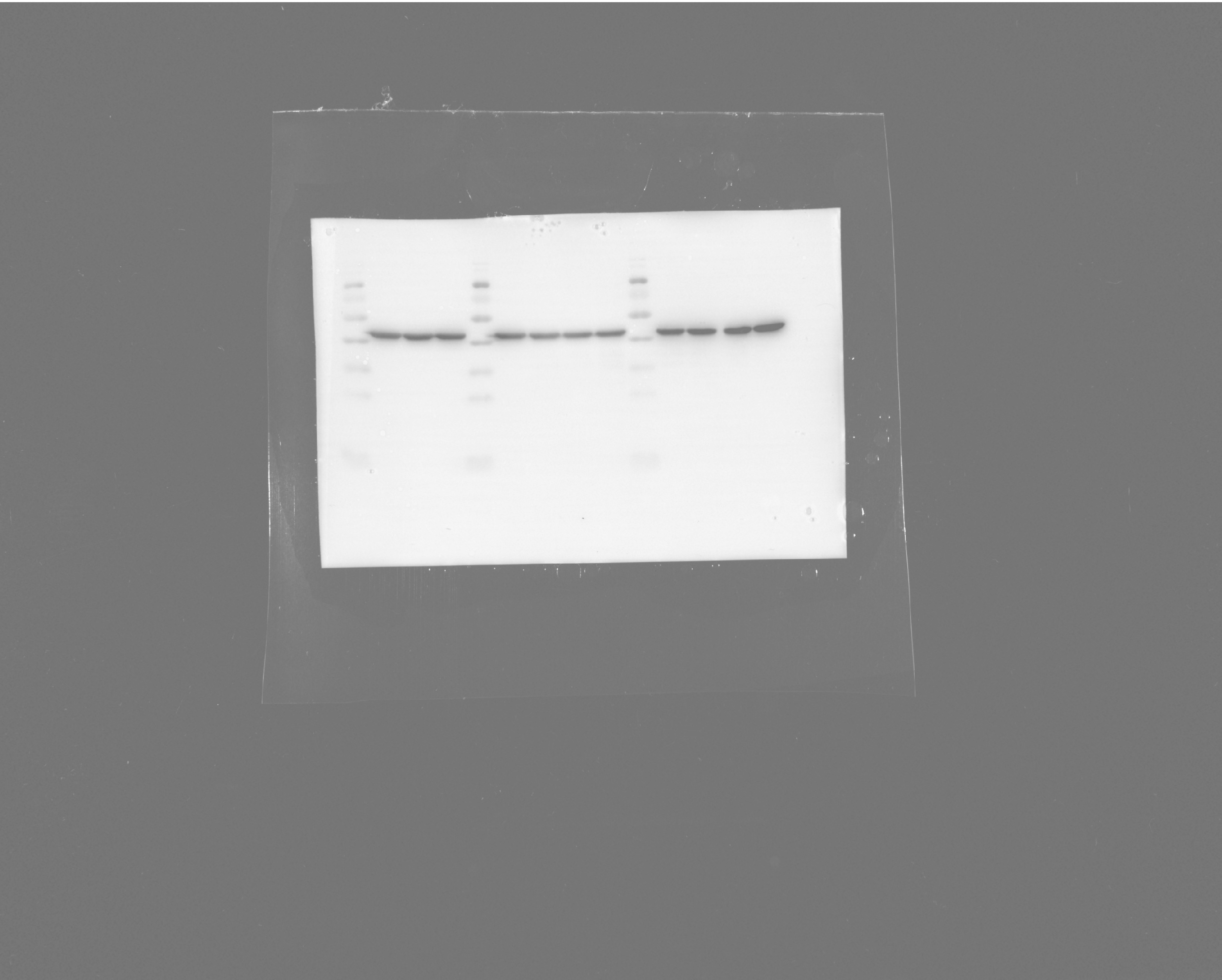

Figure_1A_pSer129 a-synuclein

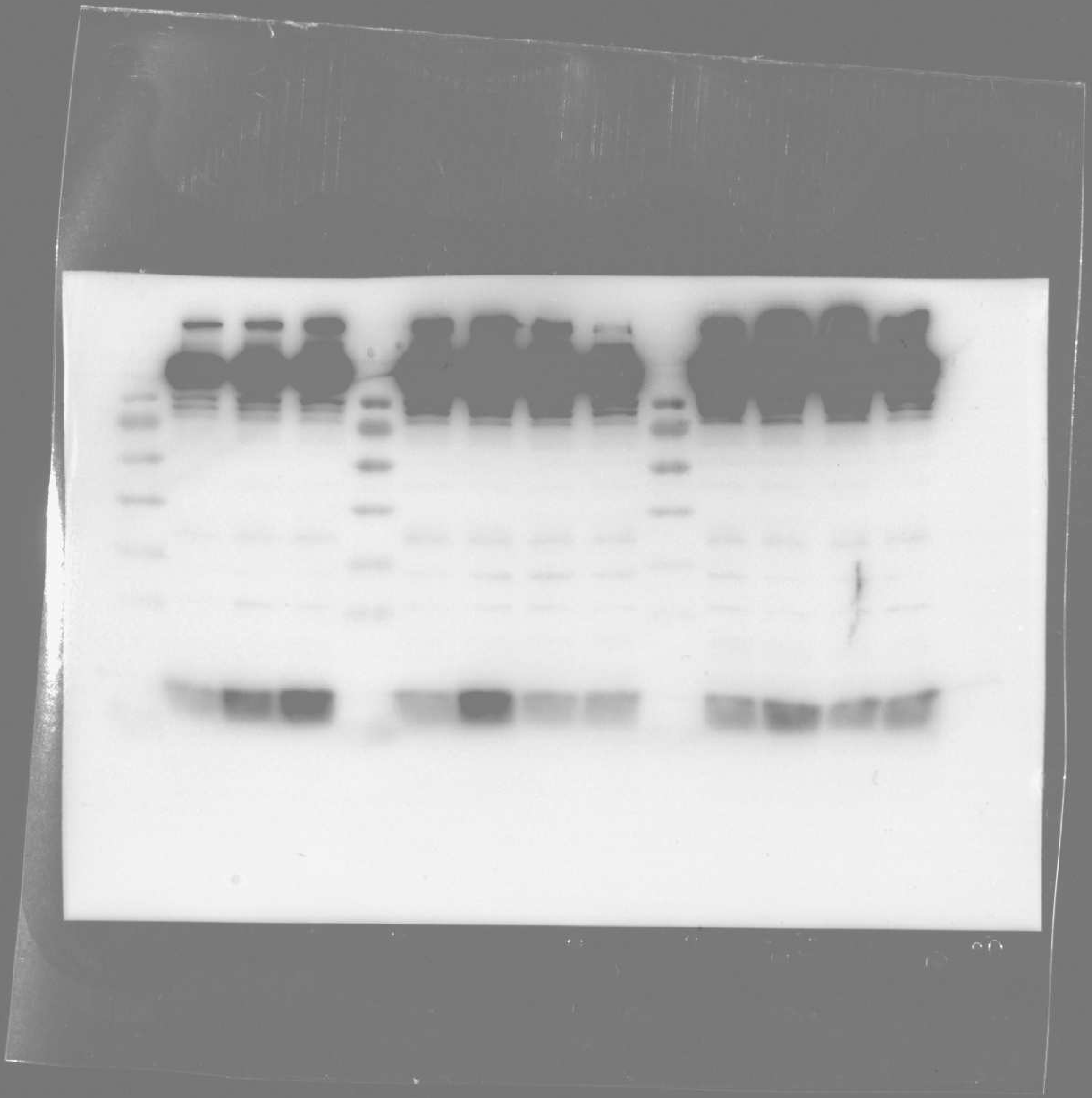

Figure_1A_total a-synuclein

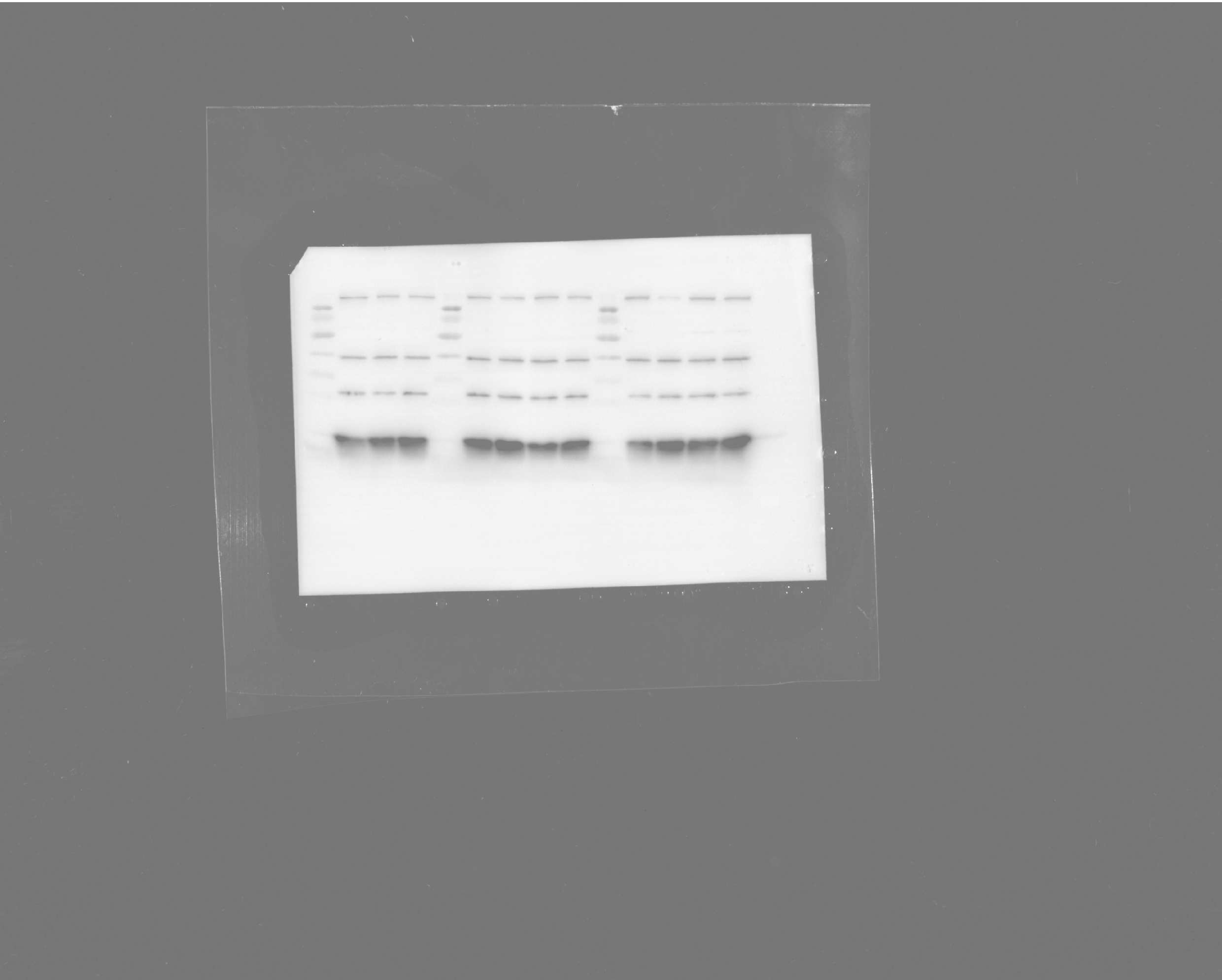

Figure_1C_and_2B_beta_Actin

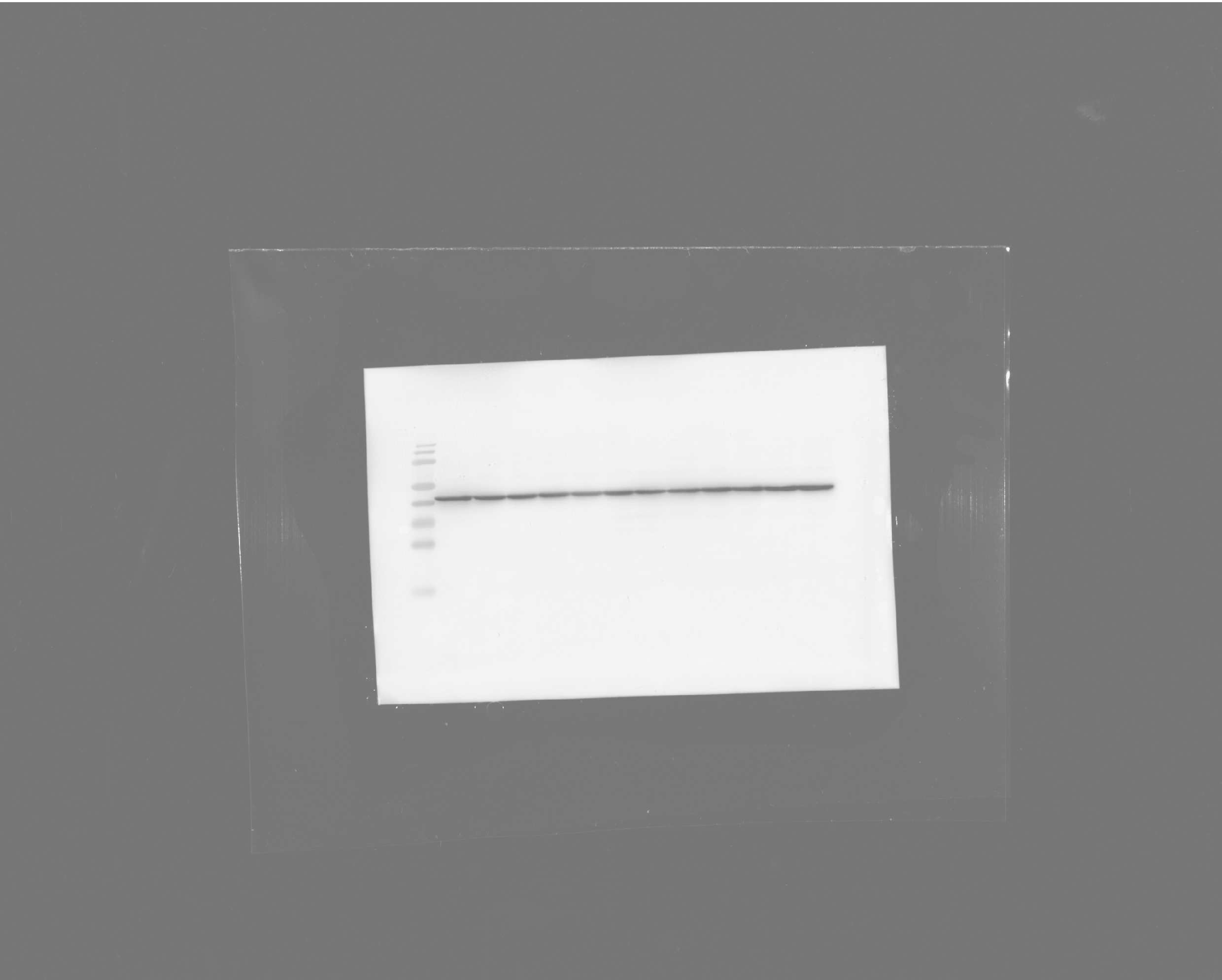

Figure_1C_and_2B_pSer129 a-synuclein

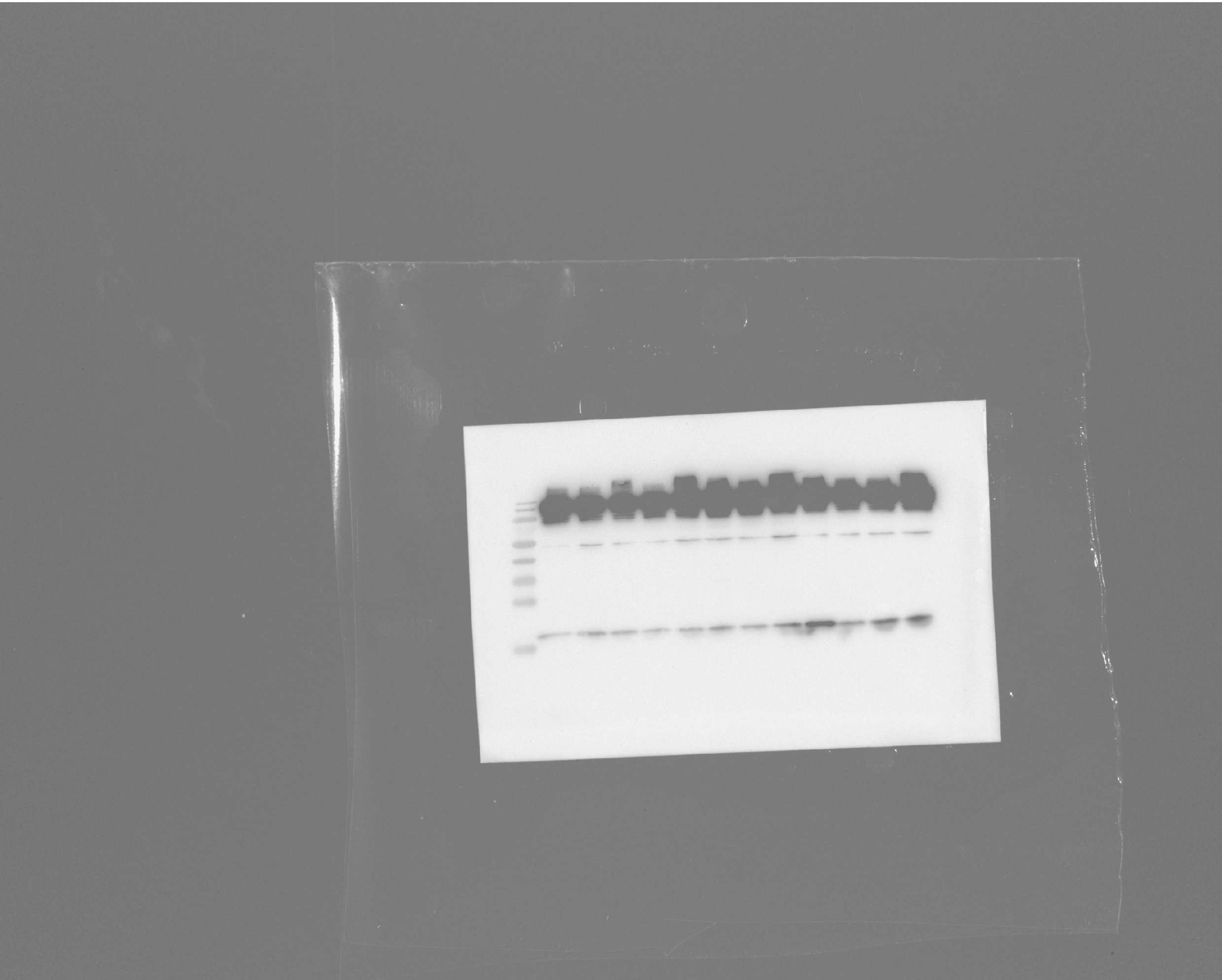

Figure_1C_and_2B_total a-synuclein

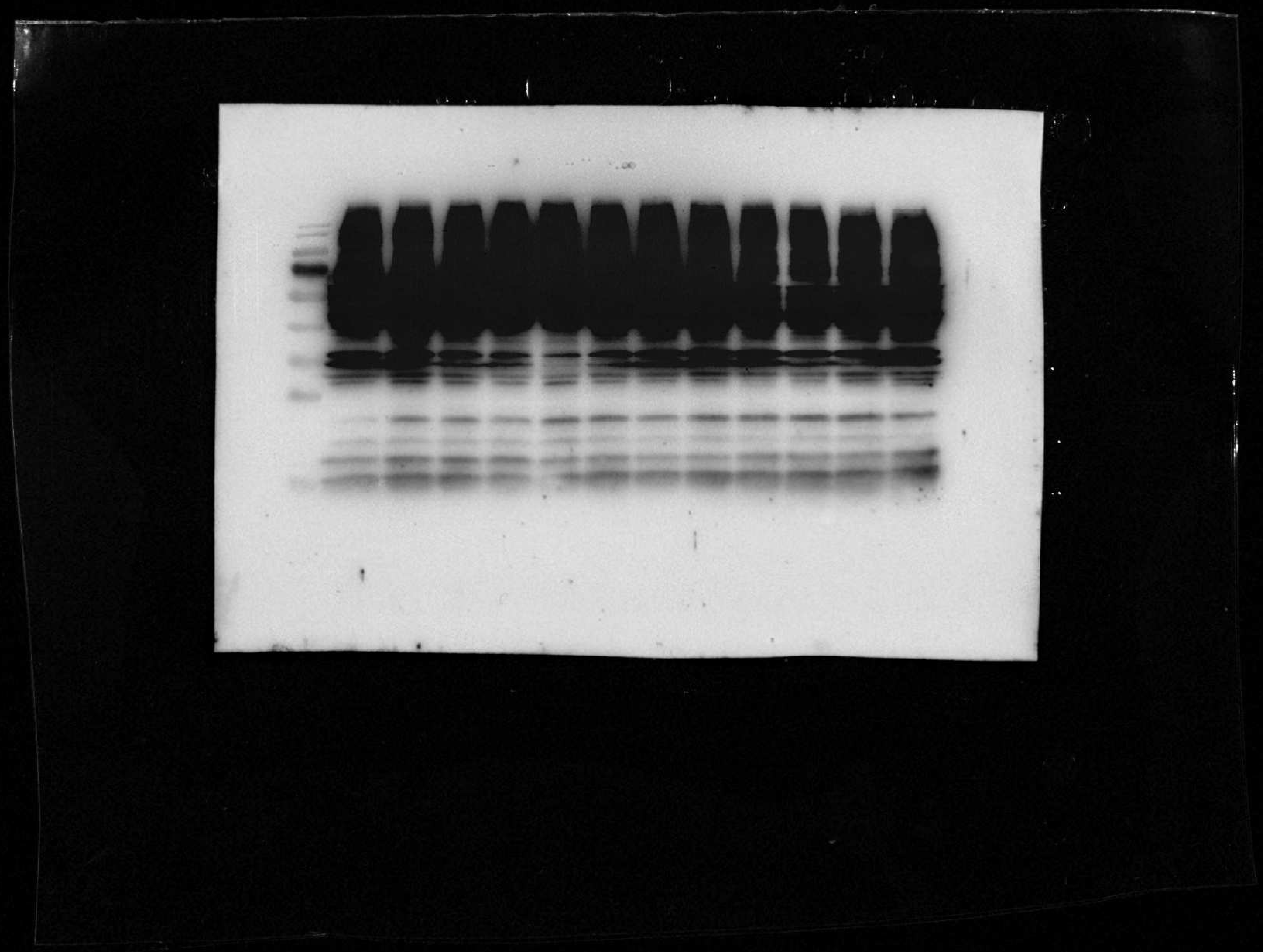

Figure_2B_pY245 c-Abl

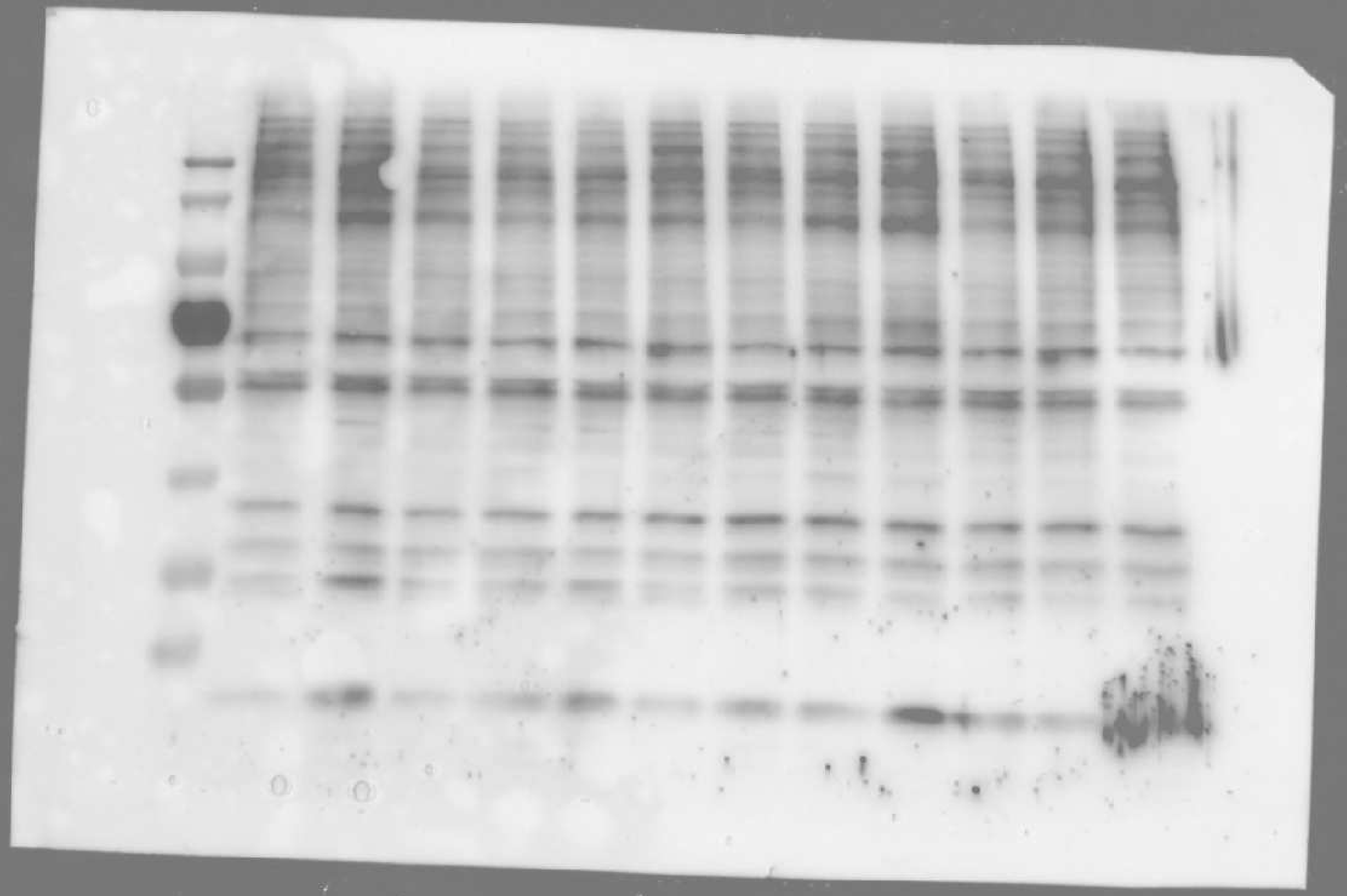

Figure_2B_pY412 cAbl

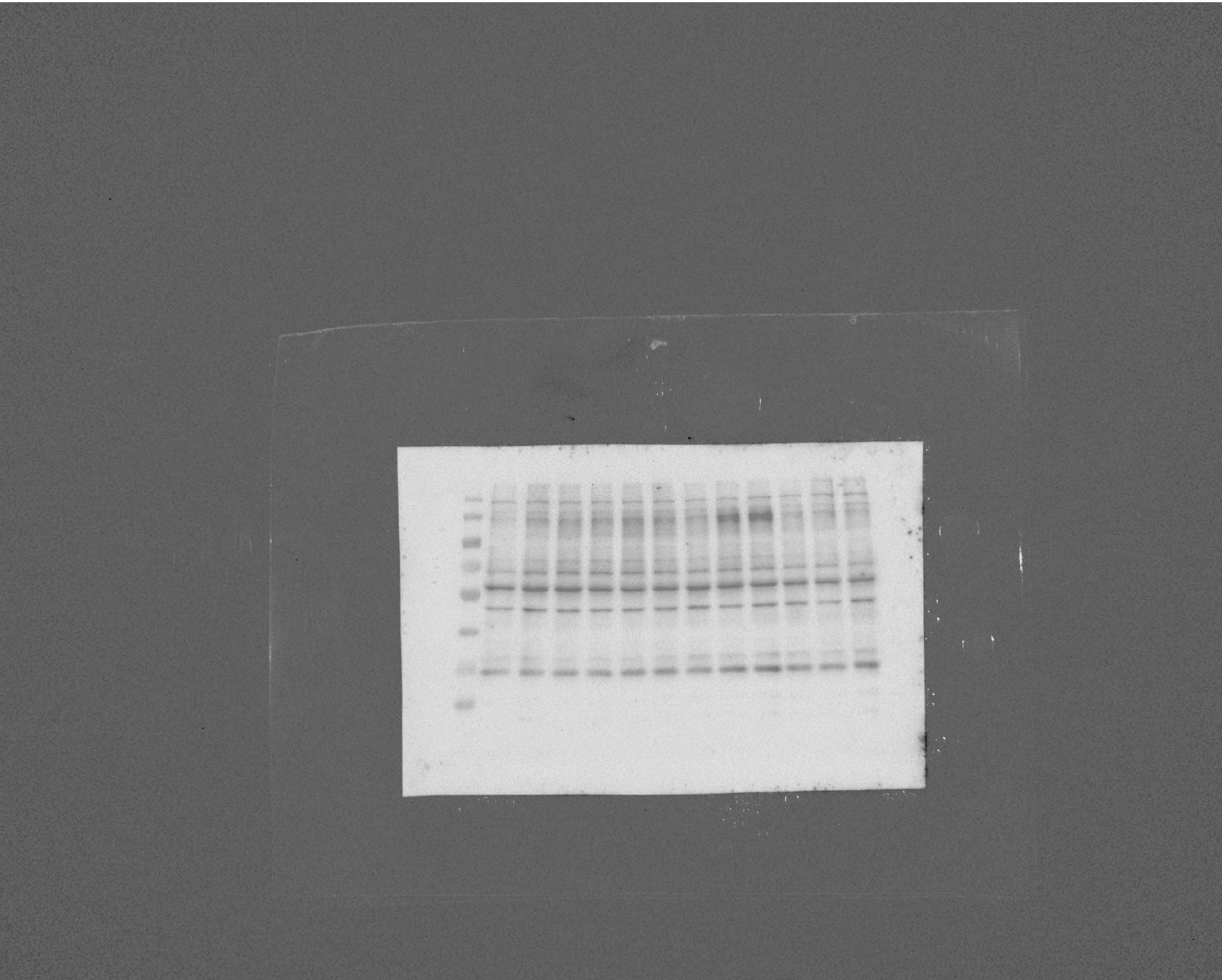

Figure_2B_total cAbl

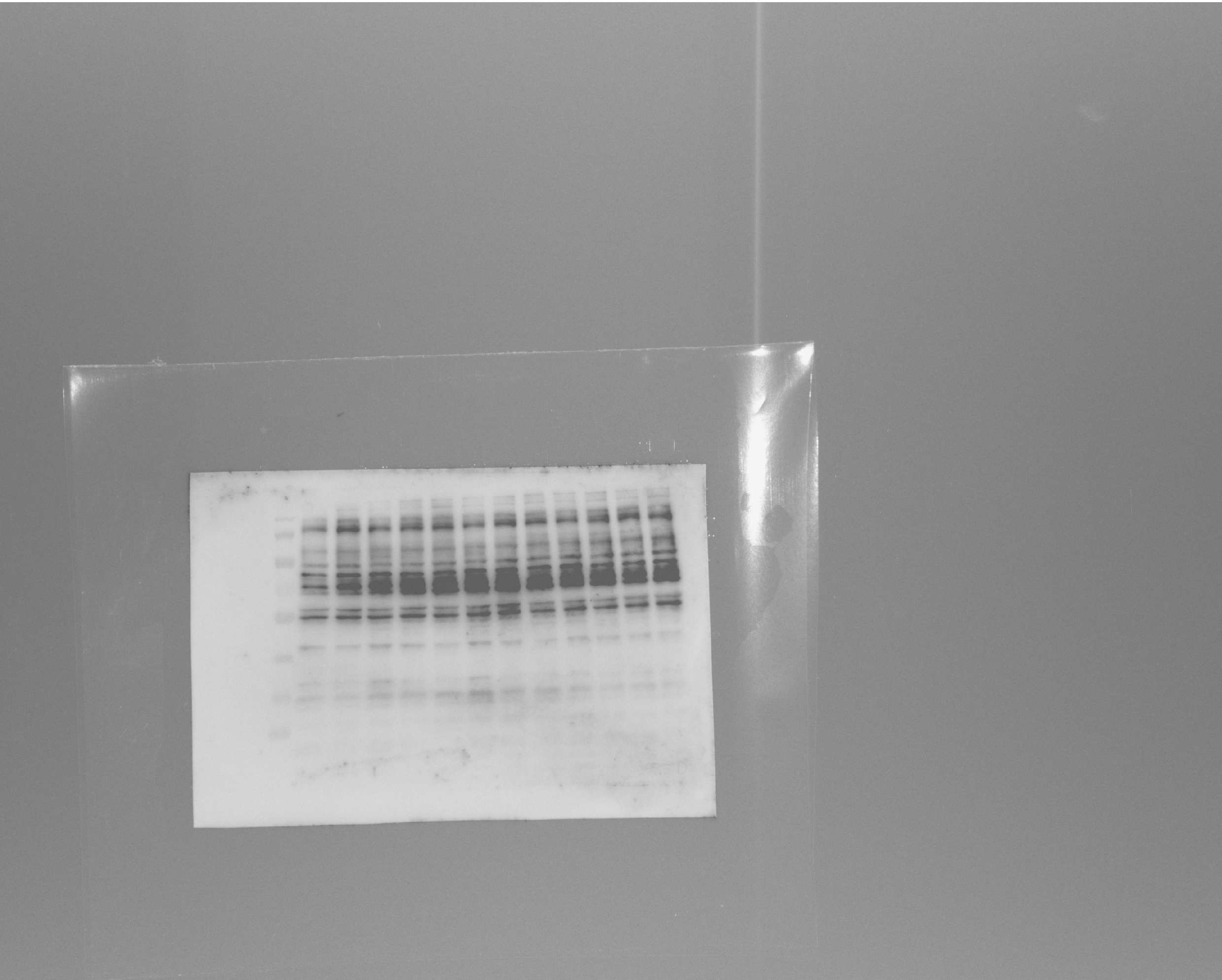

Figure_2C_beta Actin

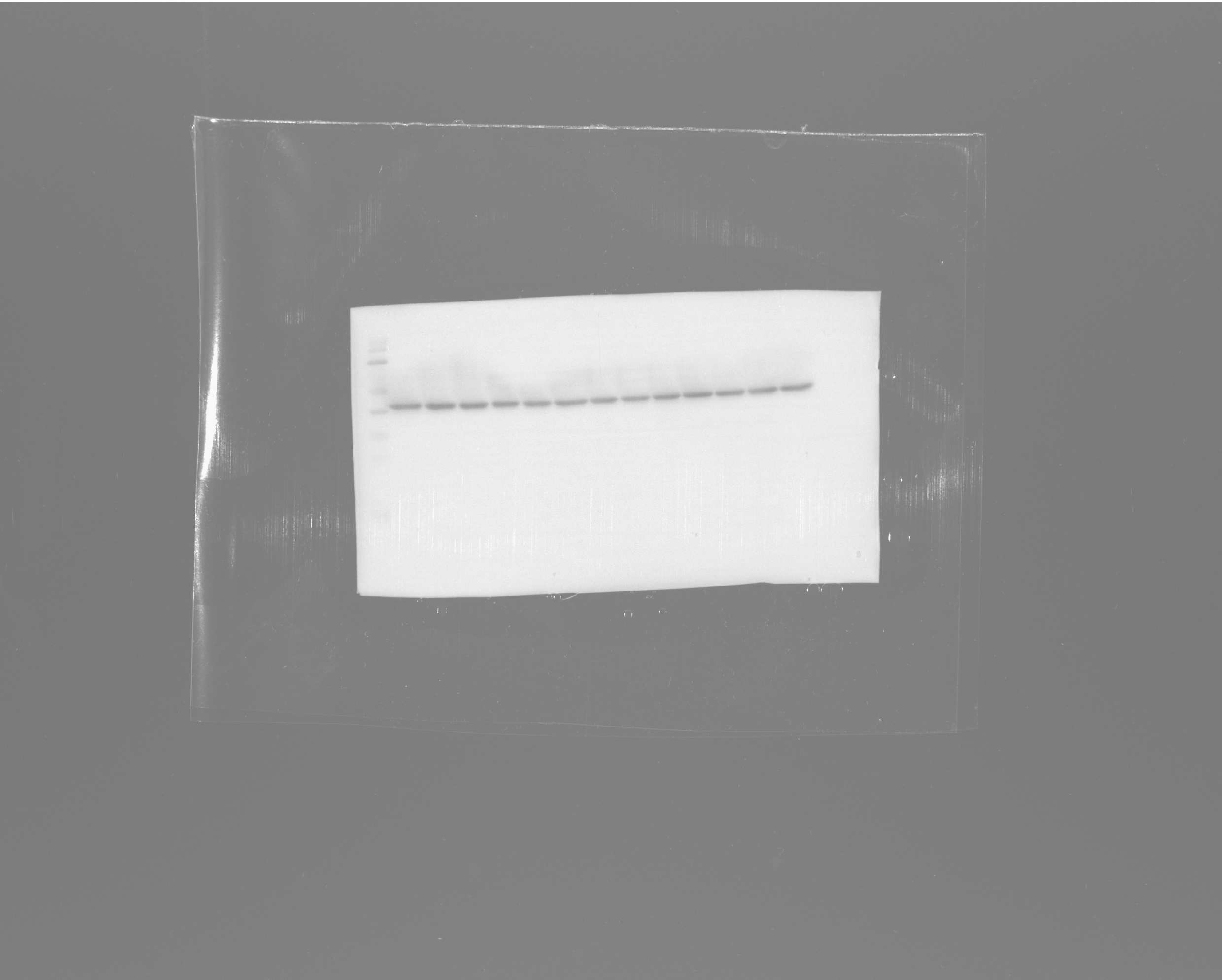

Figure_2C_pS129 a-Synuclein

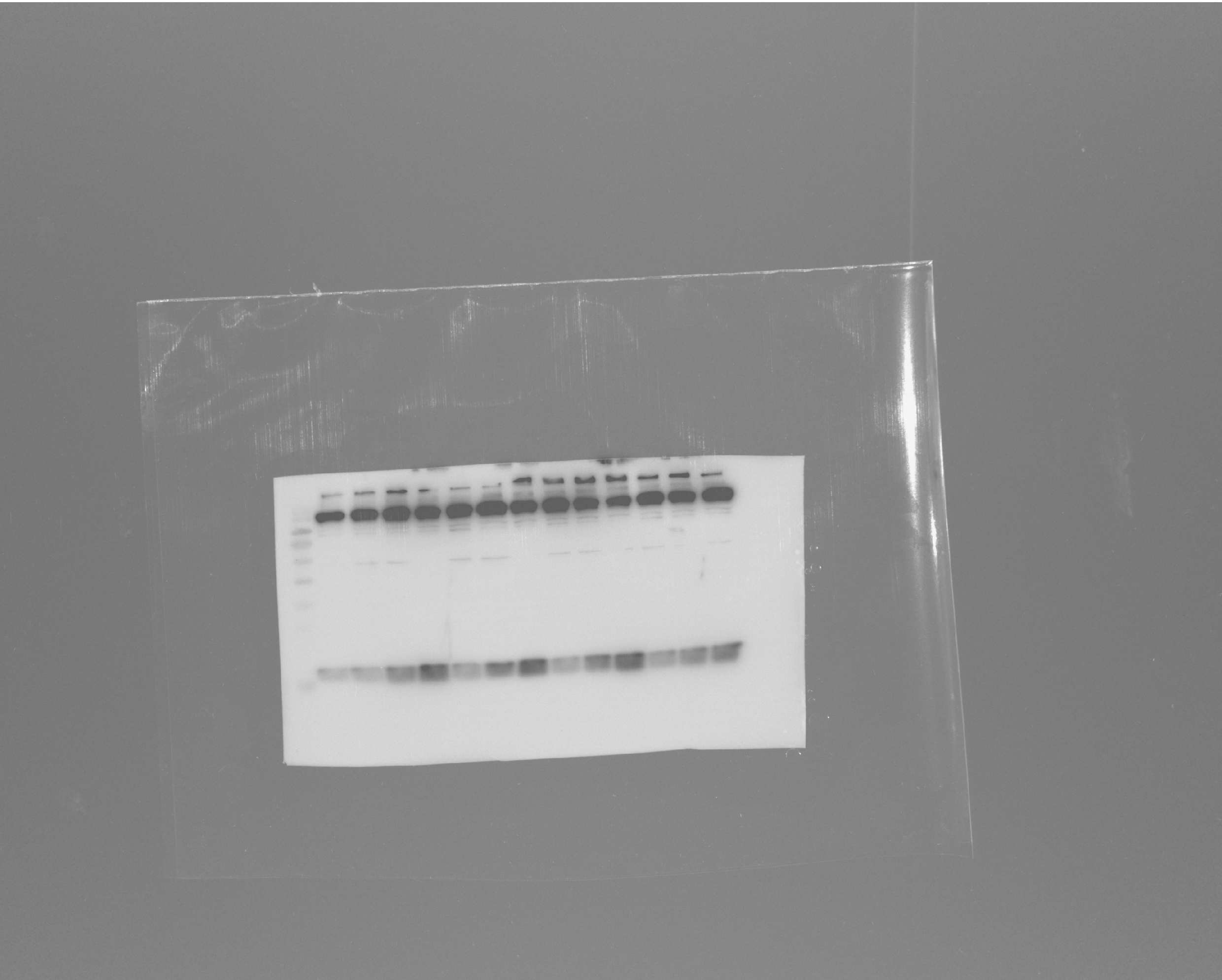

Figure_2C_pY245 cAbl

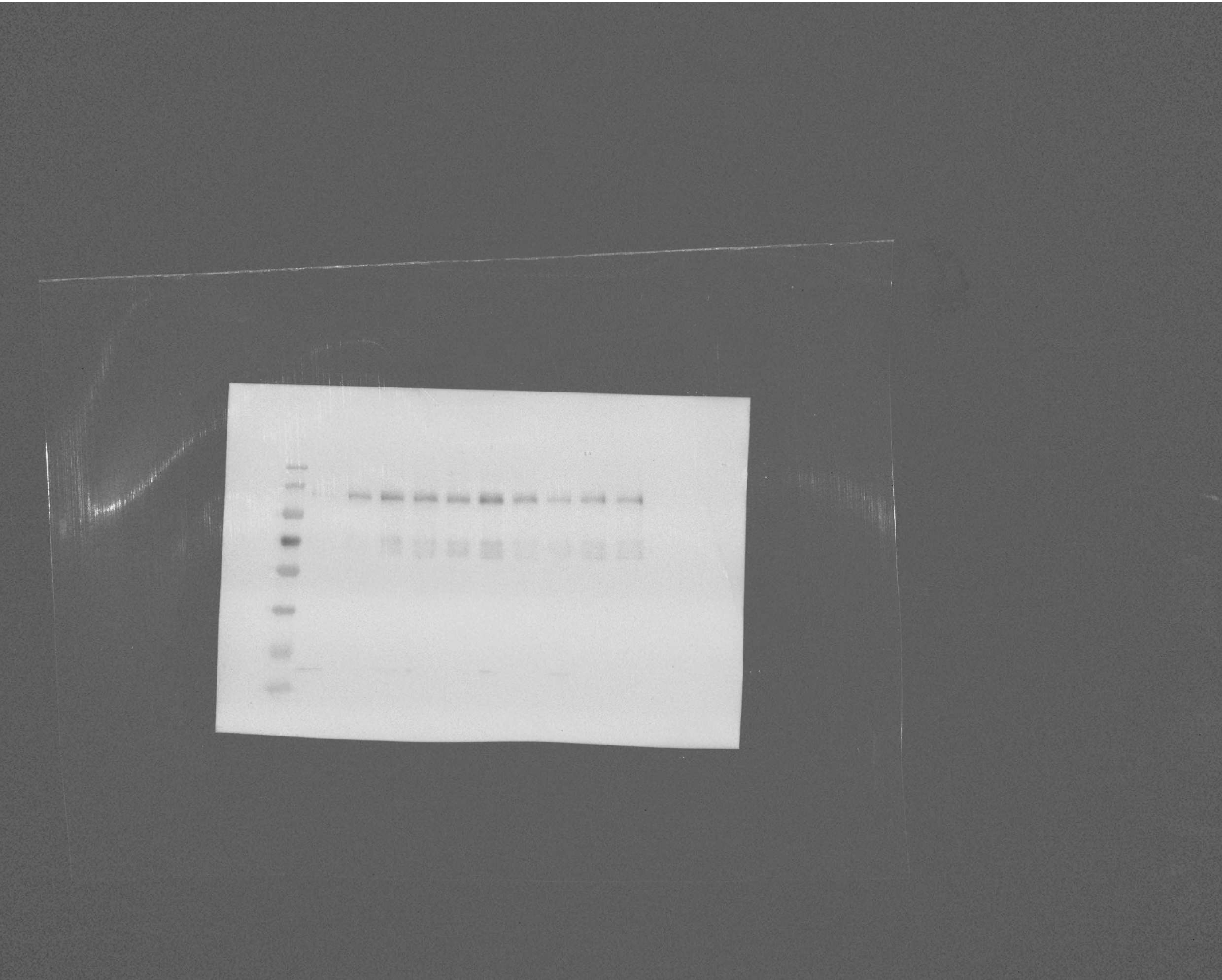

Figure_2C_pY412 cAbl

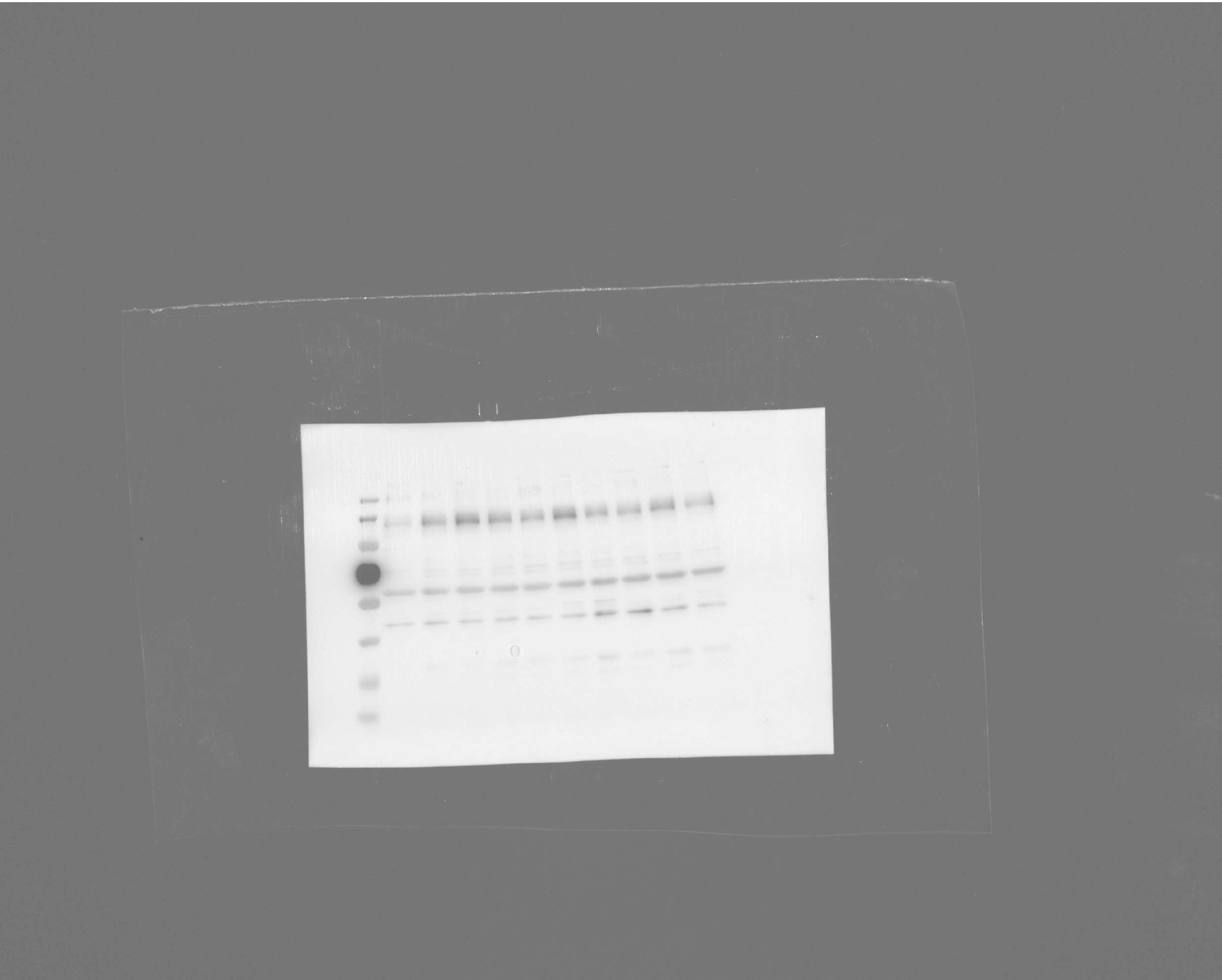

Figure_2C_total a-synuclein

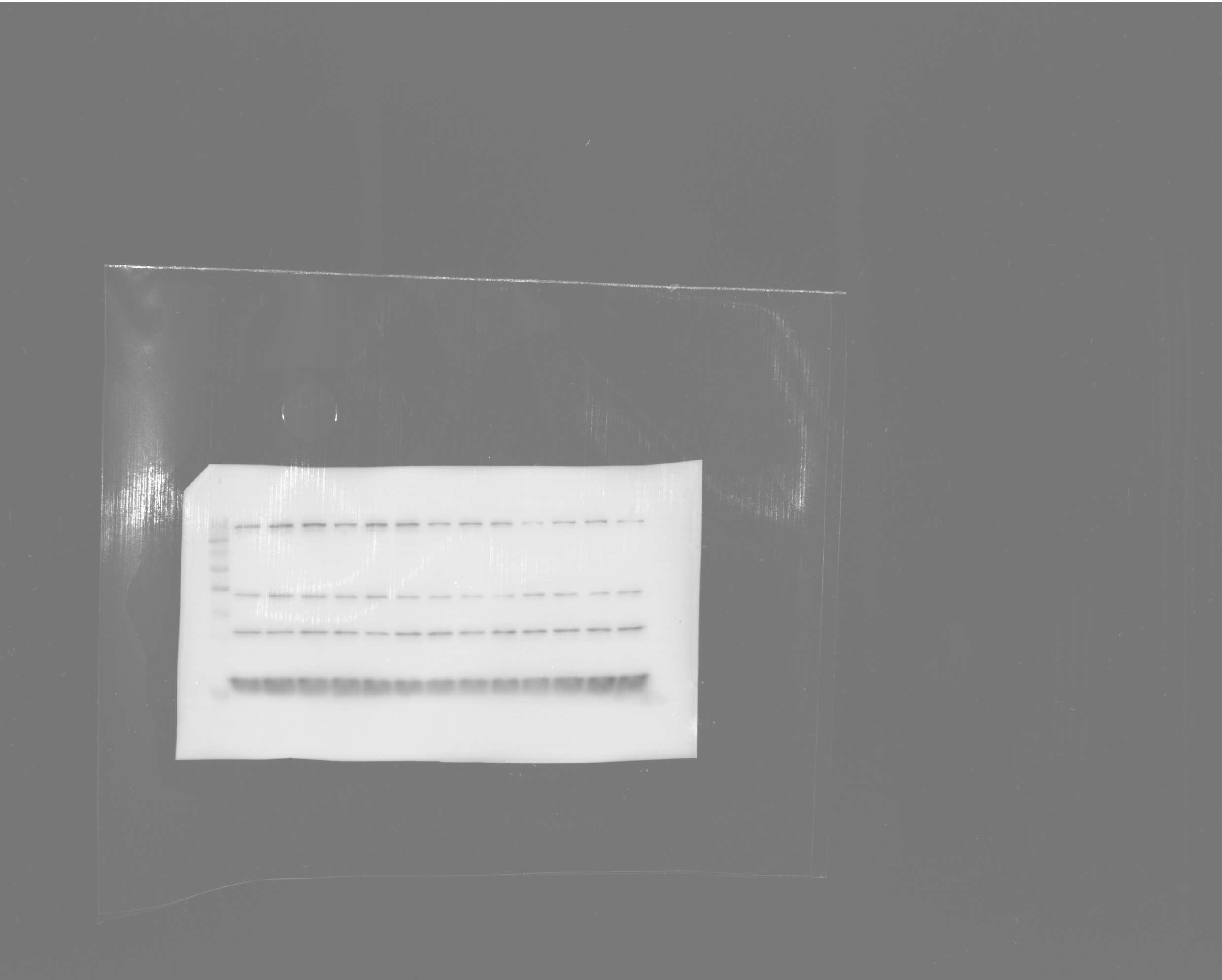

Figure_2C_total cAbl

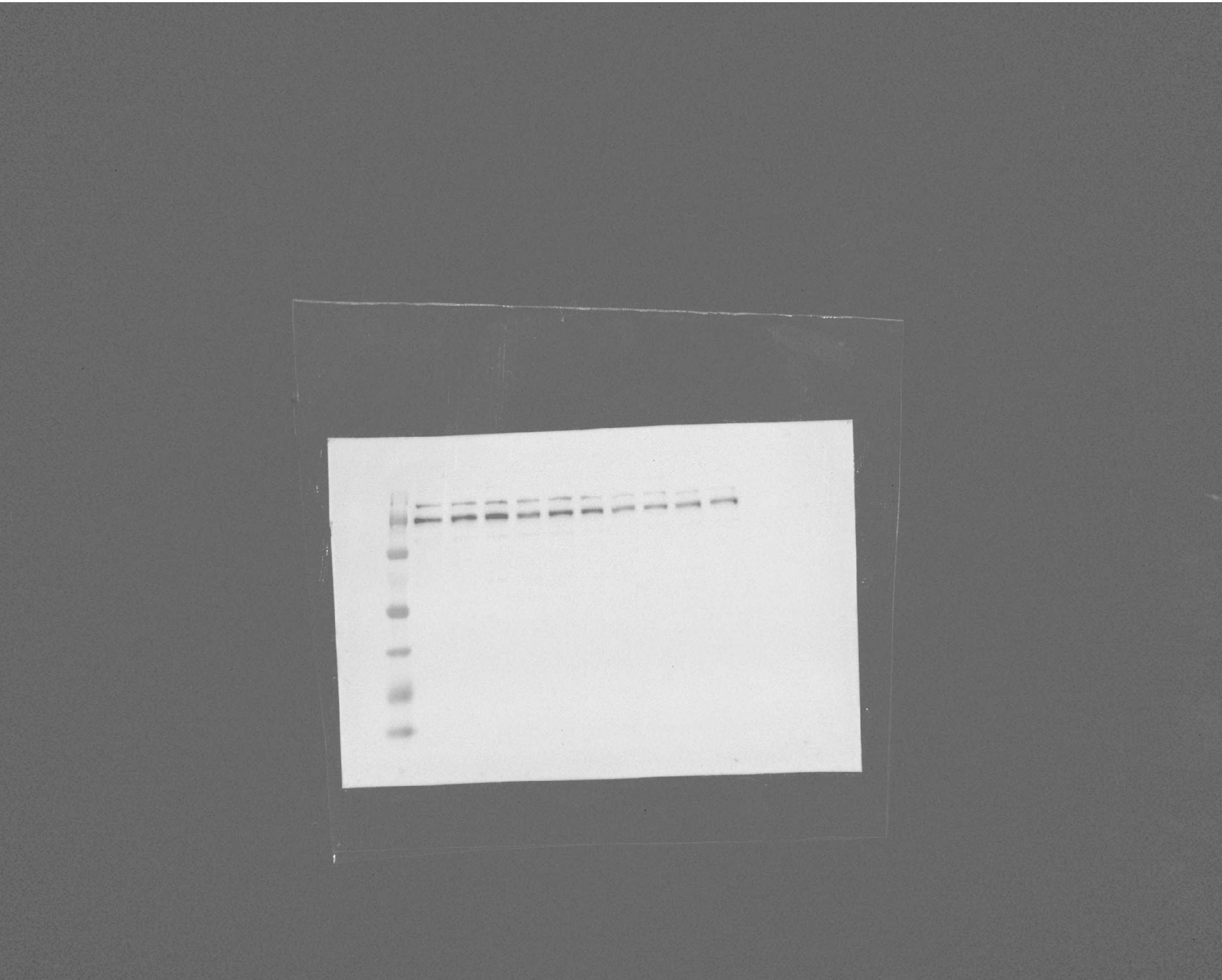

Figure_3A_beta Actin

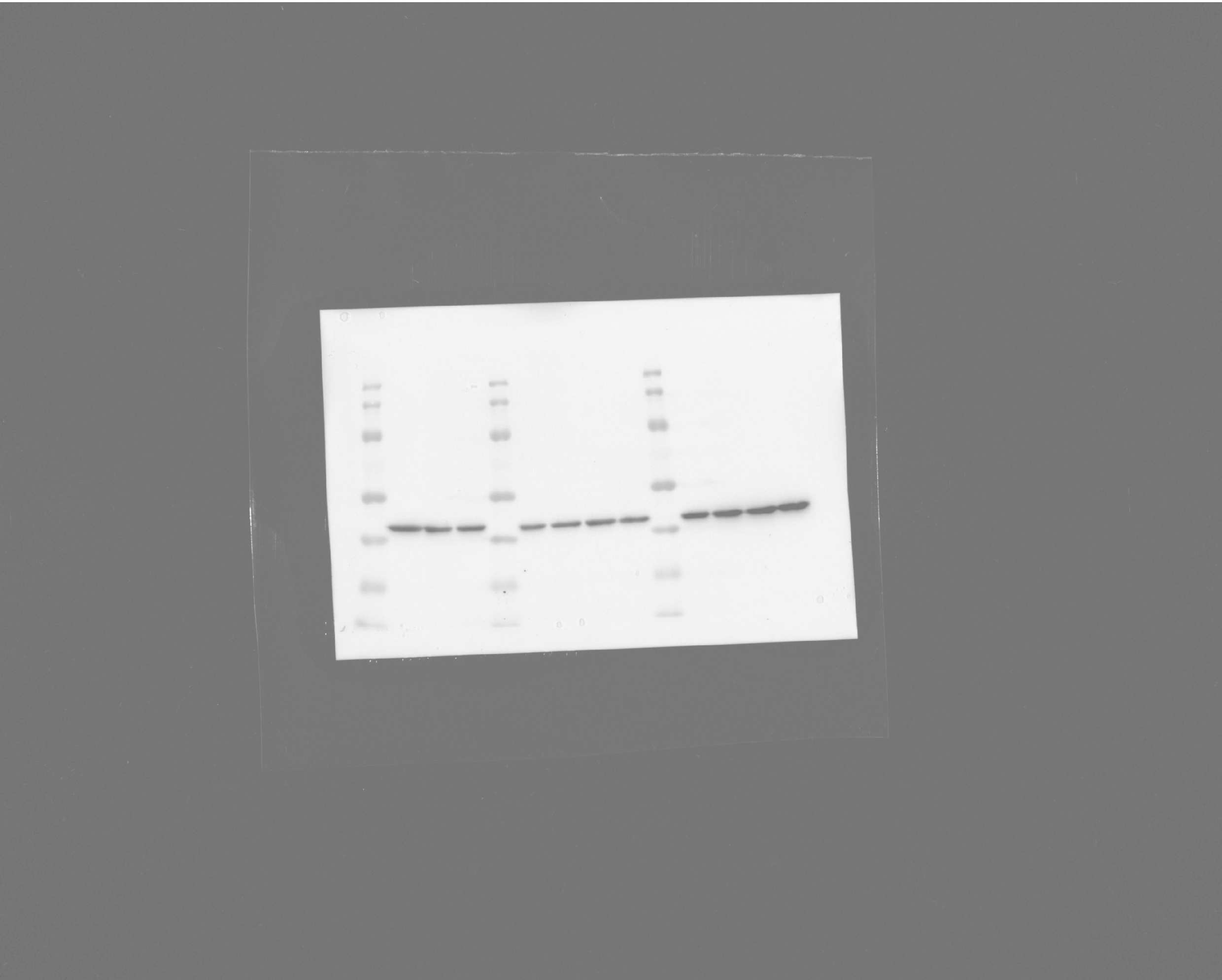

Figure_3A_pS129 a-synuclein

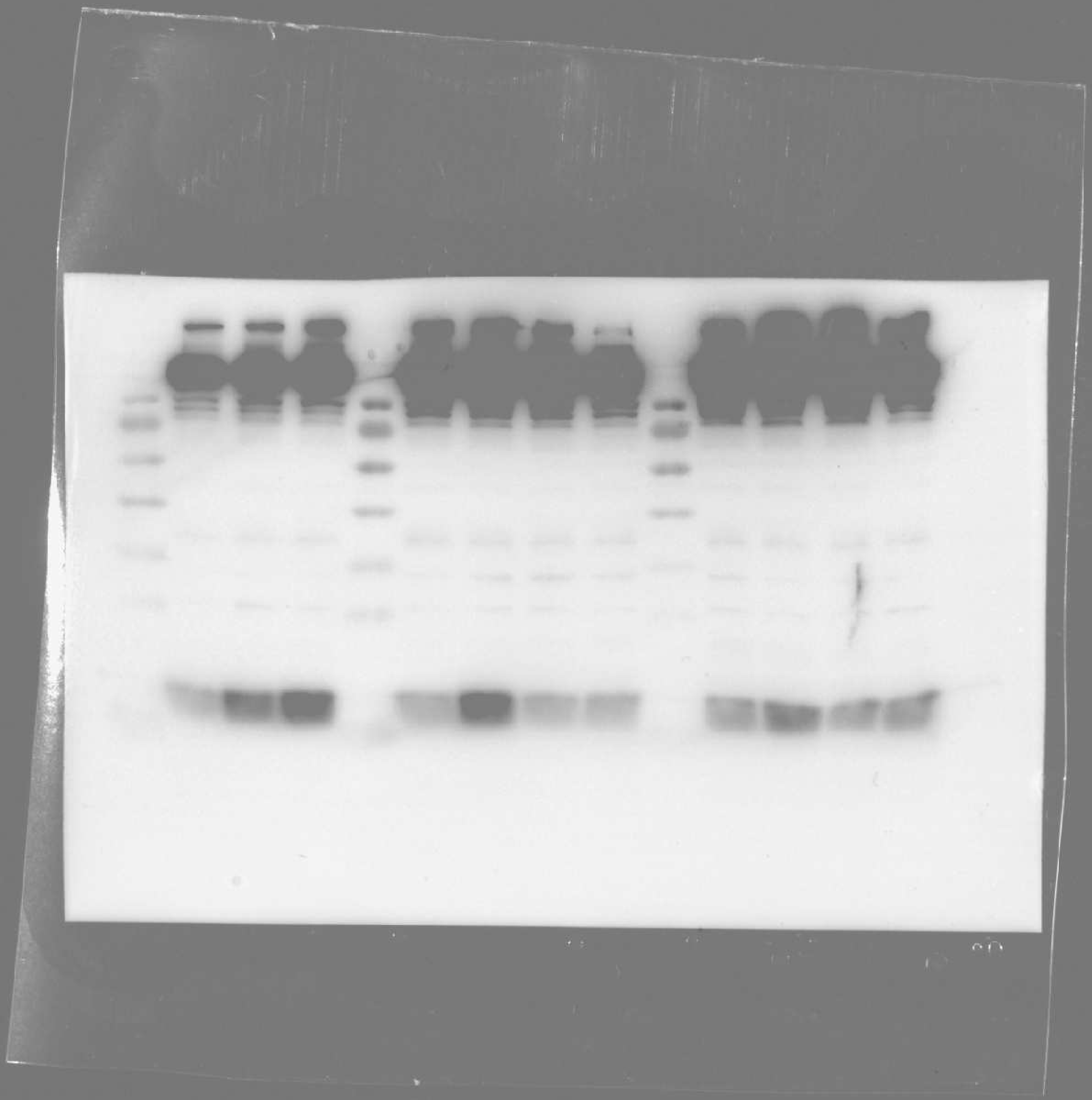

Figure_3A_pY245 cAbl

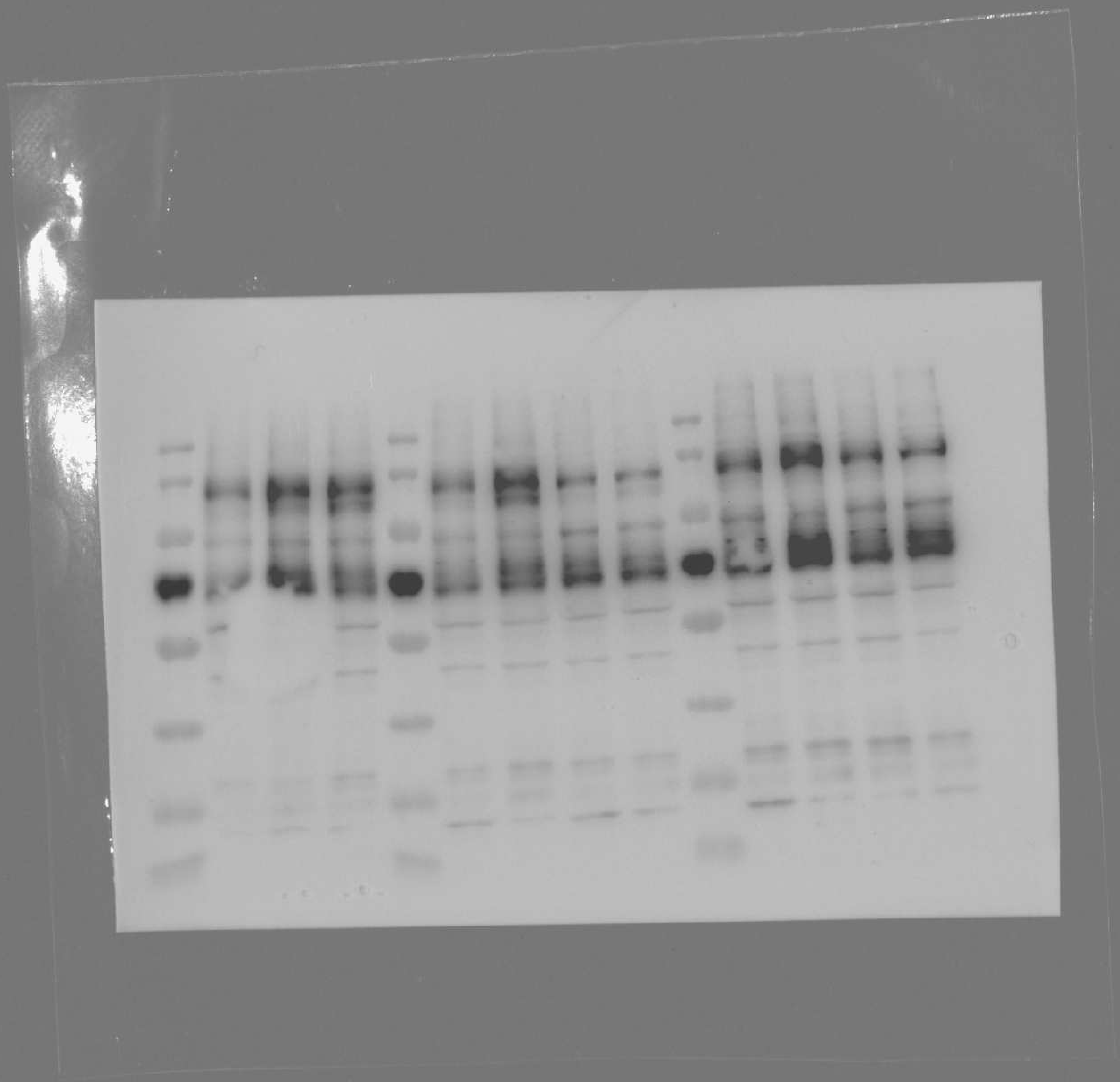

Figure_3A_pY412 cAbl

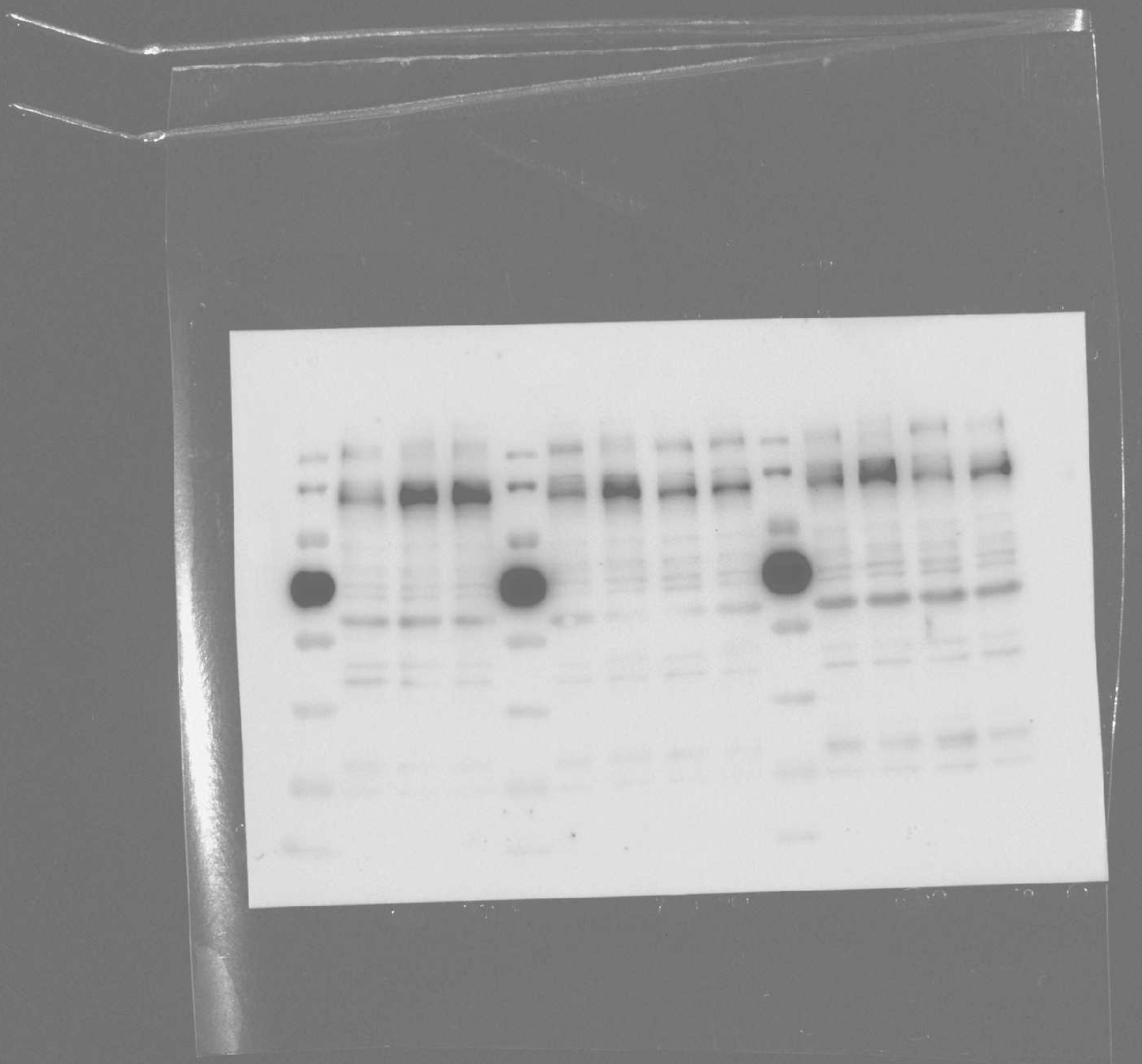

Figure_3A_total cAbl

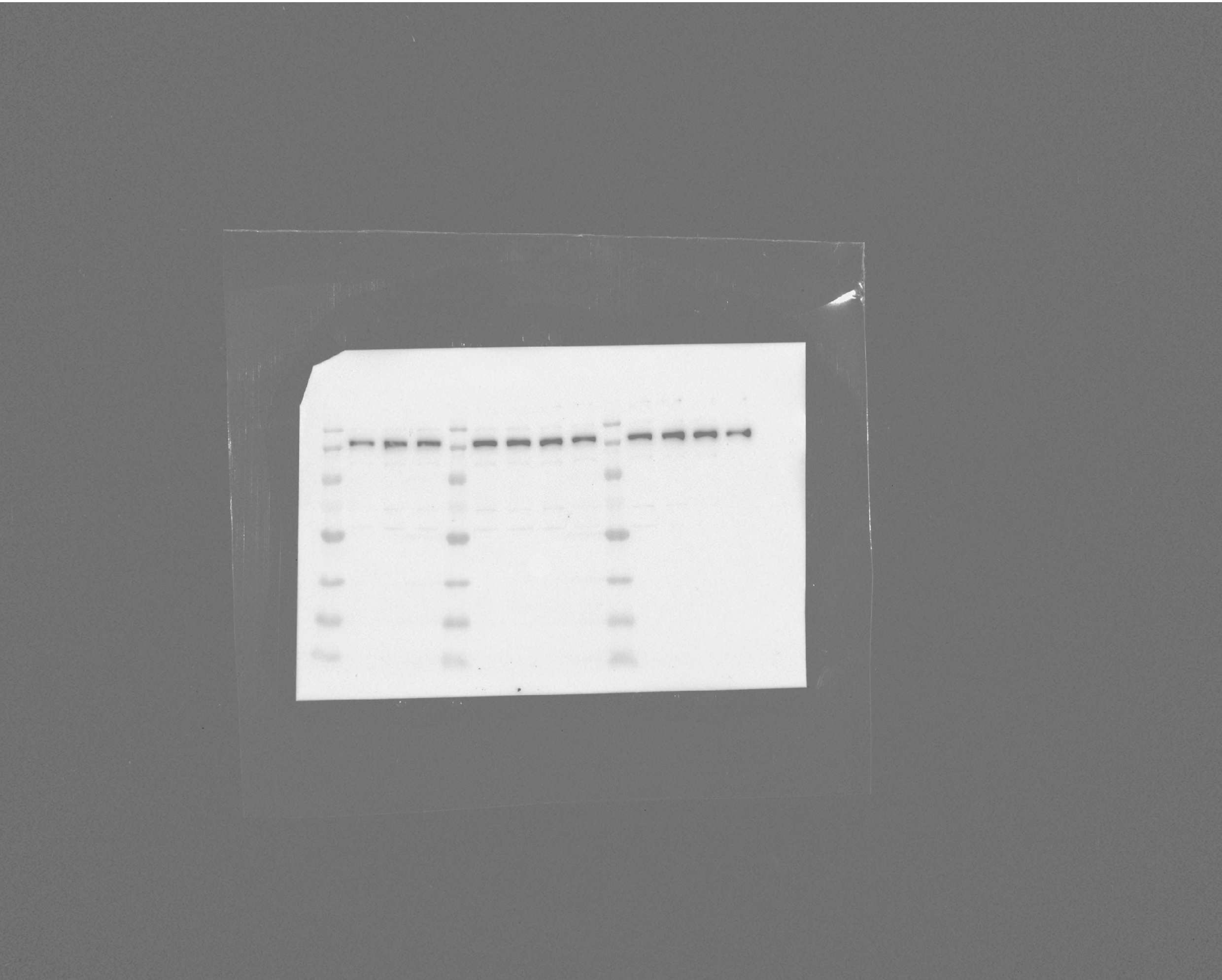

Figure_3A_total_a-synuclein

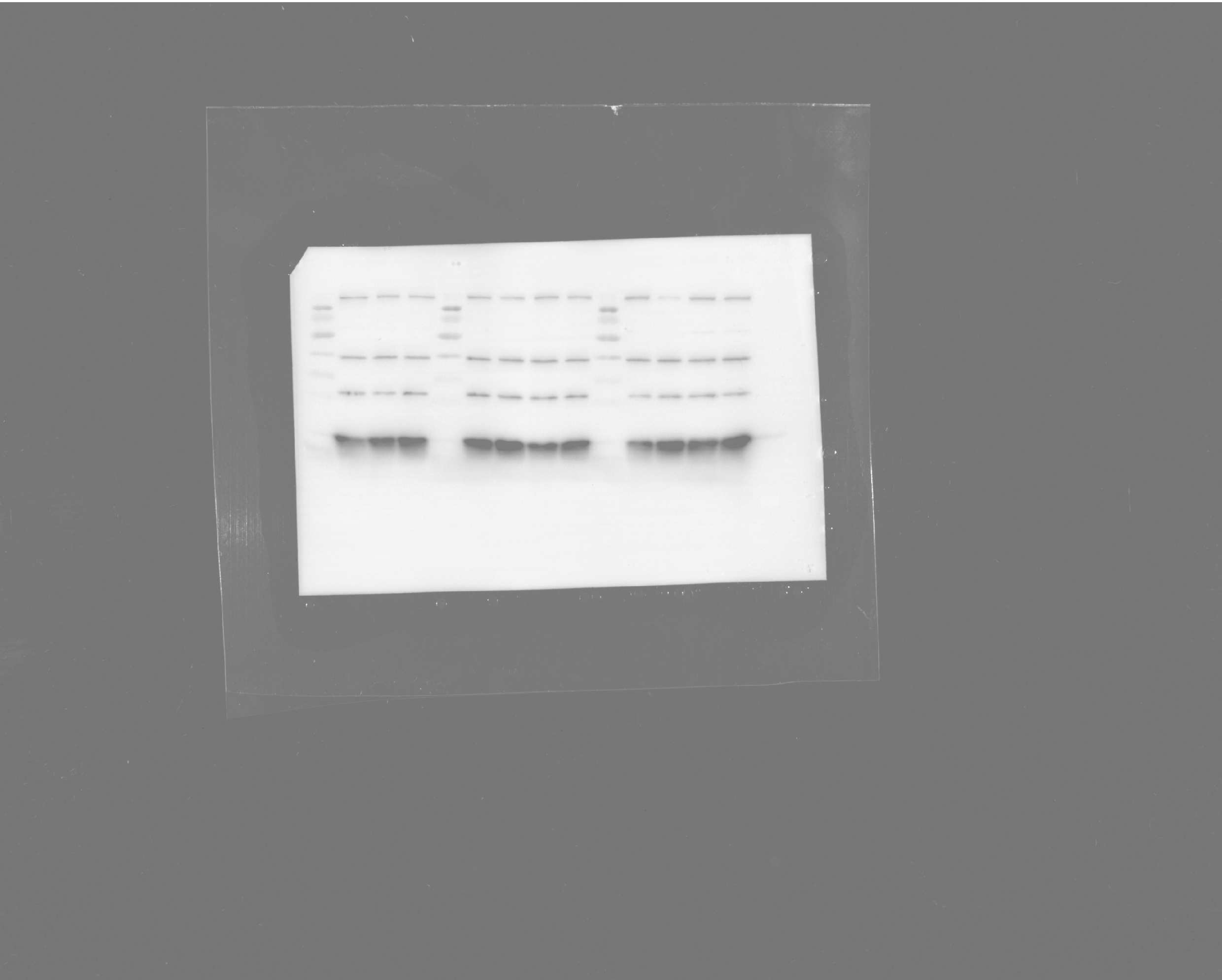

Figure_3B_beta Actin

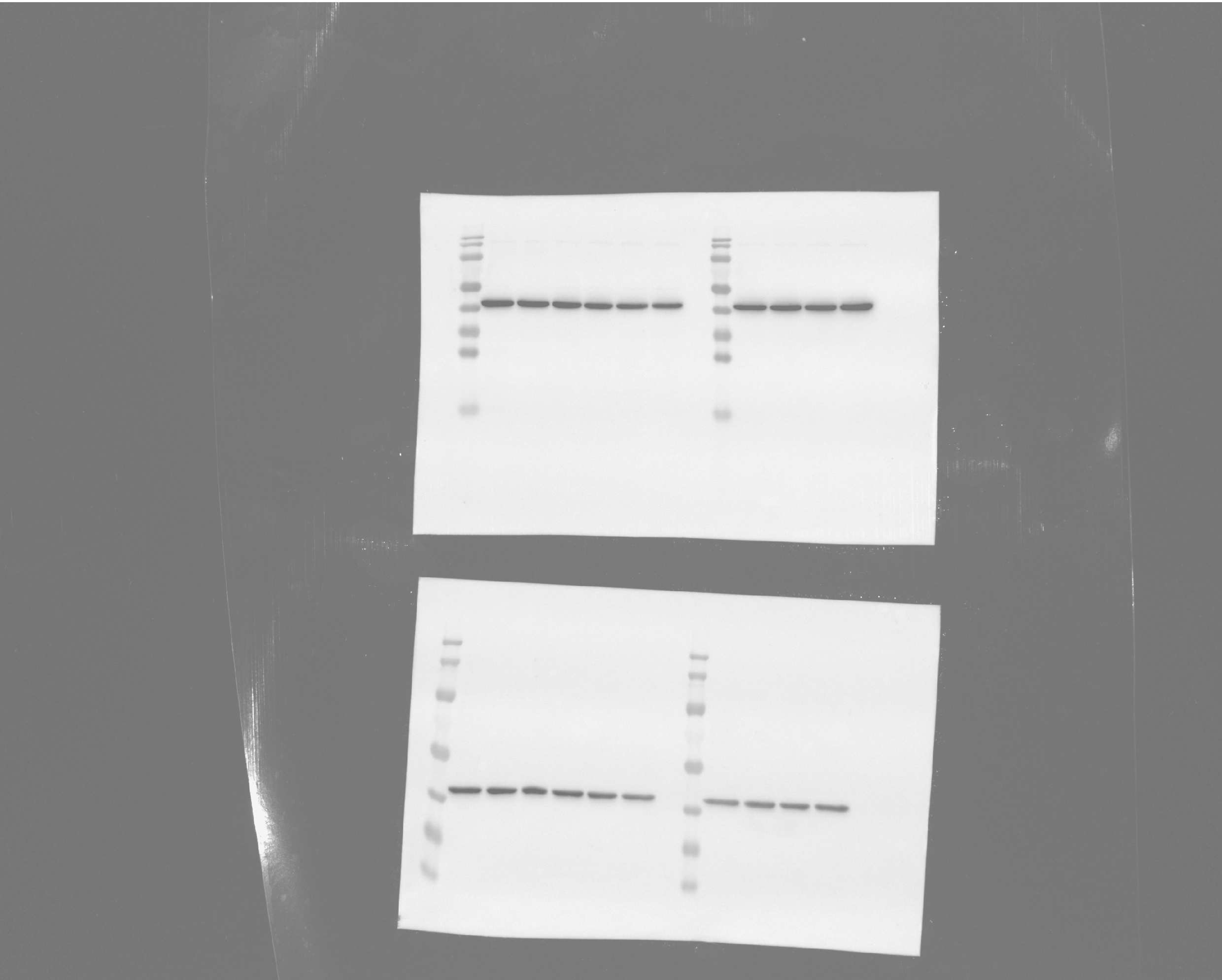

Figure_3B_pS129 a-Synuclein

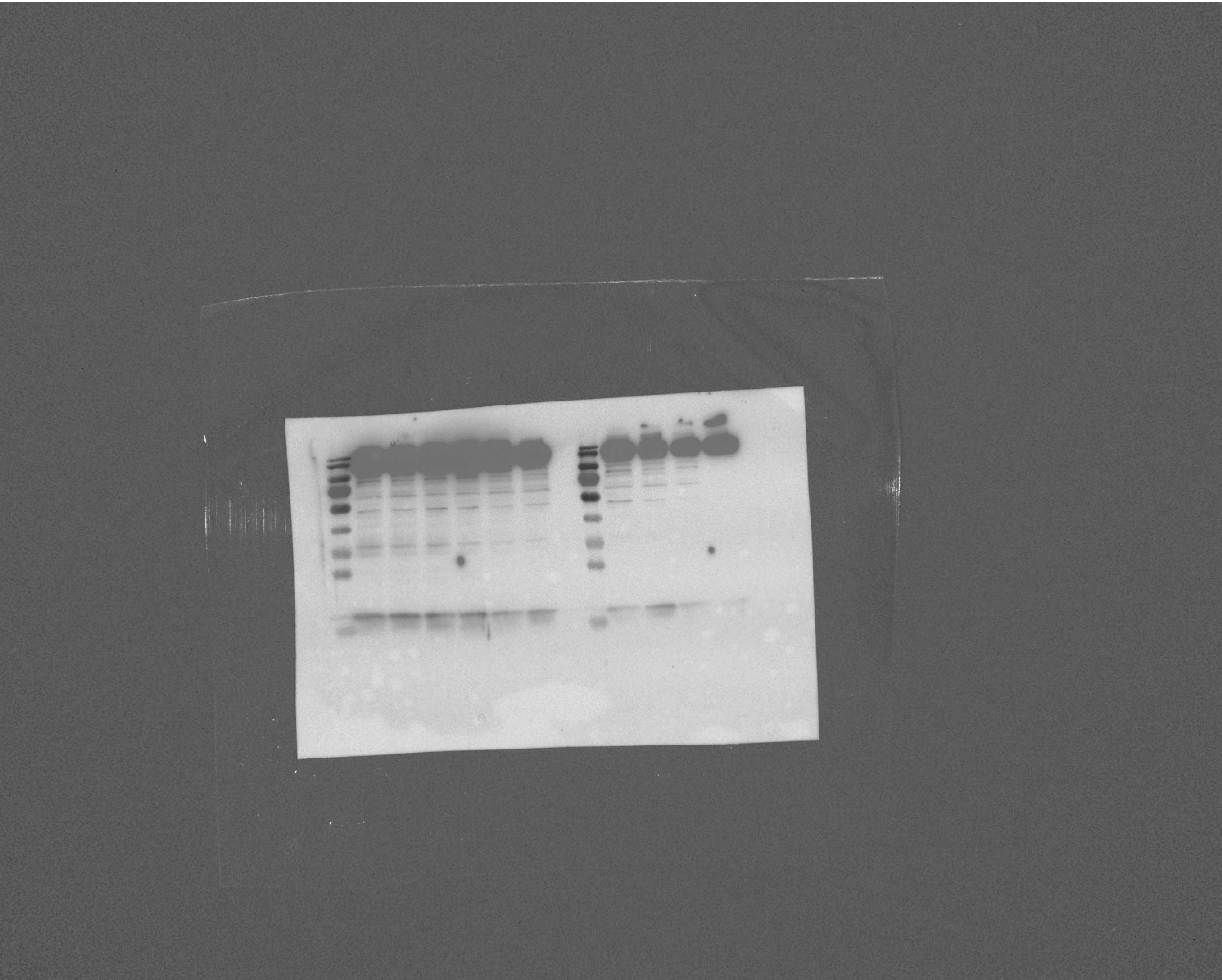

Figure_3B_pY245 cAbl

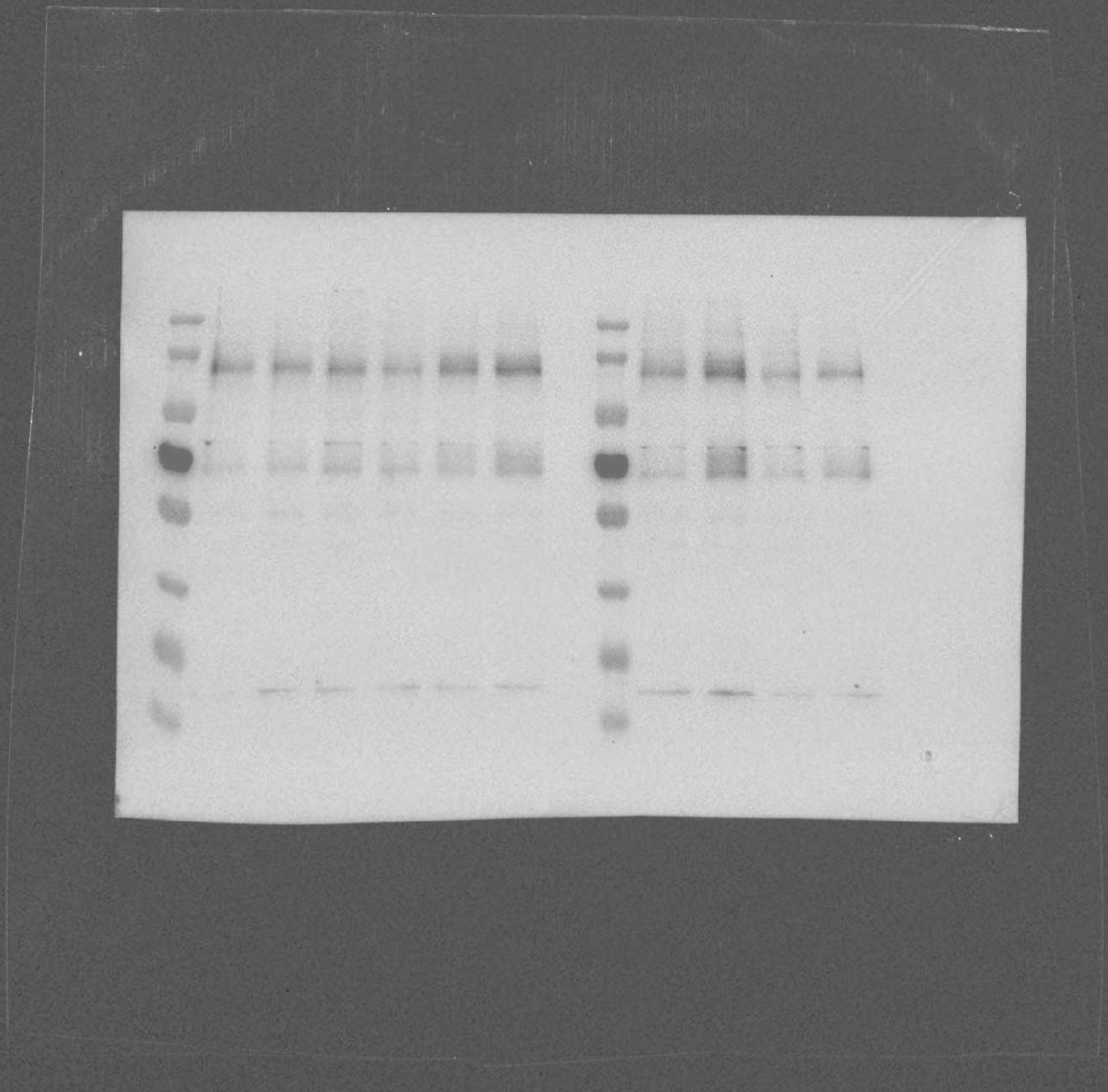

Figure_3B_pY412 cAbl

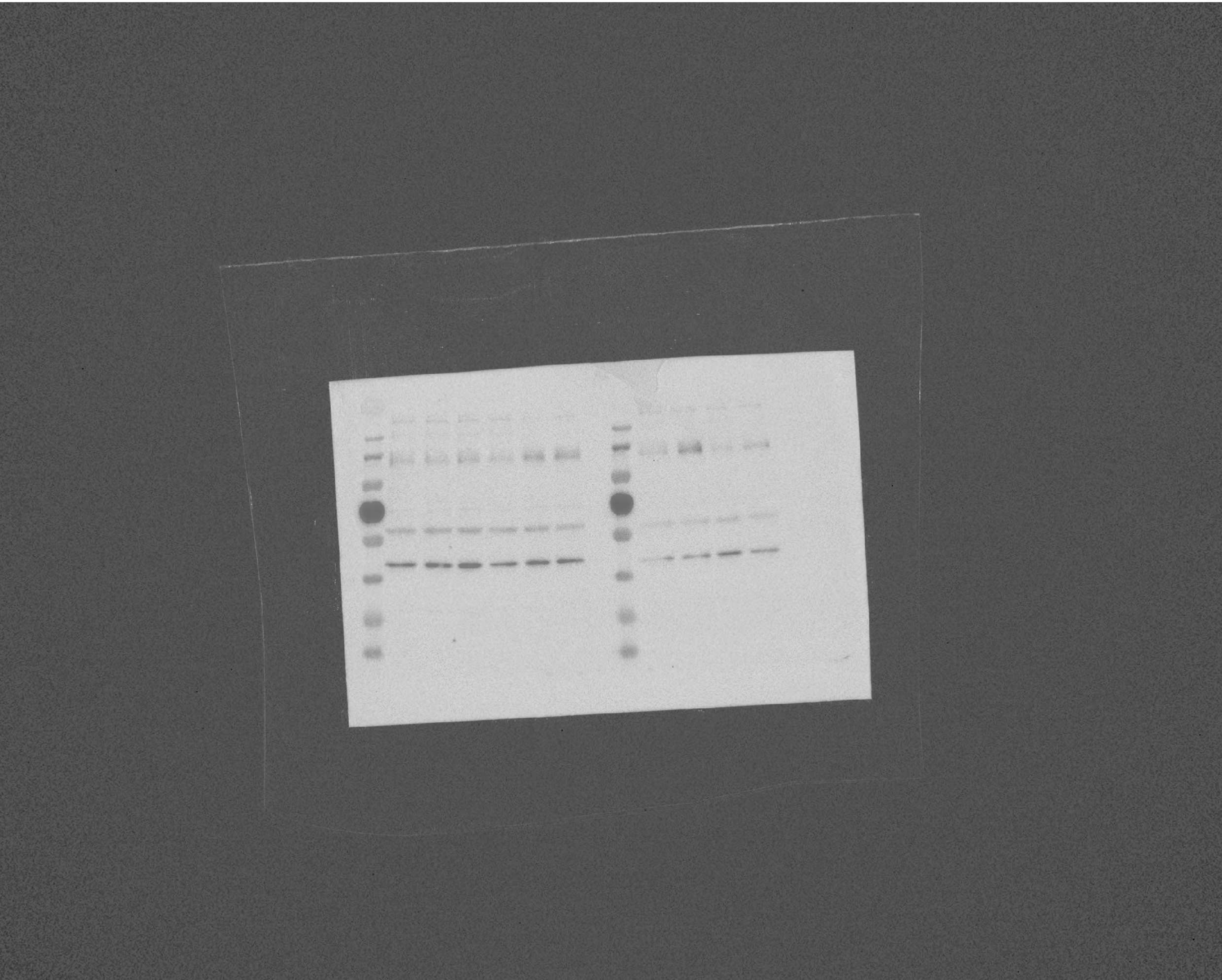

Figure_3B_total a-Synuclein

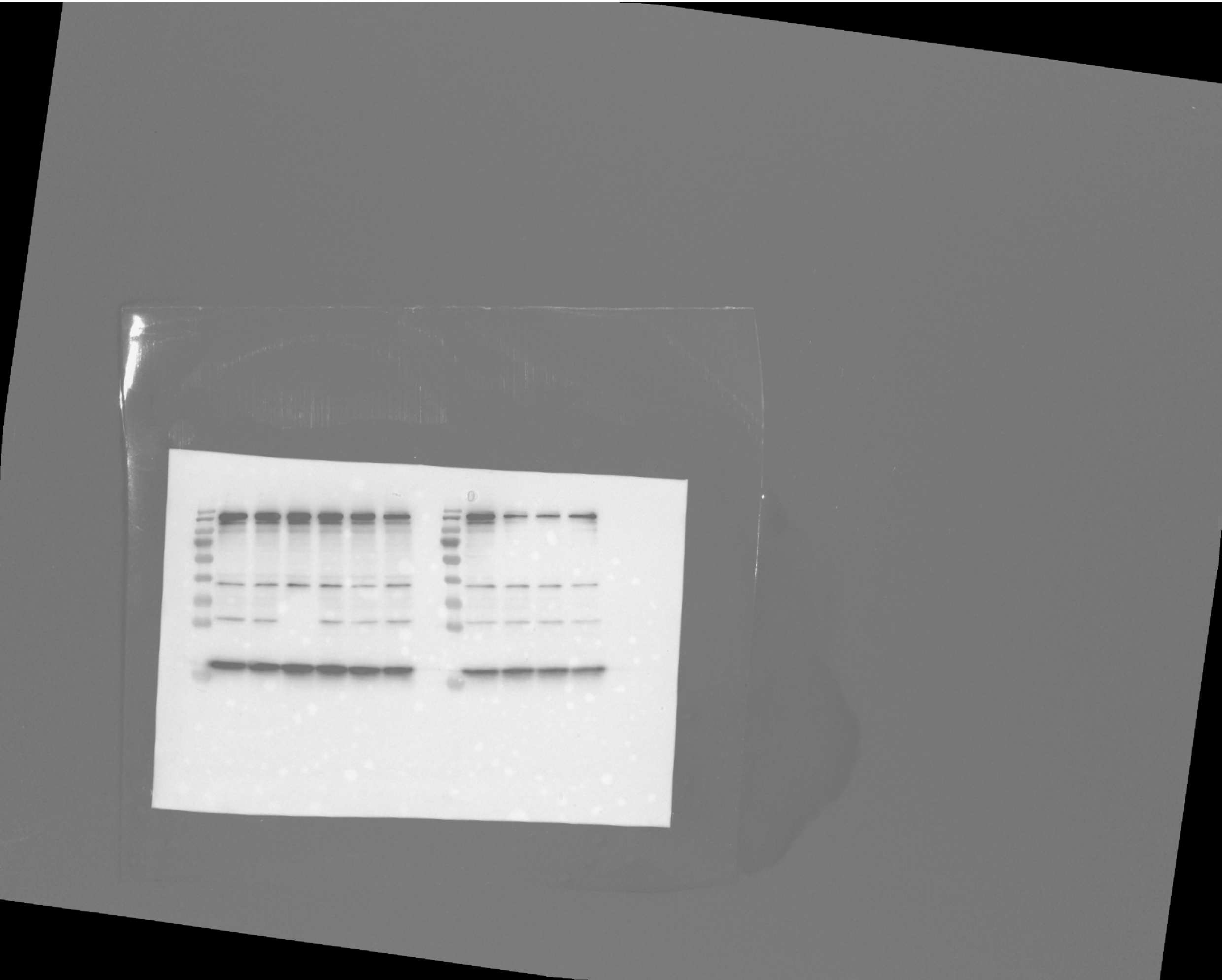

Figure_3B_total cAbl

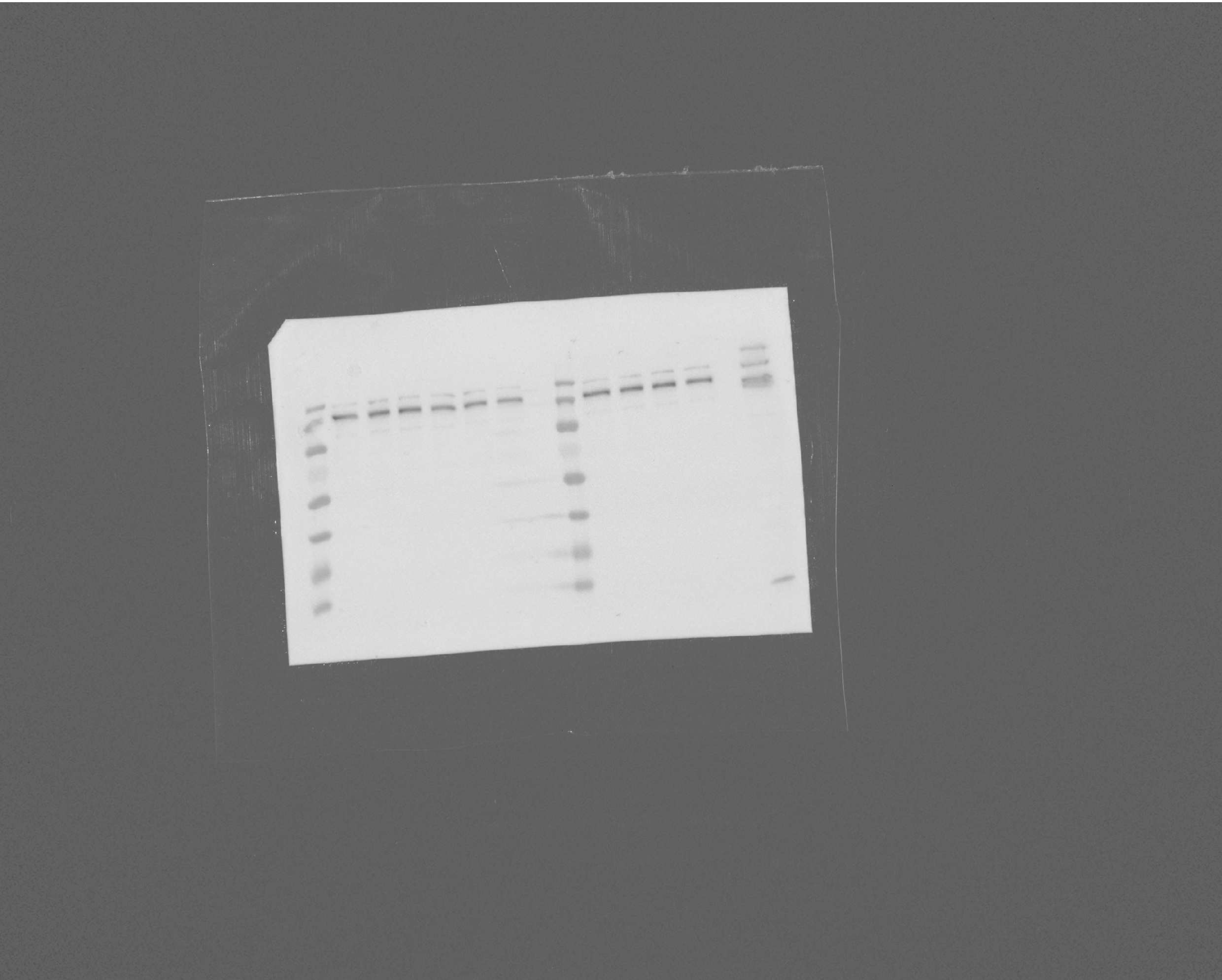

Figure_5C_beta_Actin

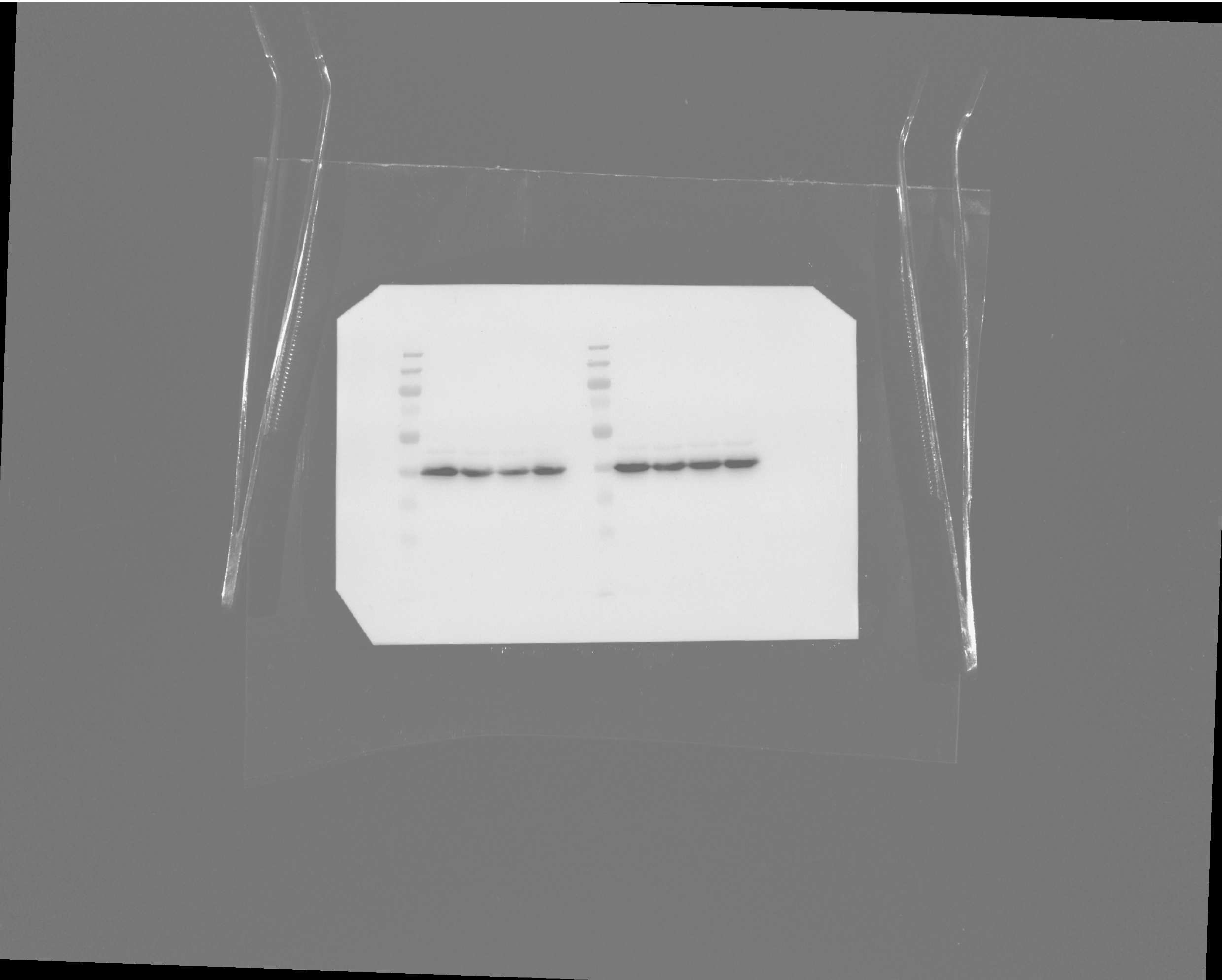

Figure_5C_p-Y216 GSK3B

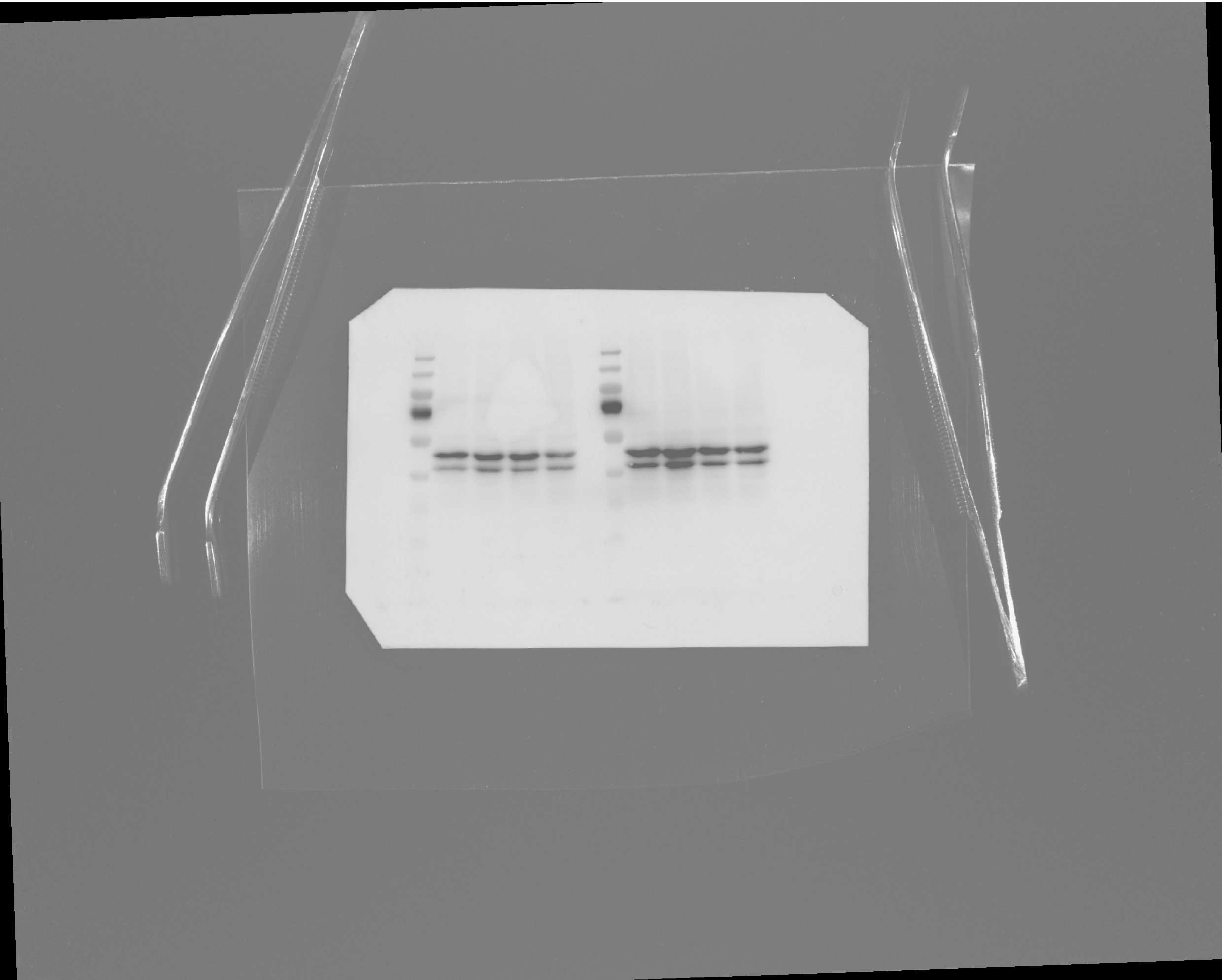

Figure_5C_total GSK3B

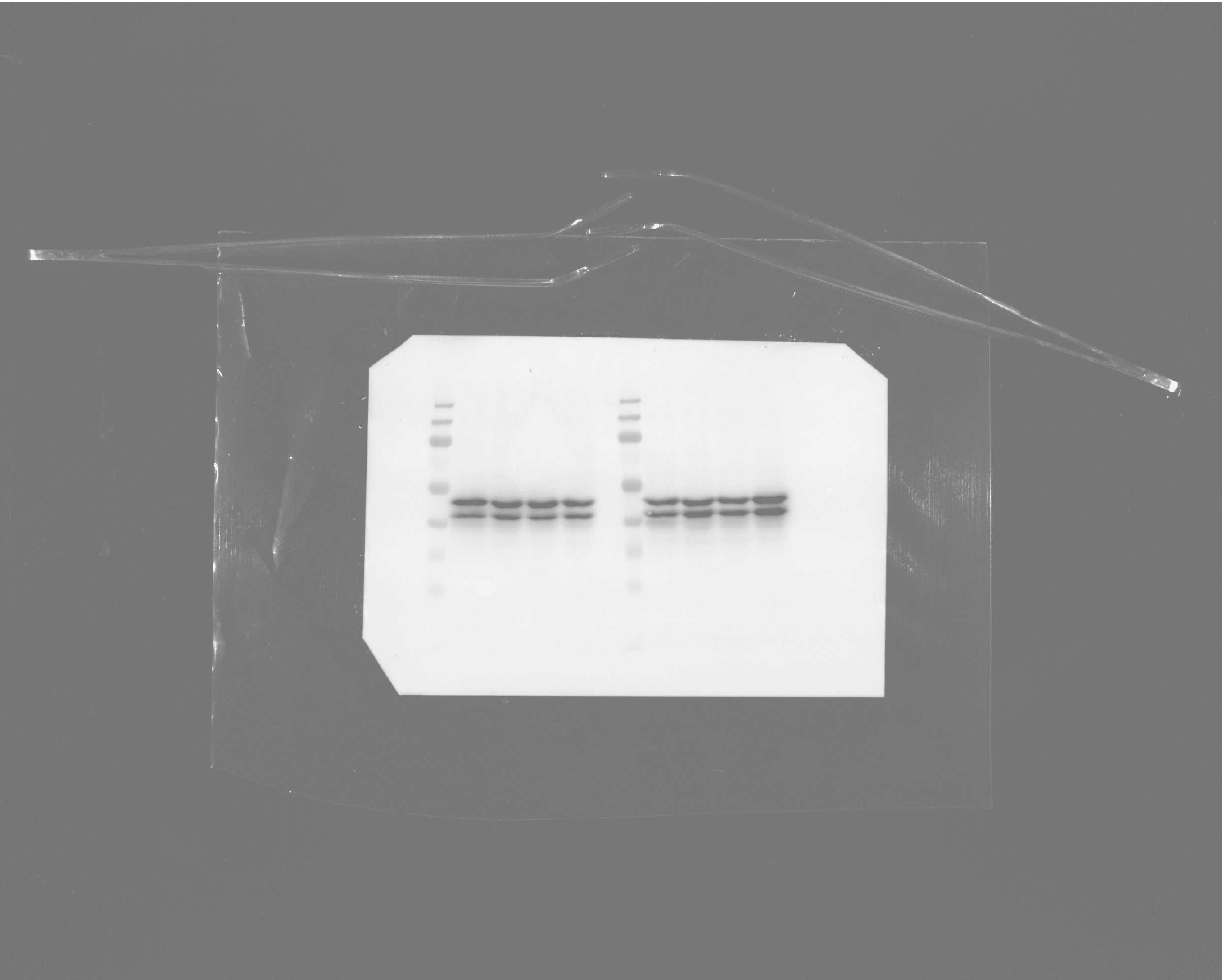

Figure_5D_beta_Actin

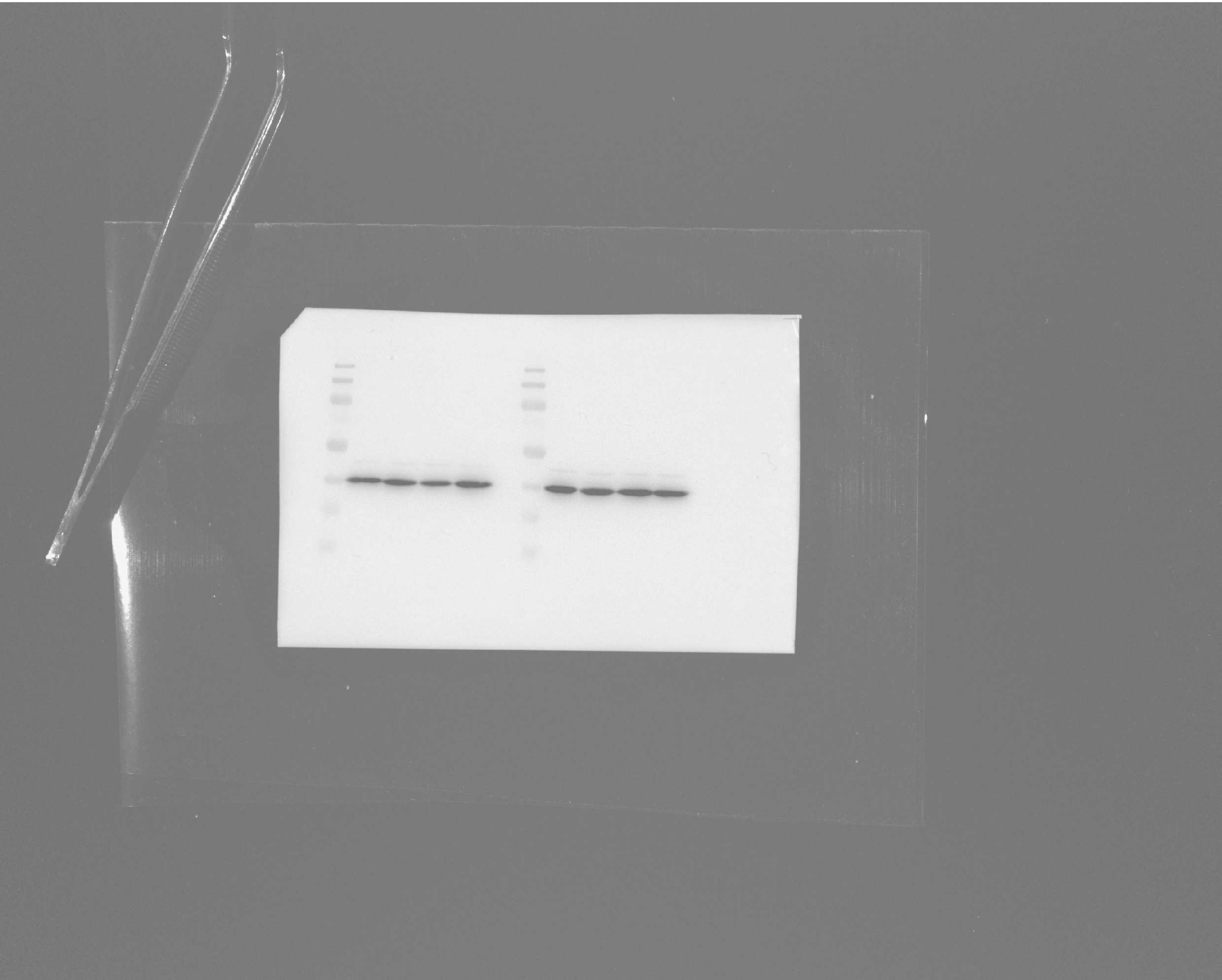

Figure_5D_pY216 GSK3B

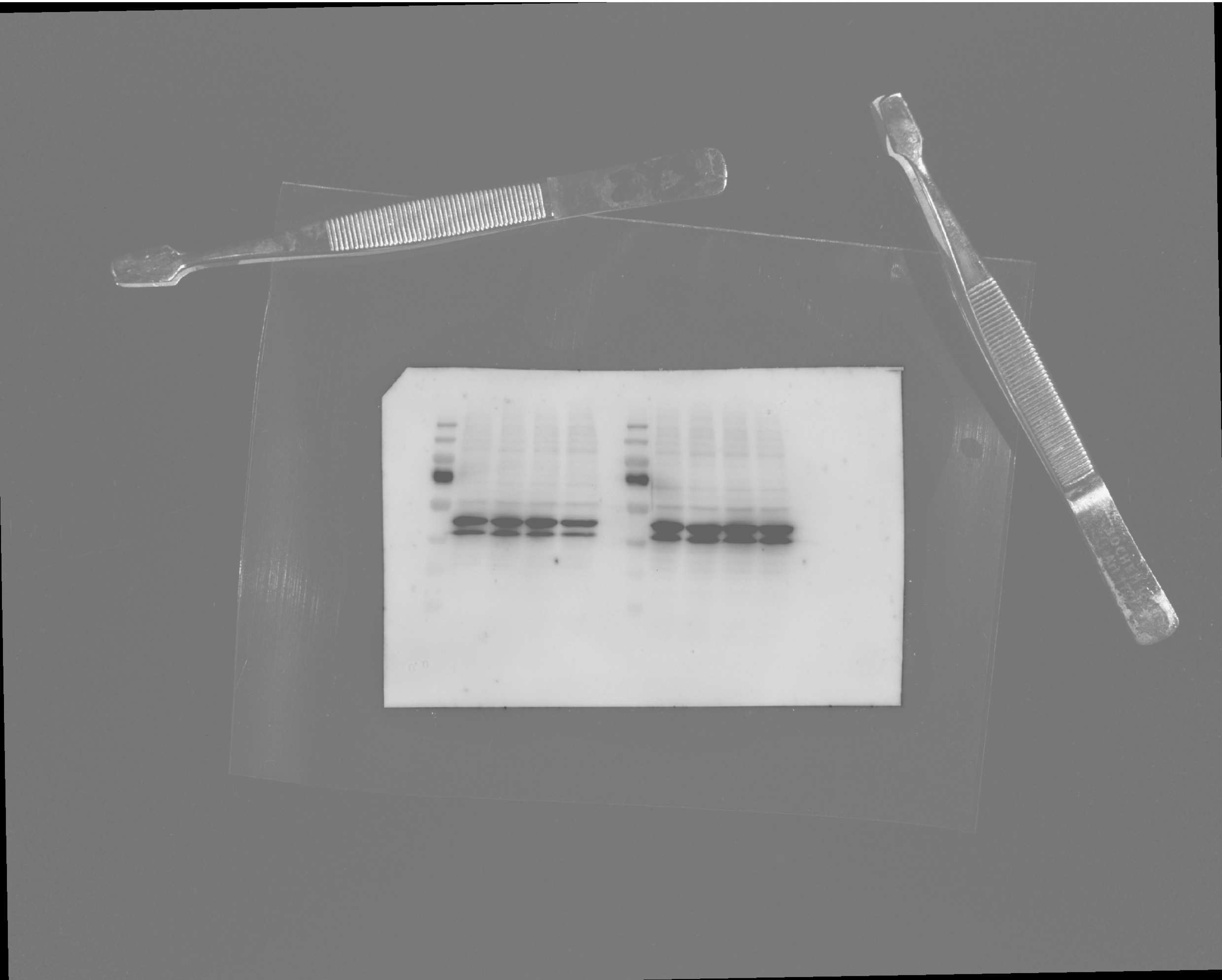

Figure_5D_total GSK3B

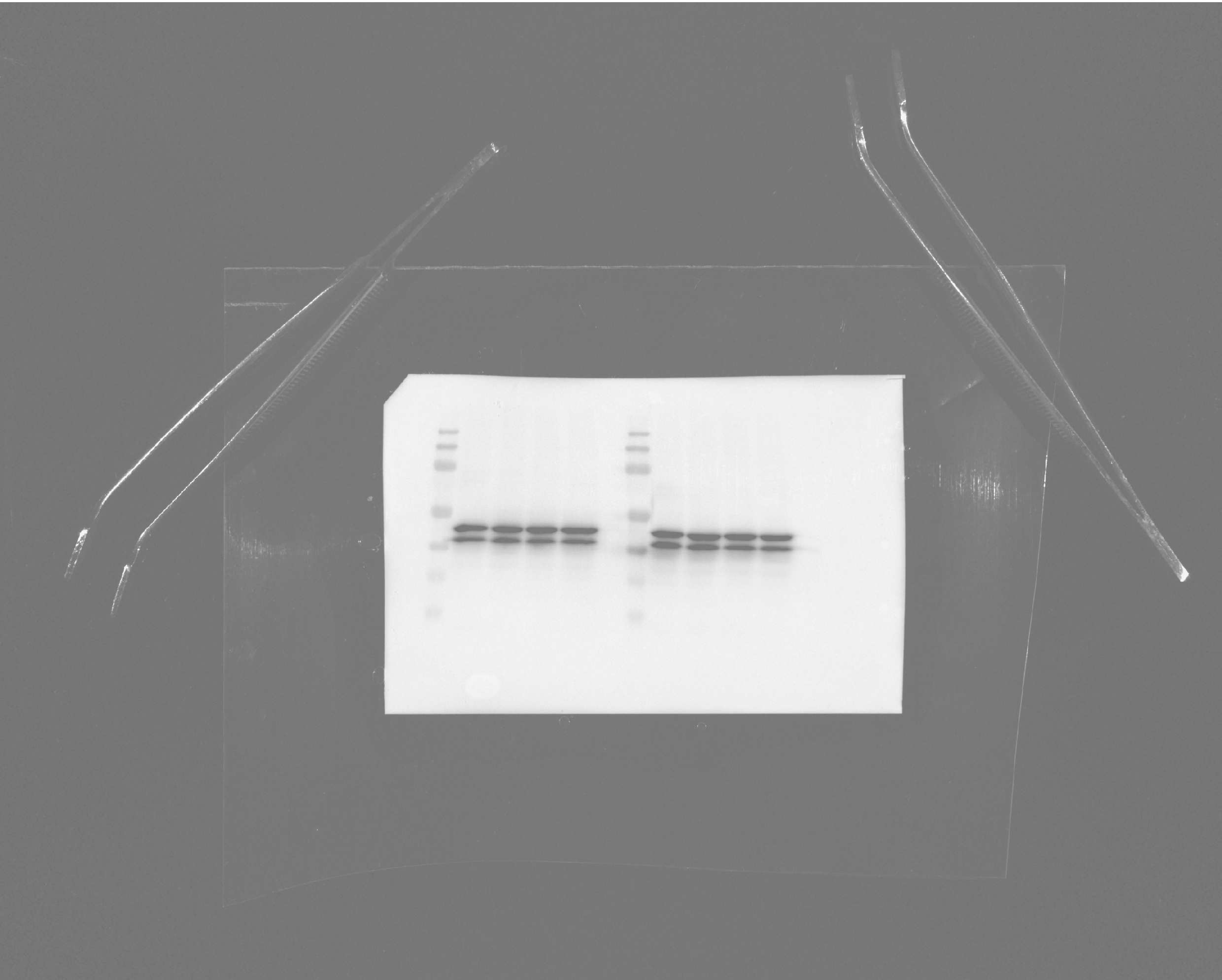

Figure_5E_beta_Actin

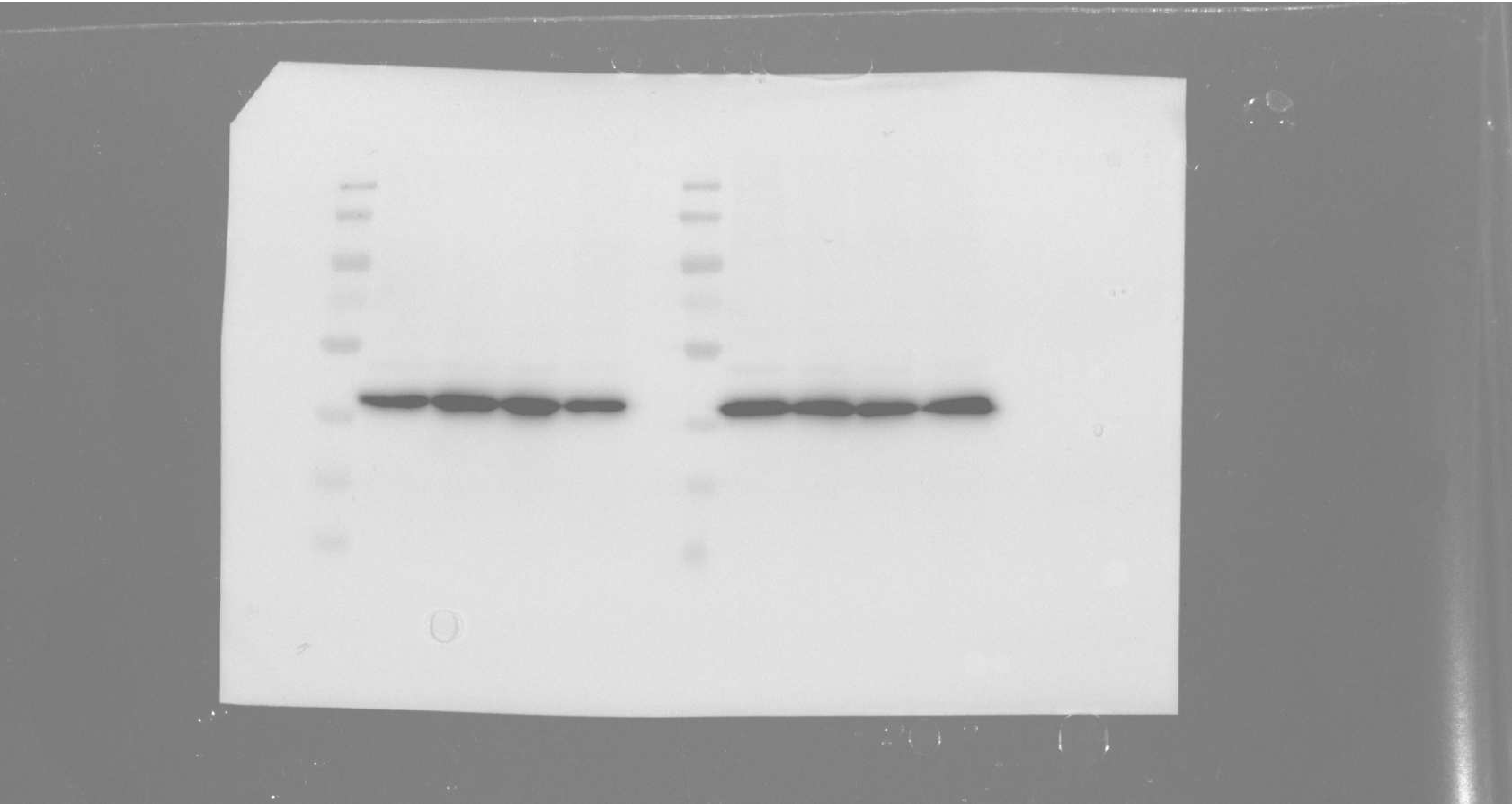

Figure_5E_pY216 GSK3B

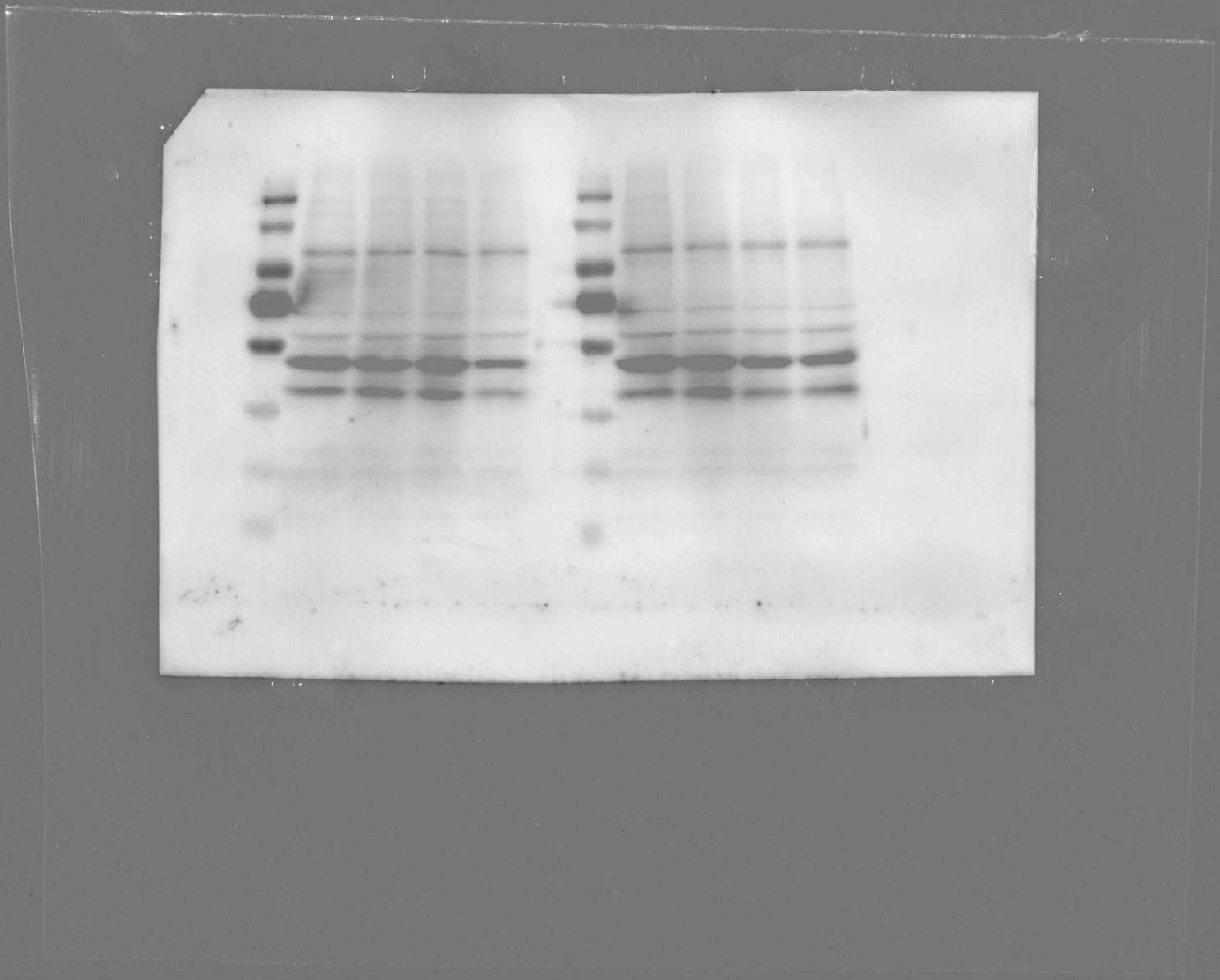

Figure_5E_total GSK3B

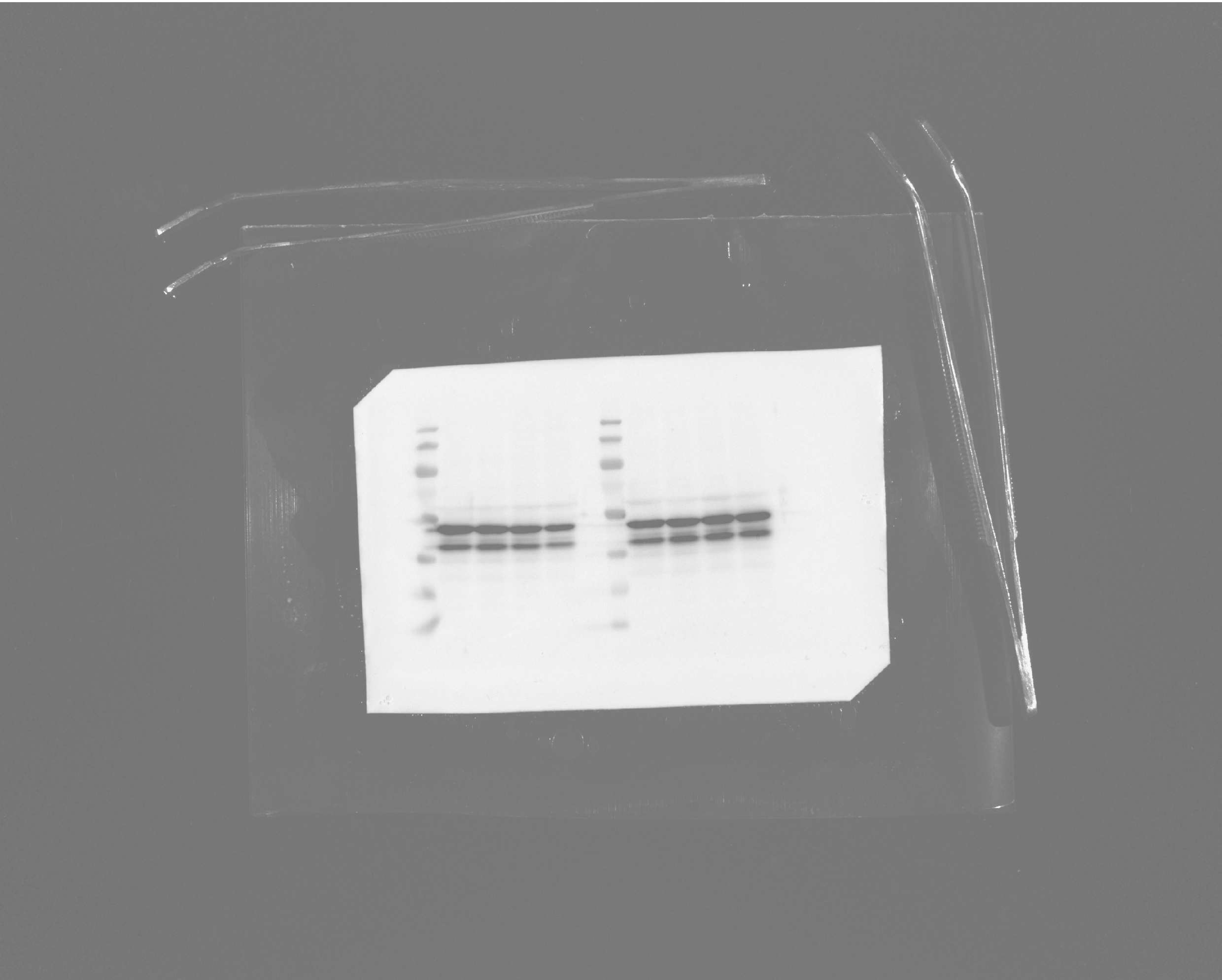

Figure_5F_beta_Actin

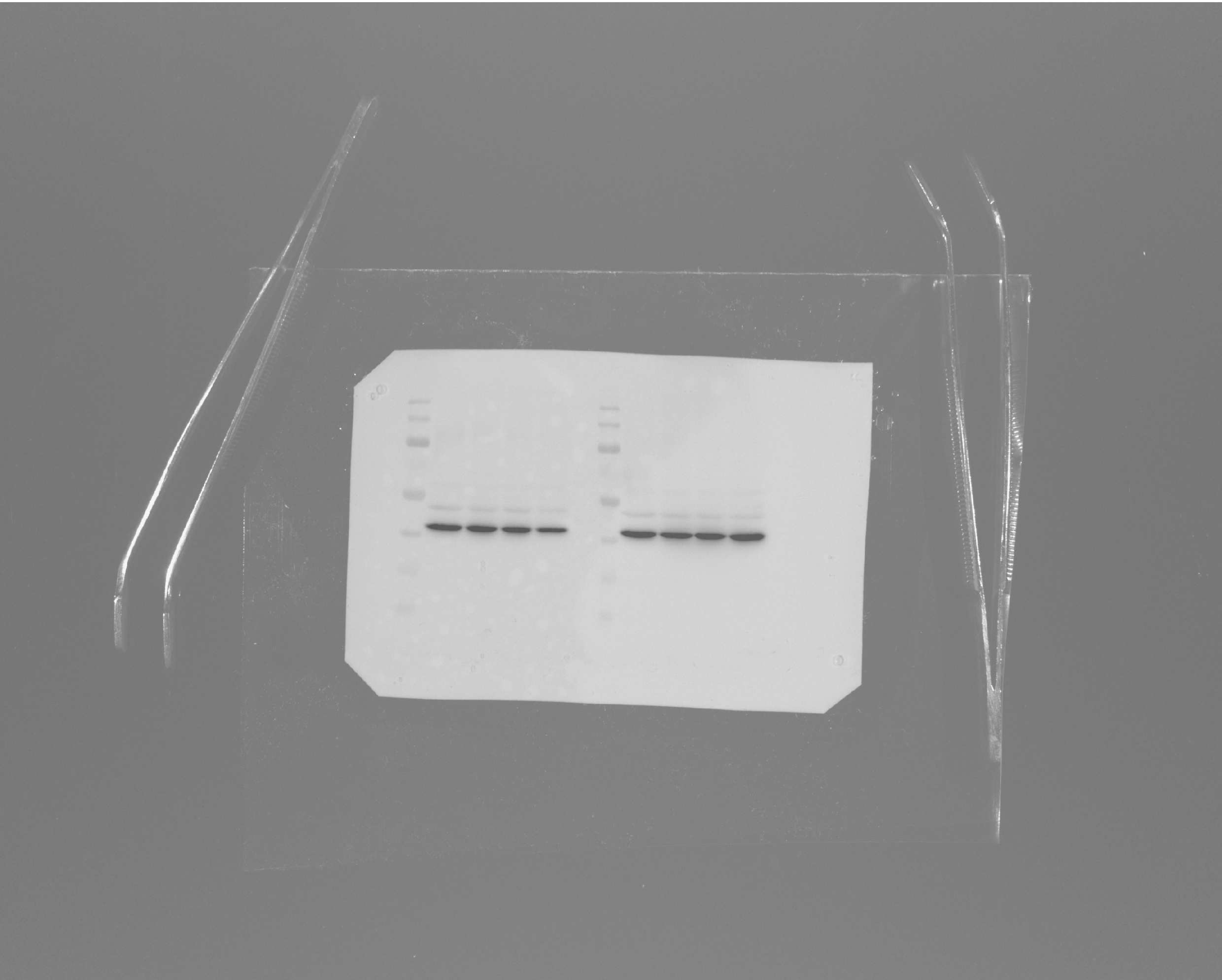

Figure_5F_pY216 GSK3B

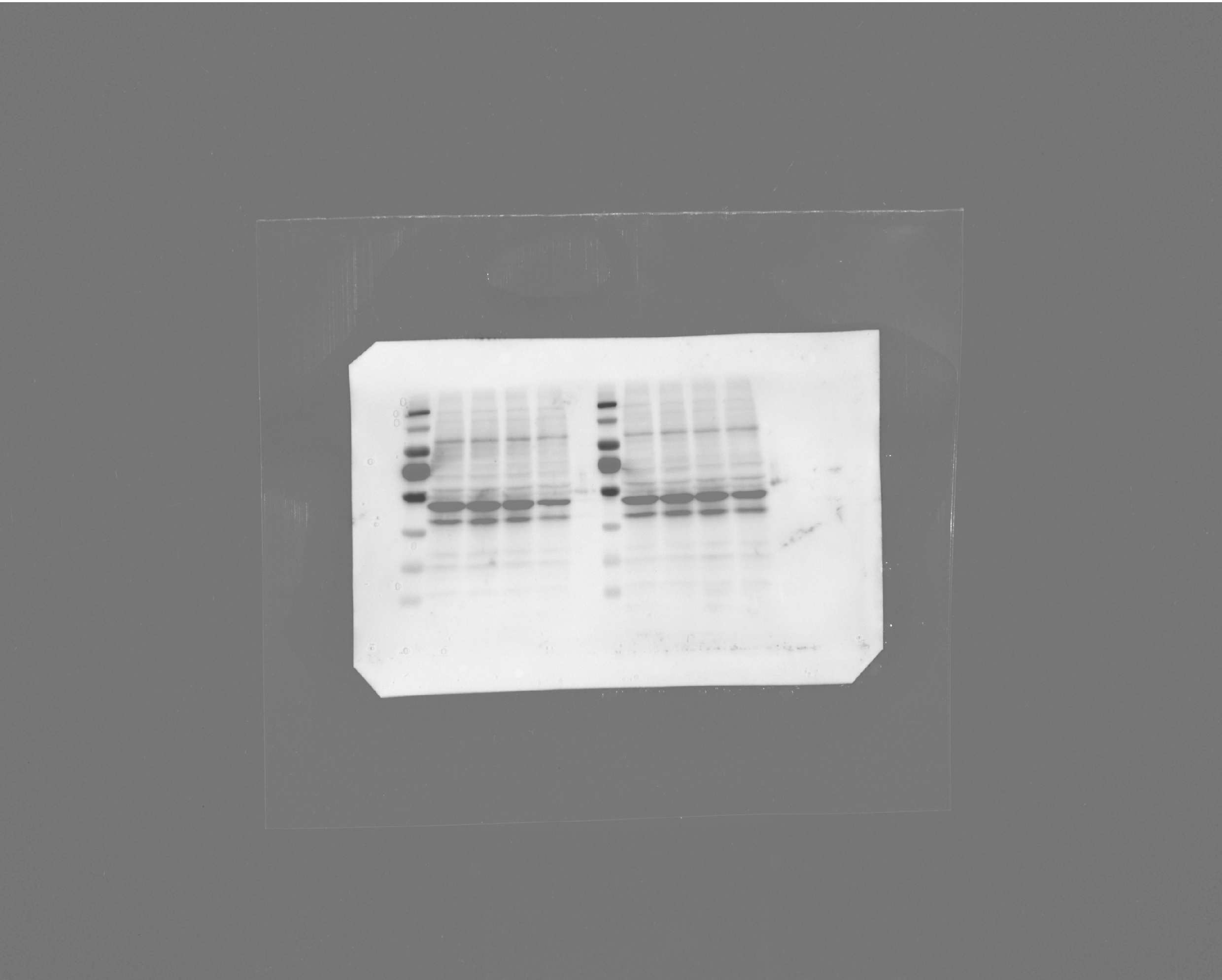

Figure_5F_total GSK3B

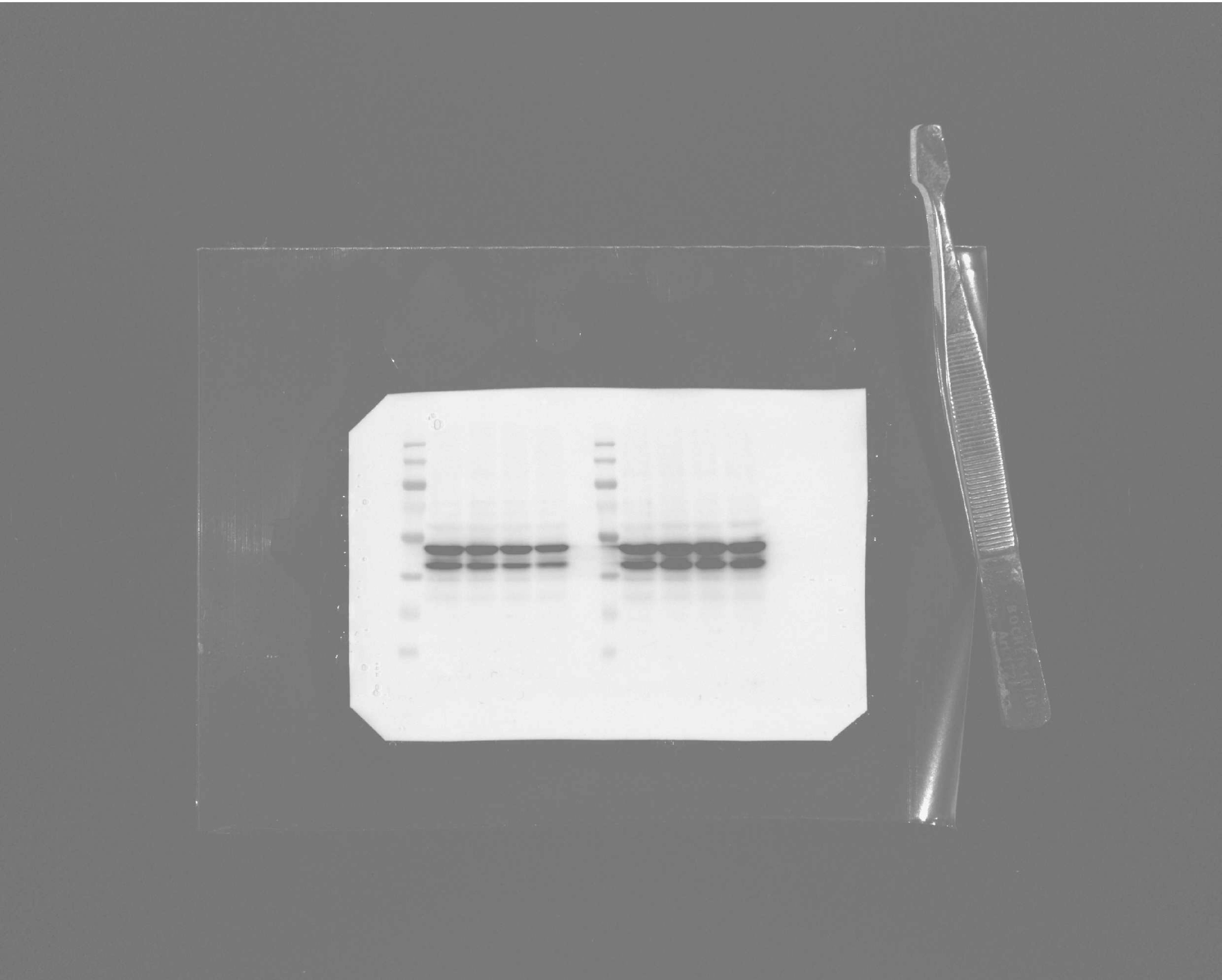

Figure_5G_beta_Actin

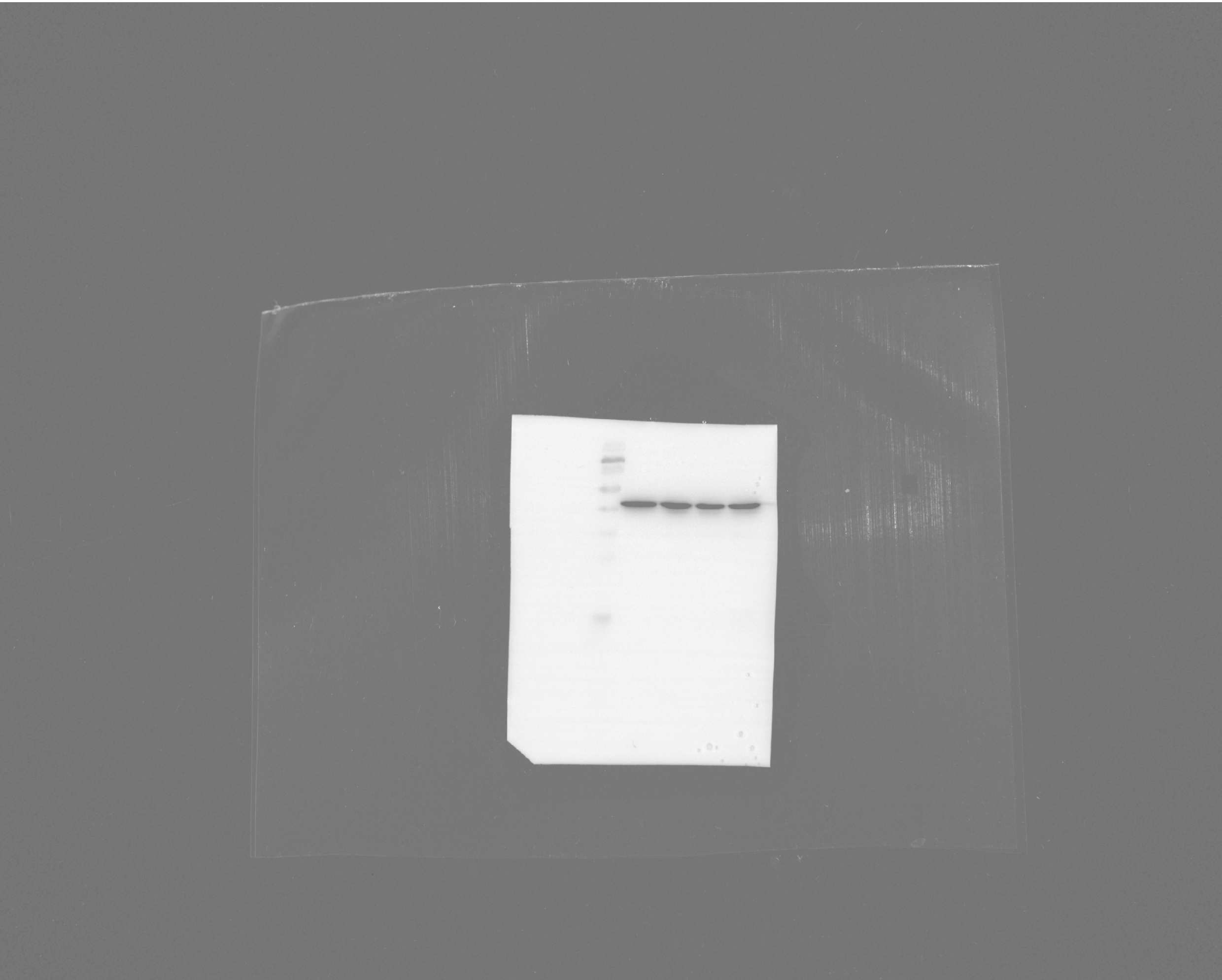

Figure_5G_pS129 a-synuclein

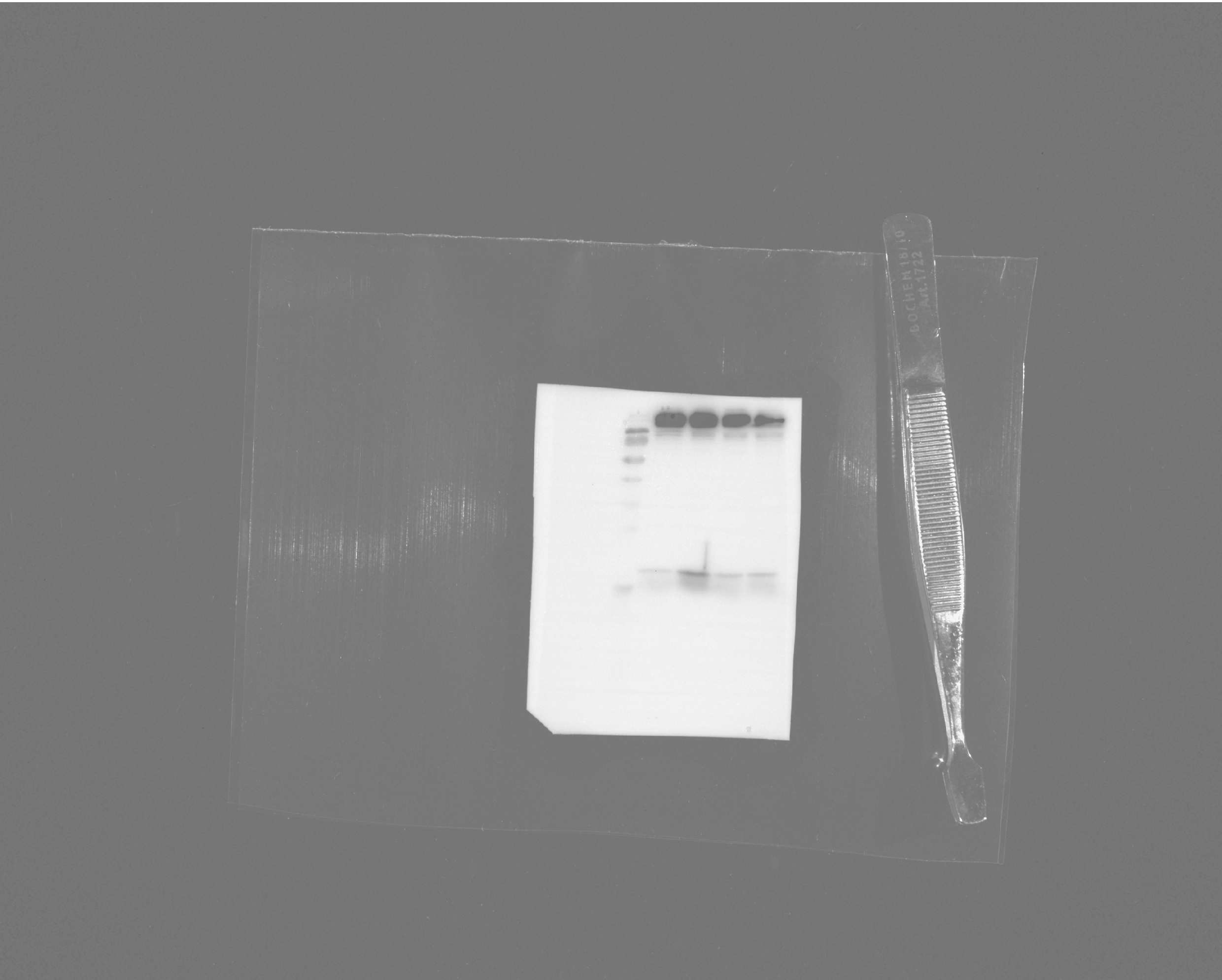

Figure_5G_pY216 GSk3B

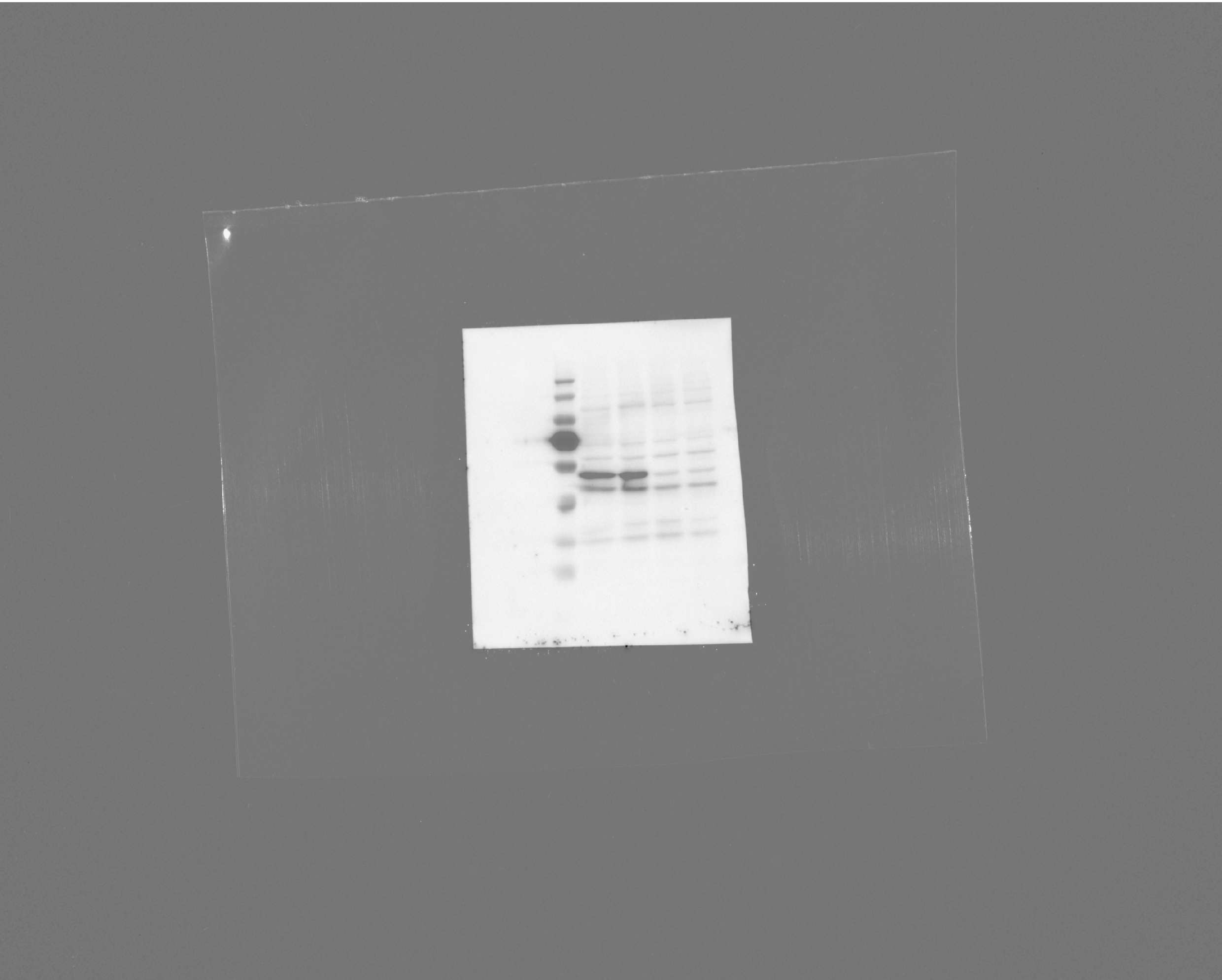

Figure_5G_total a-synuclein

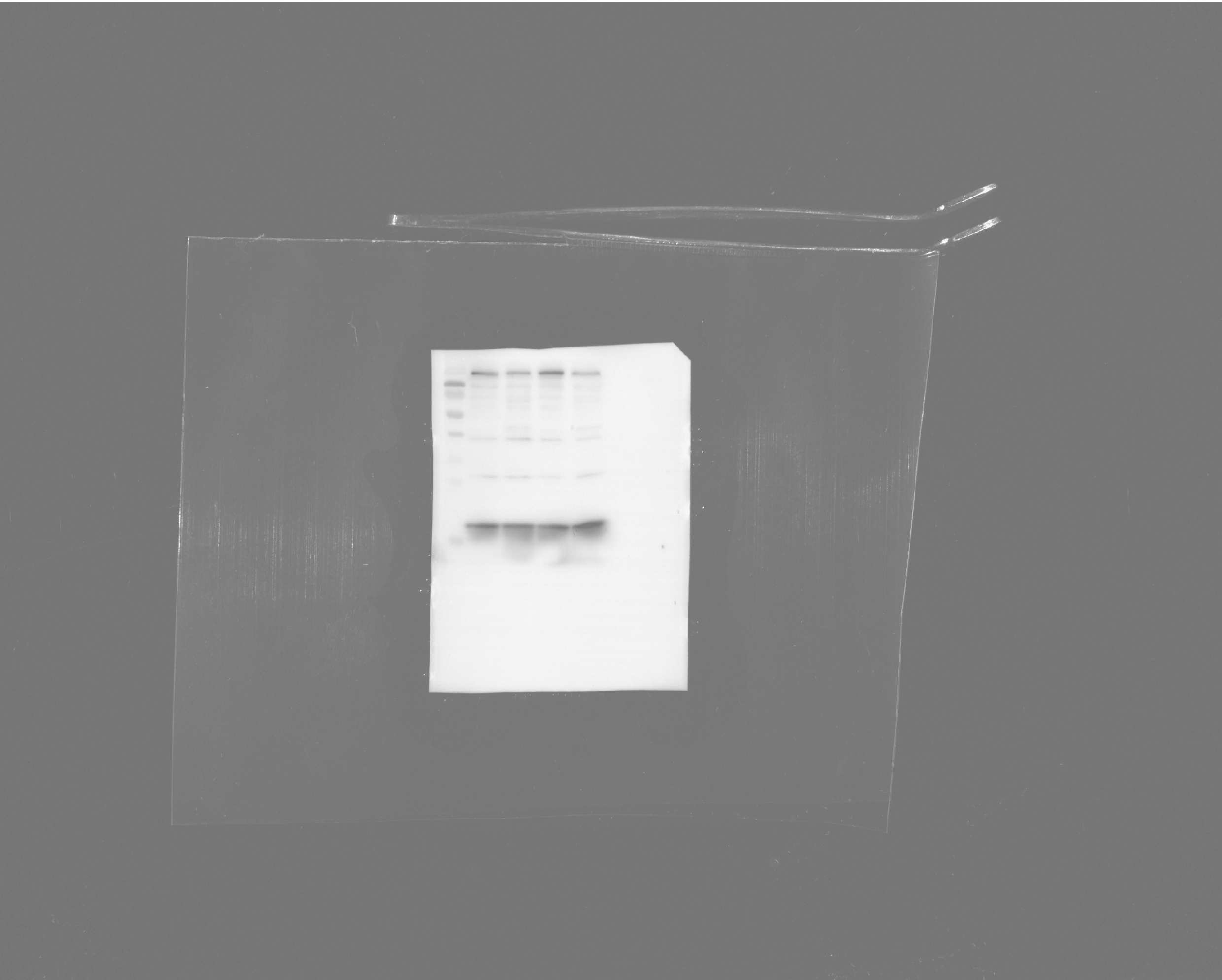

Figure_5G_total GSk3B

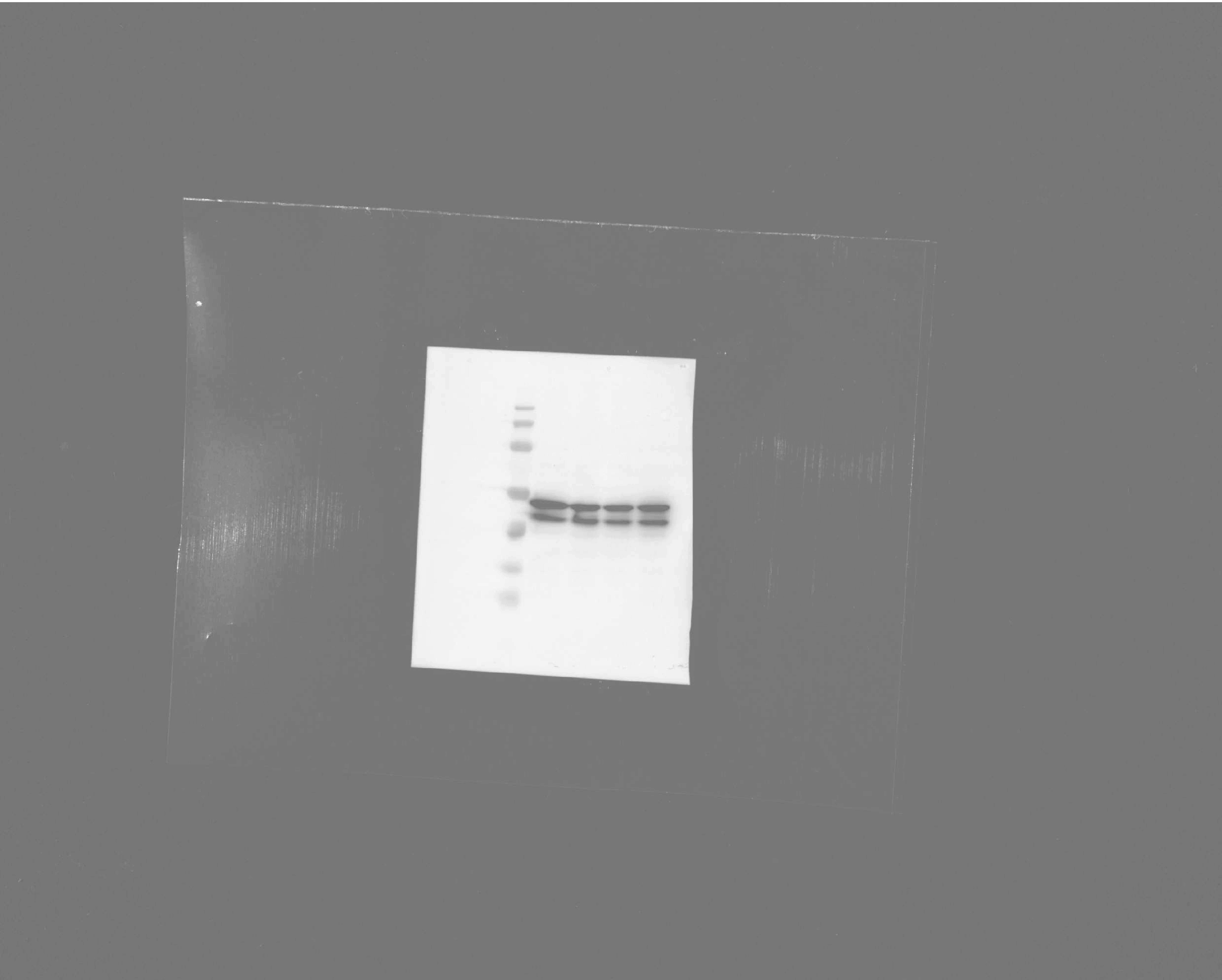

Figure_5H_beta_Actin

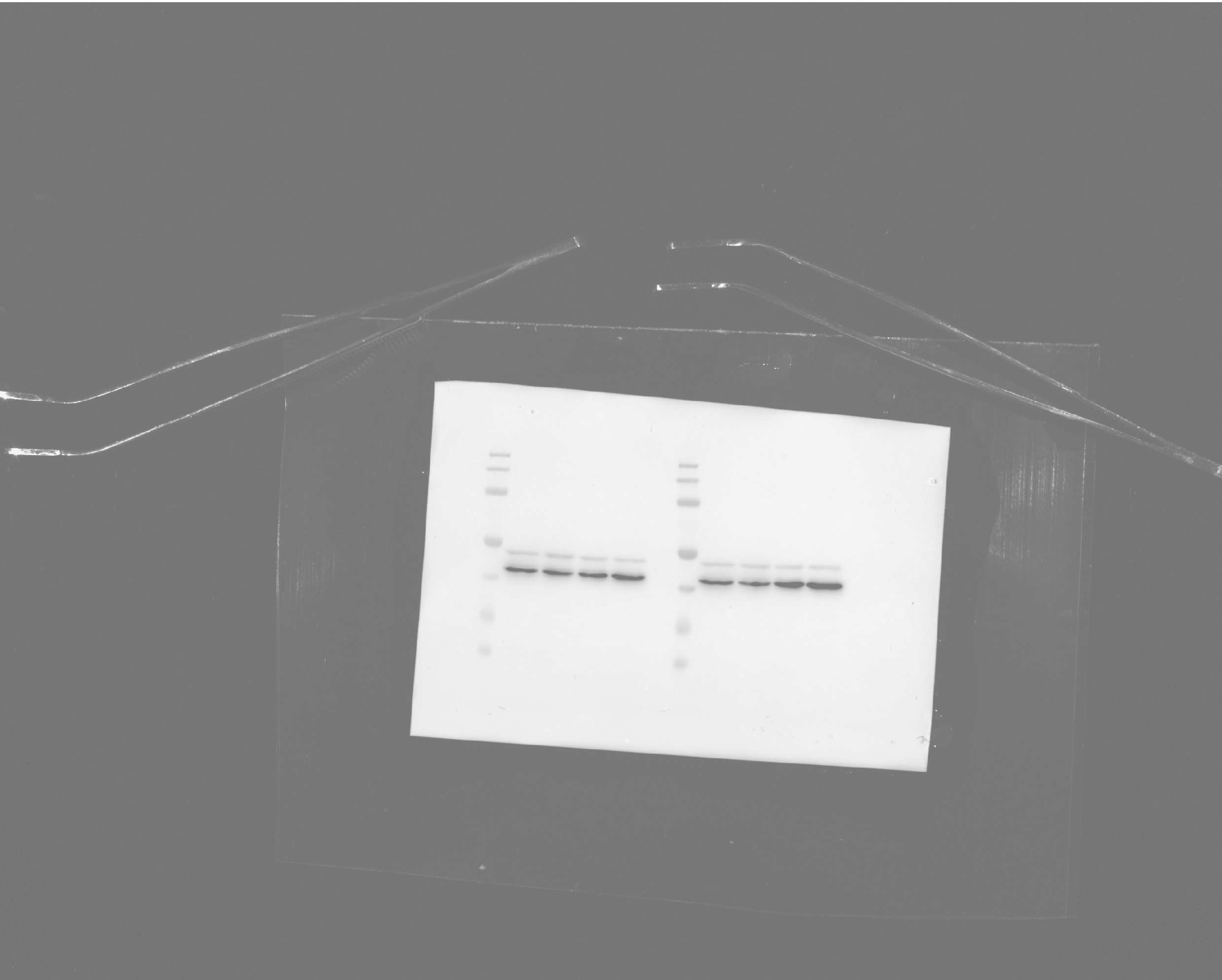

Figure_5H_pSer129 a-synuclein

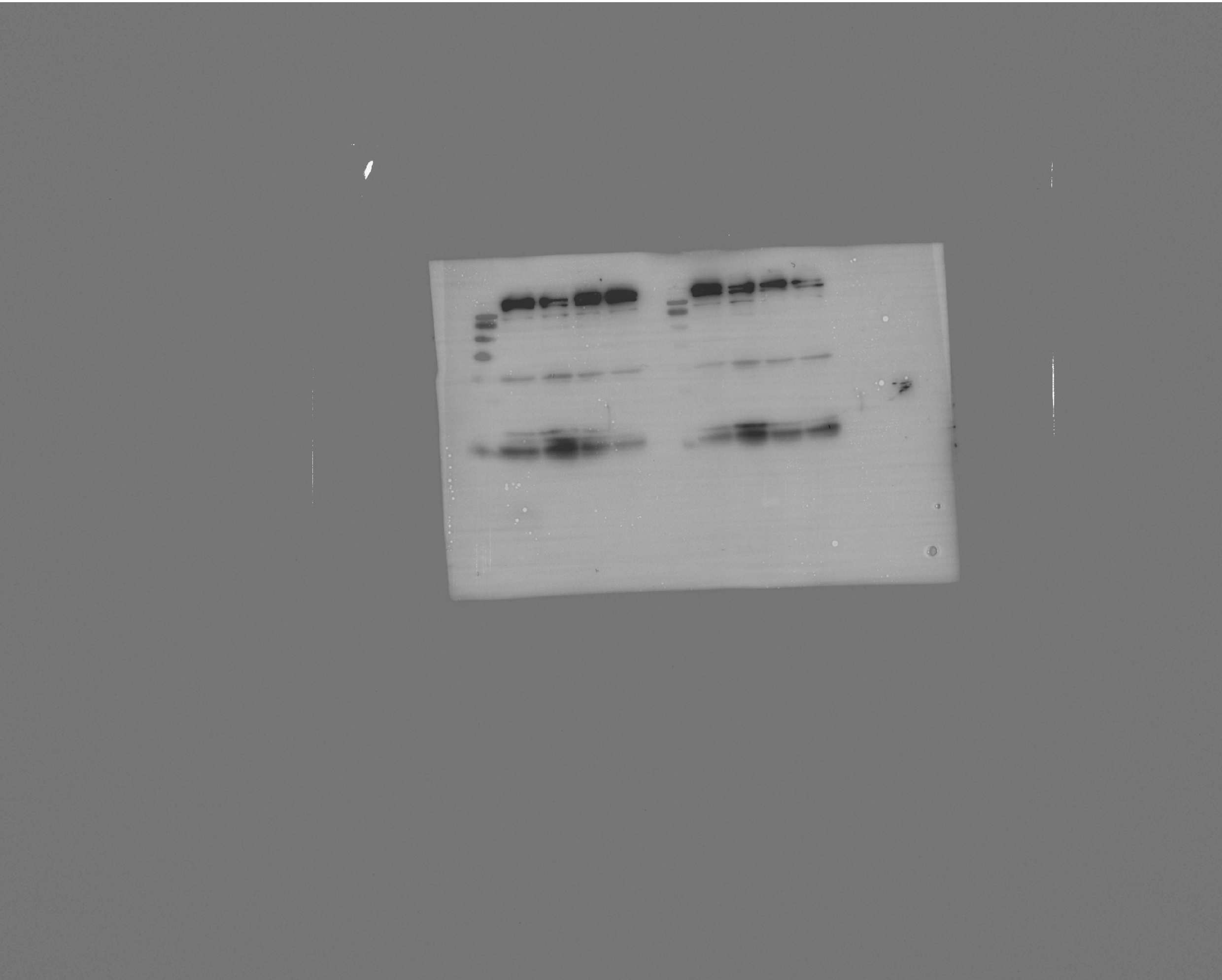

Figure_5H_pY216 GSK3B

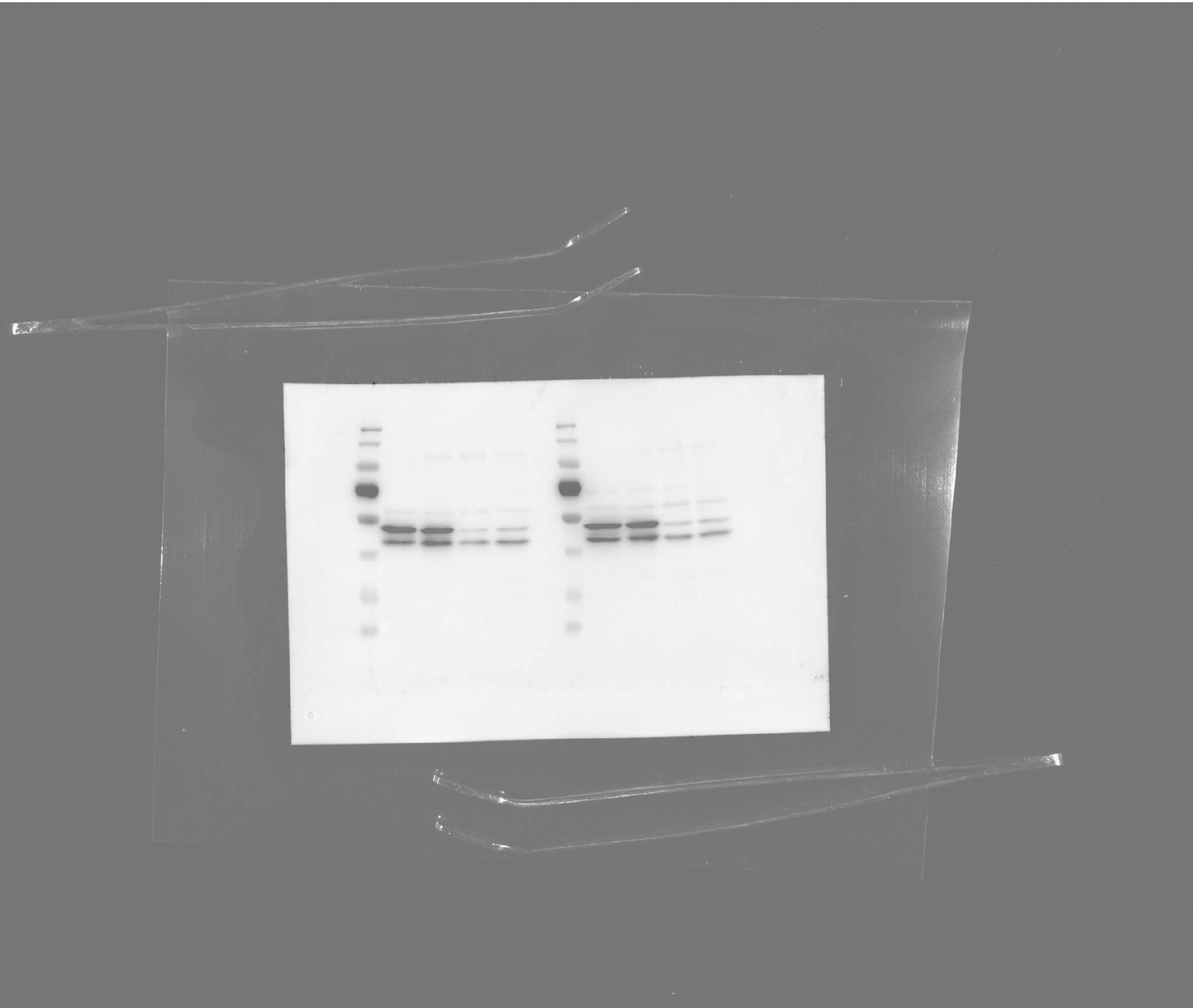

Figure_5H_total a-synuclein

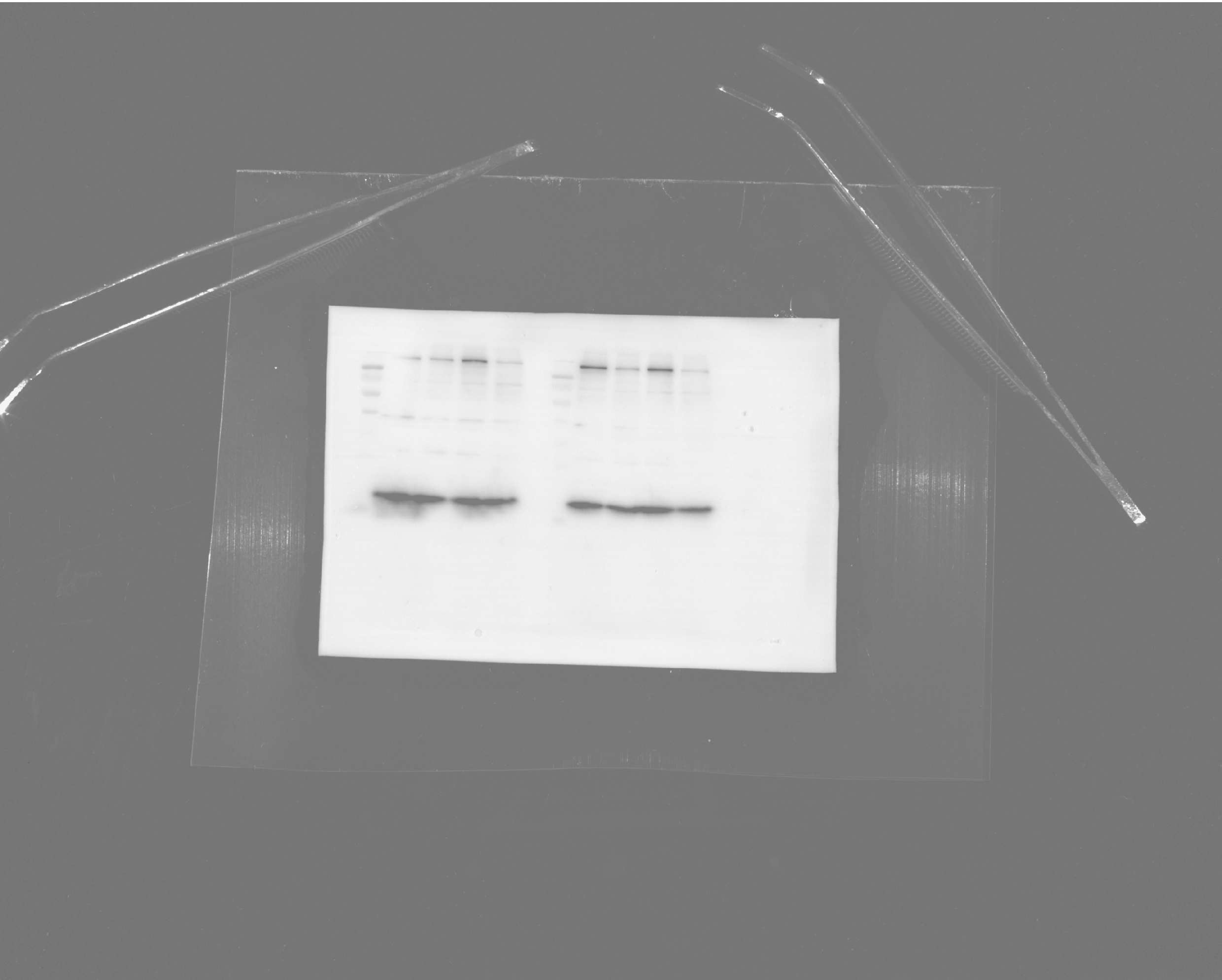

Figure_5H_total GSK3B

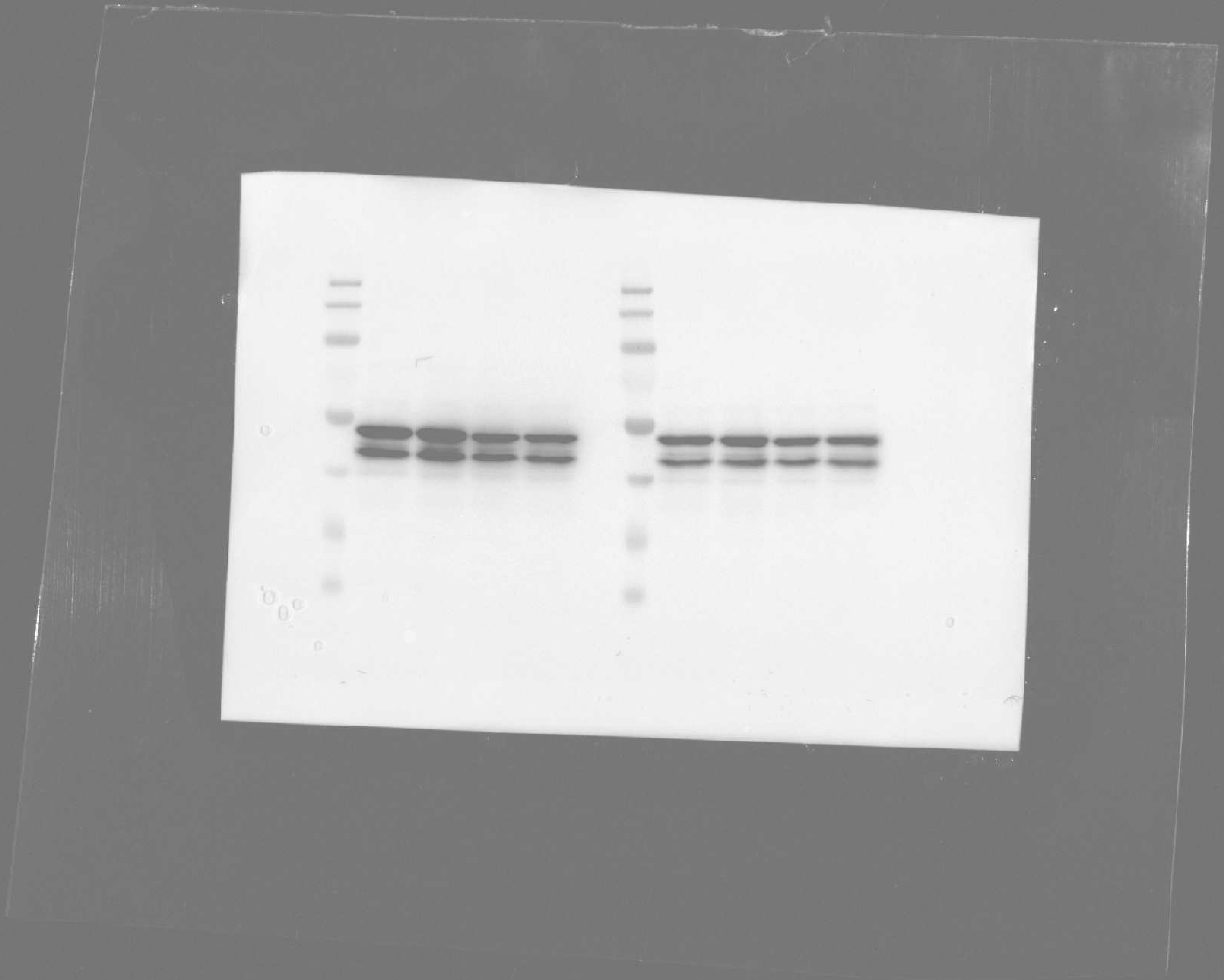

Figure_6A_beta Actin

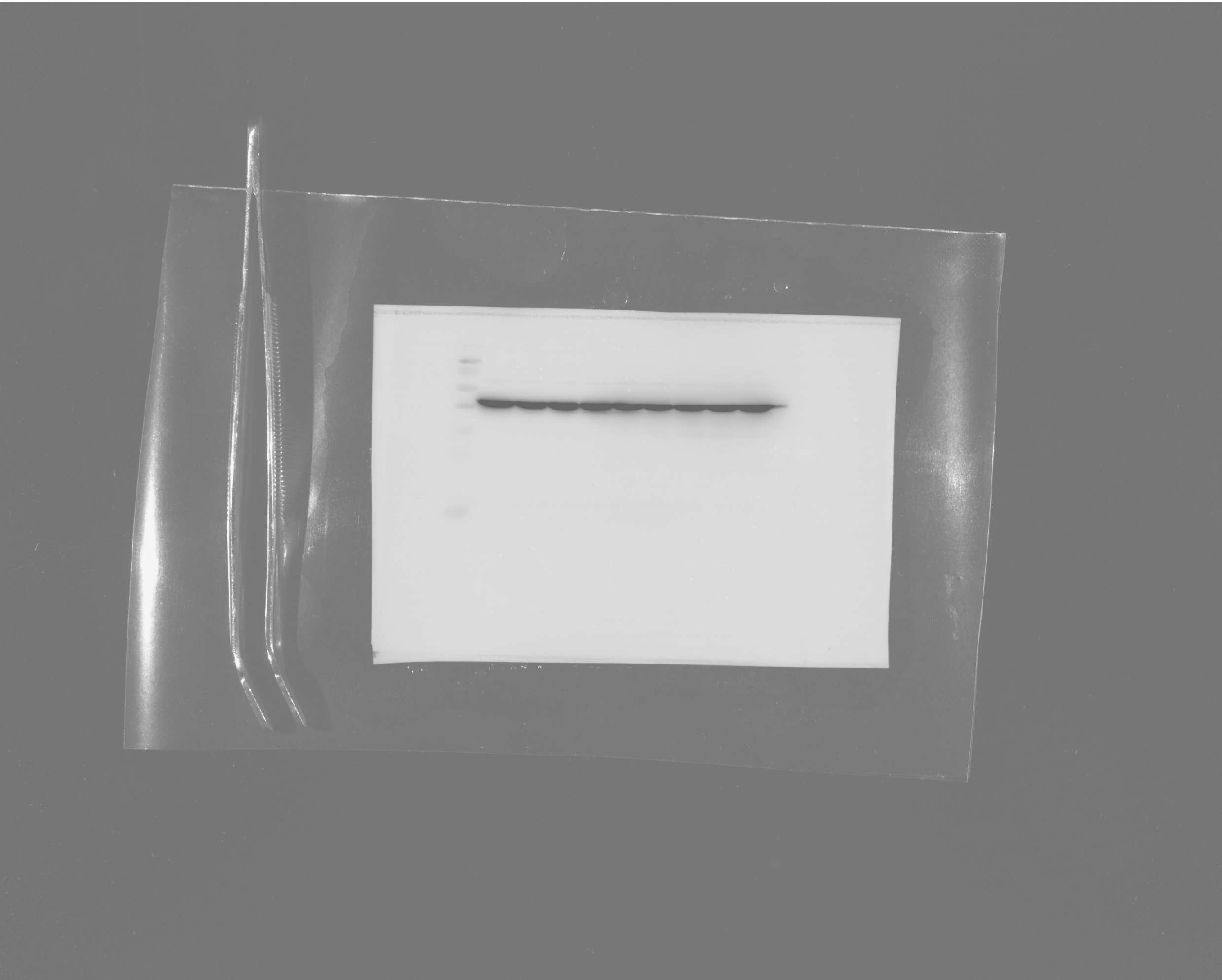

Figure_6A_pSer129 a-synuclein

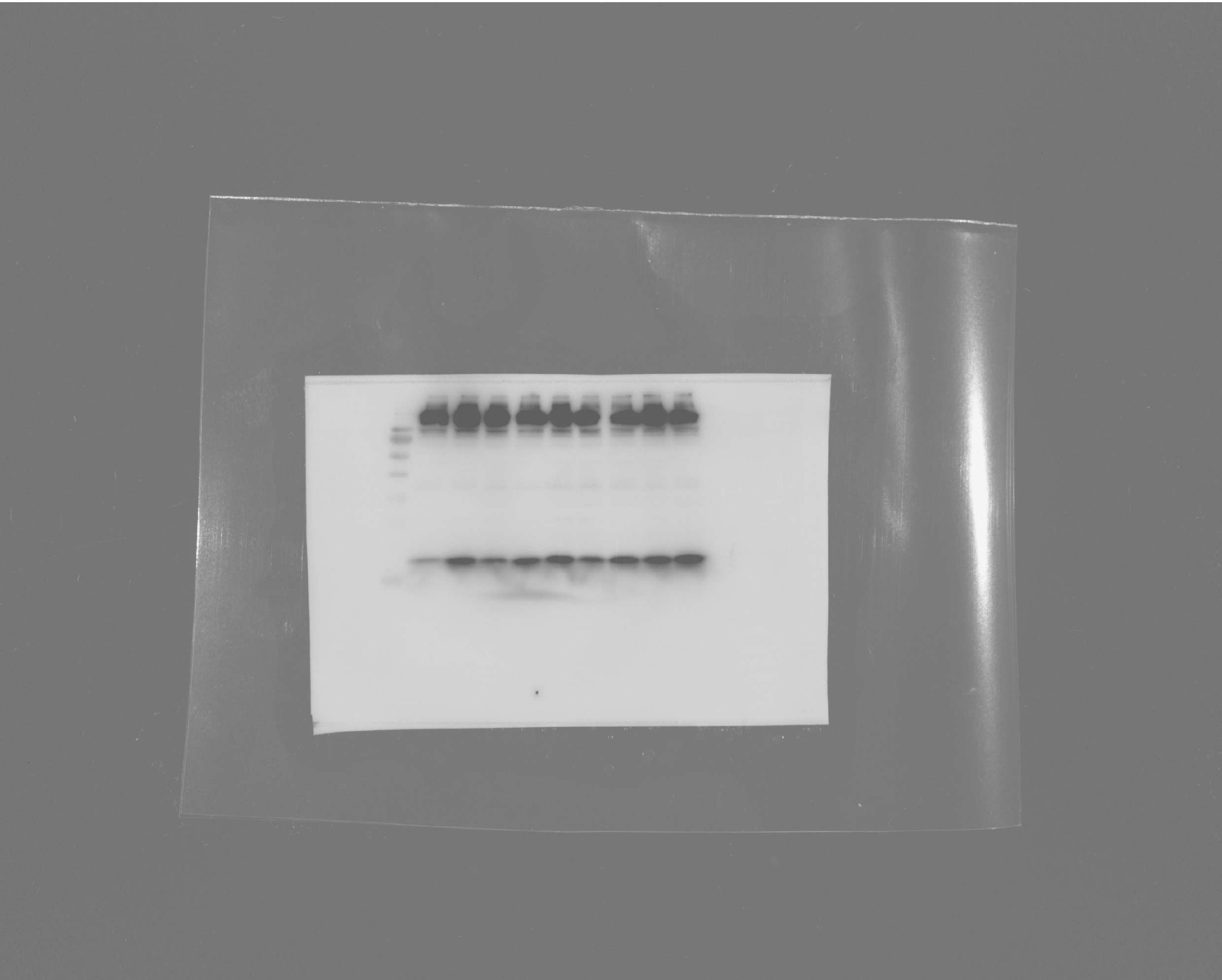

Figure_6A_Total a-synuclein

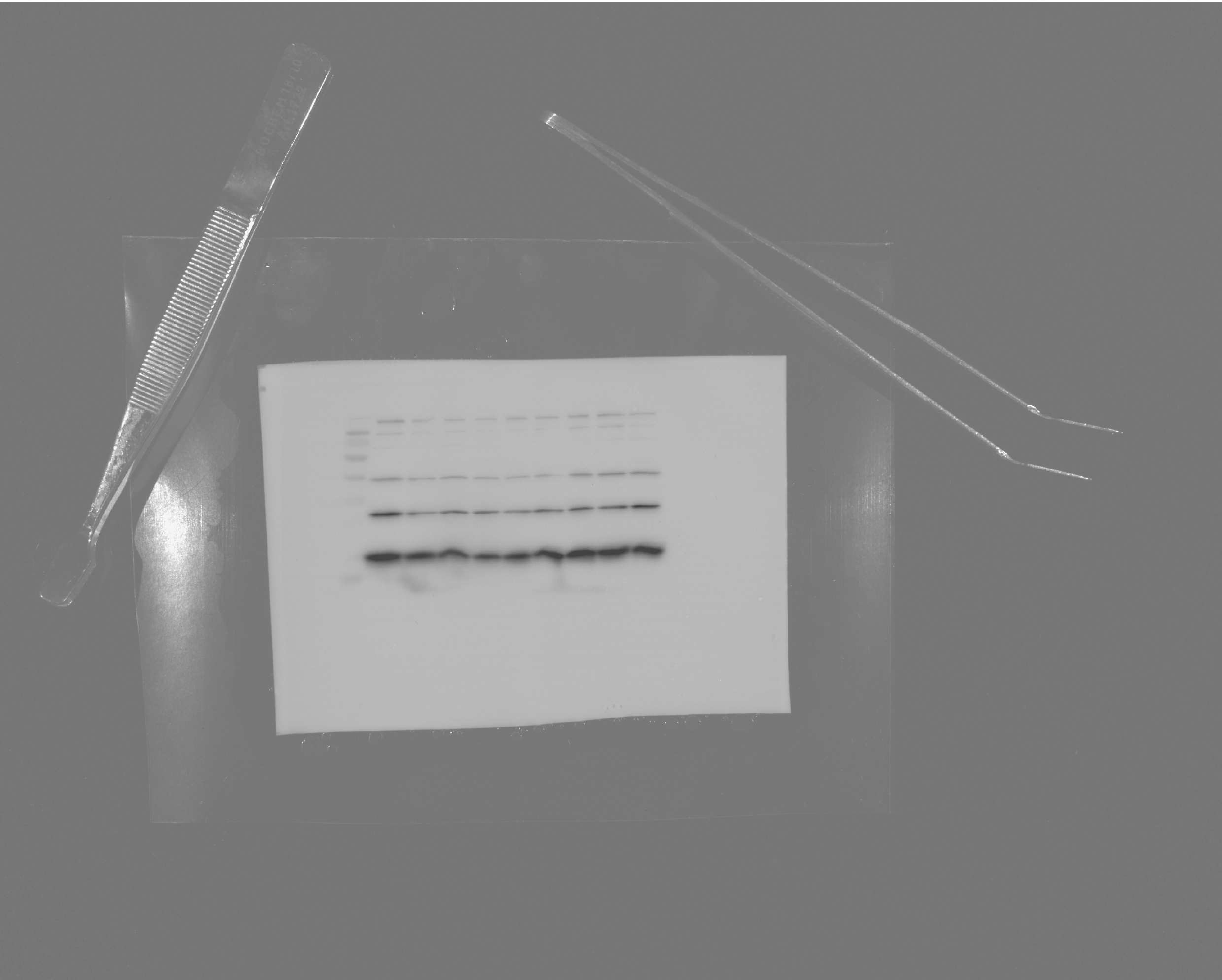

Figure_6B_beta Actin

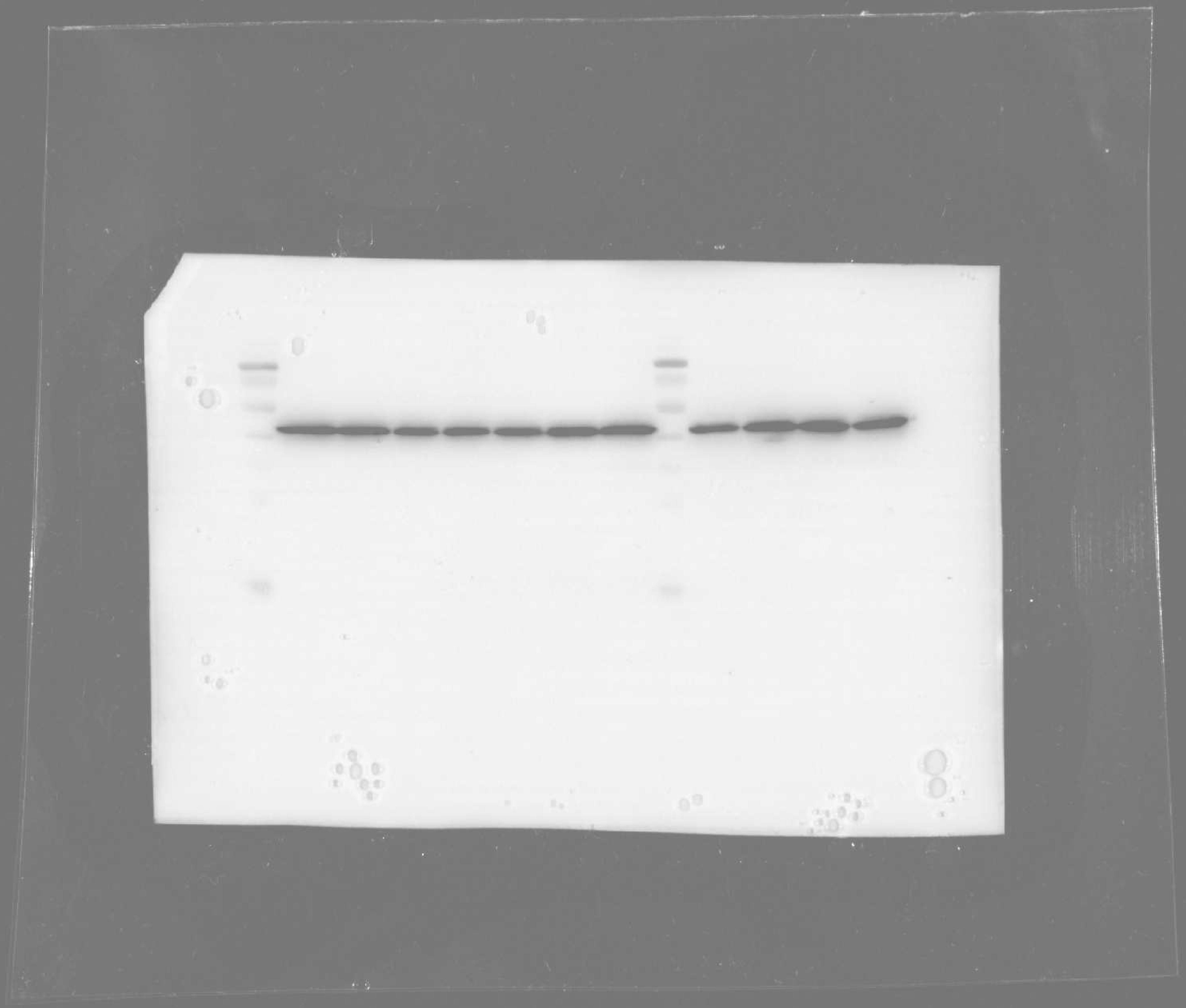

Figure_6B_pS129 a-Synuclein

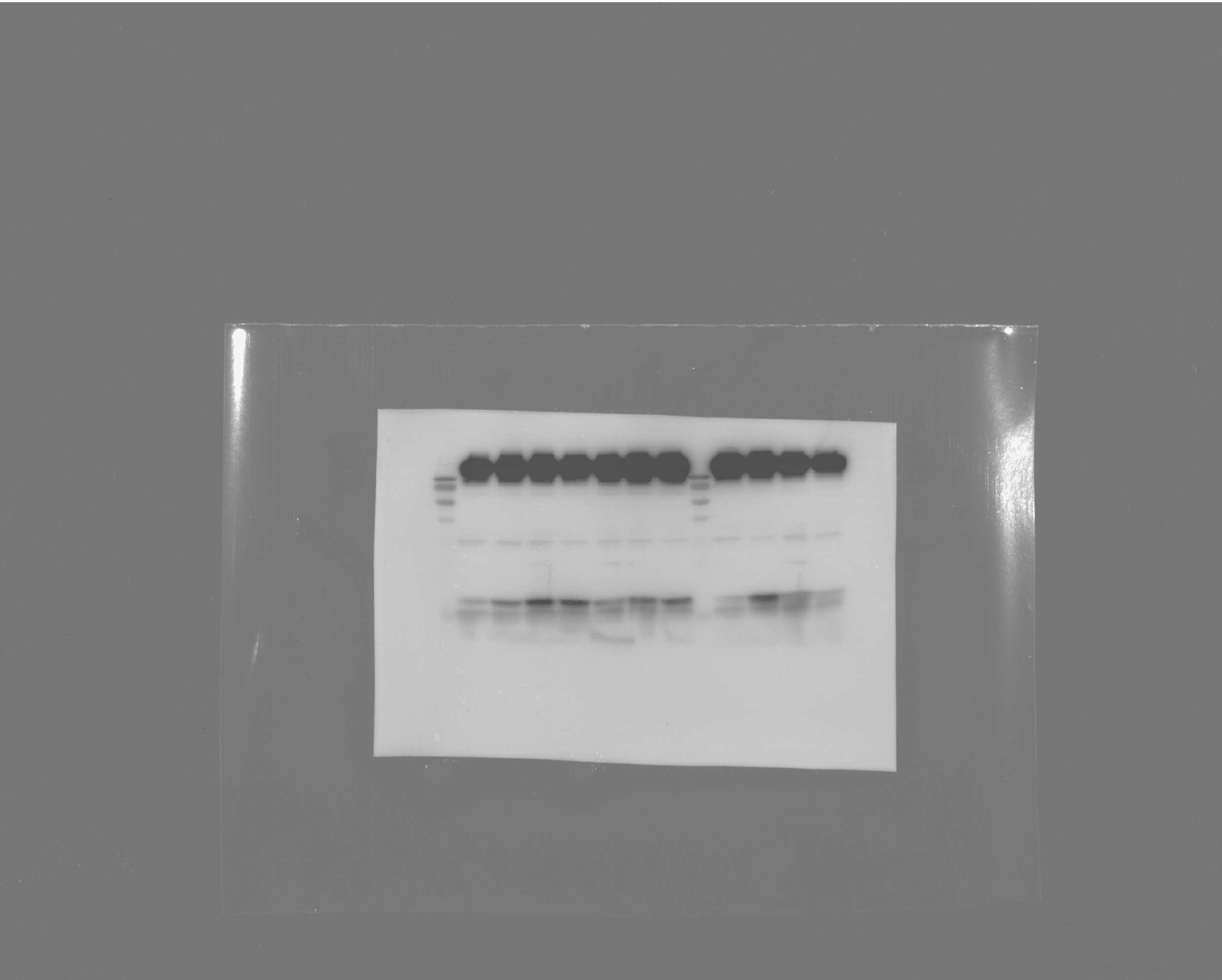

Figure_6B_pY216 GSK3B

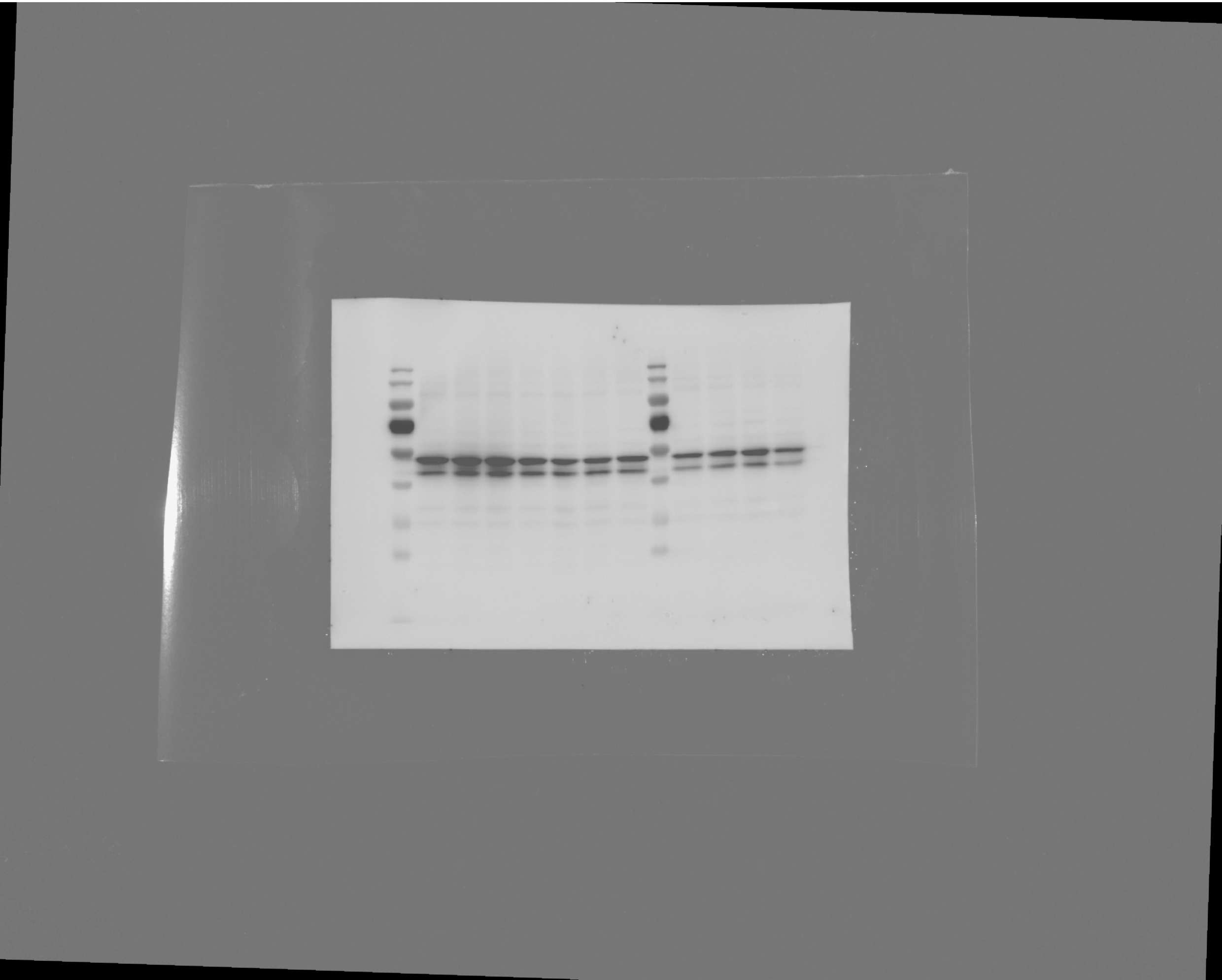

Figure_6B_pY245 cAbl

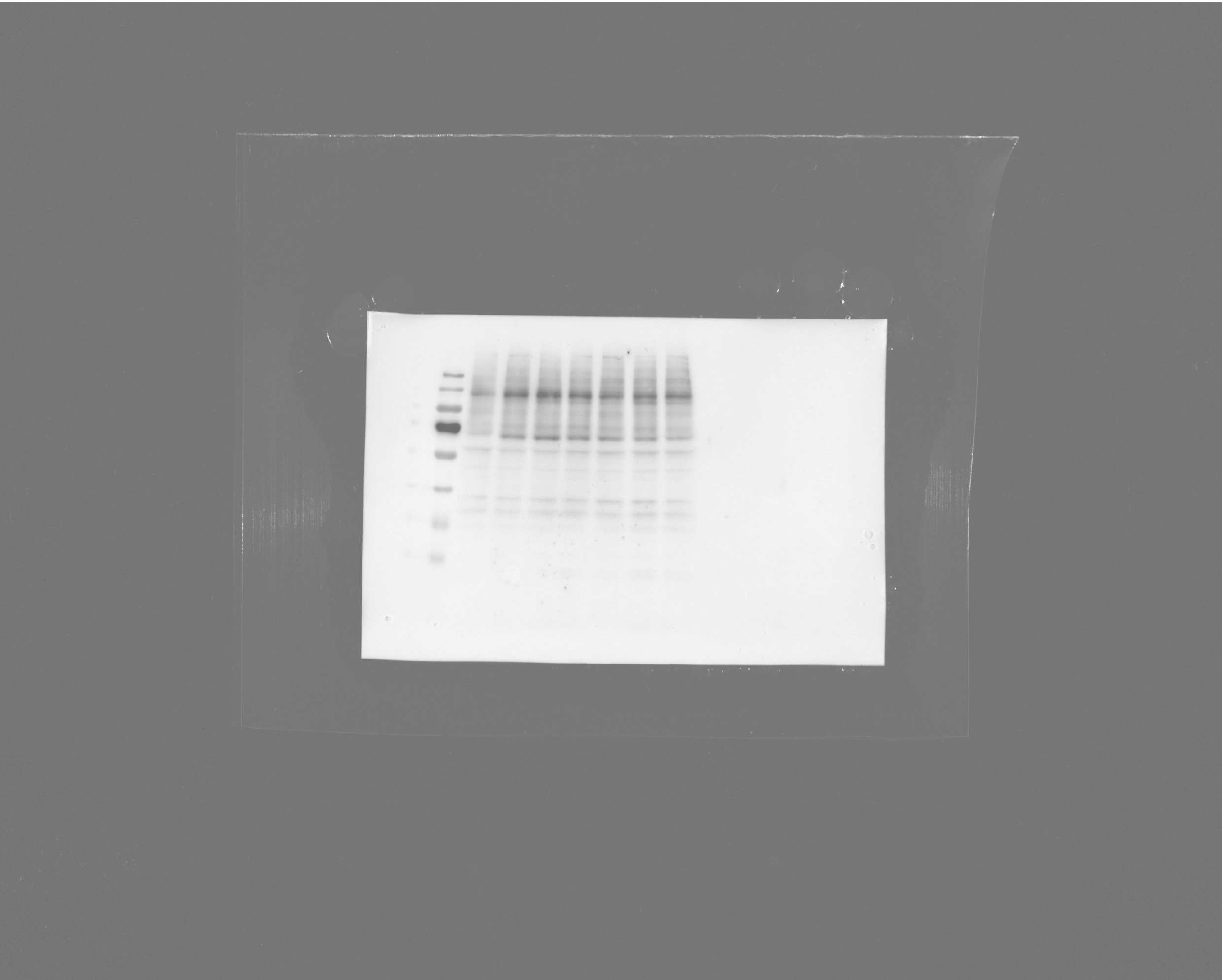

Figure_6B_pY412 cAbl

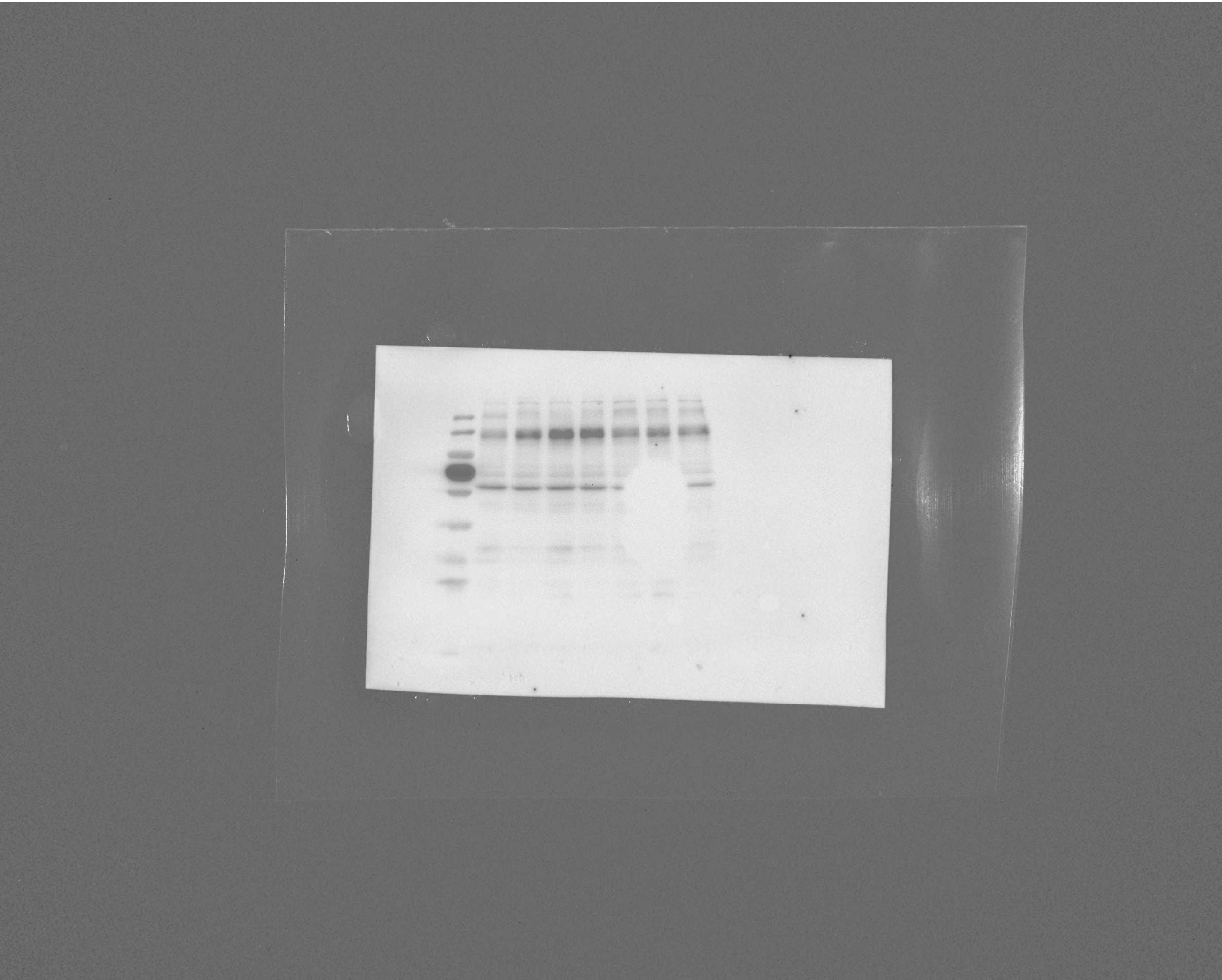

Figure_6B_total a-Synuclein

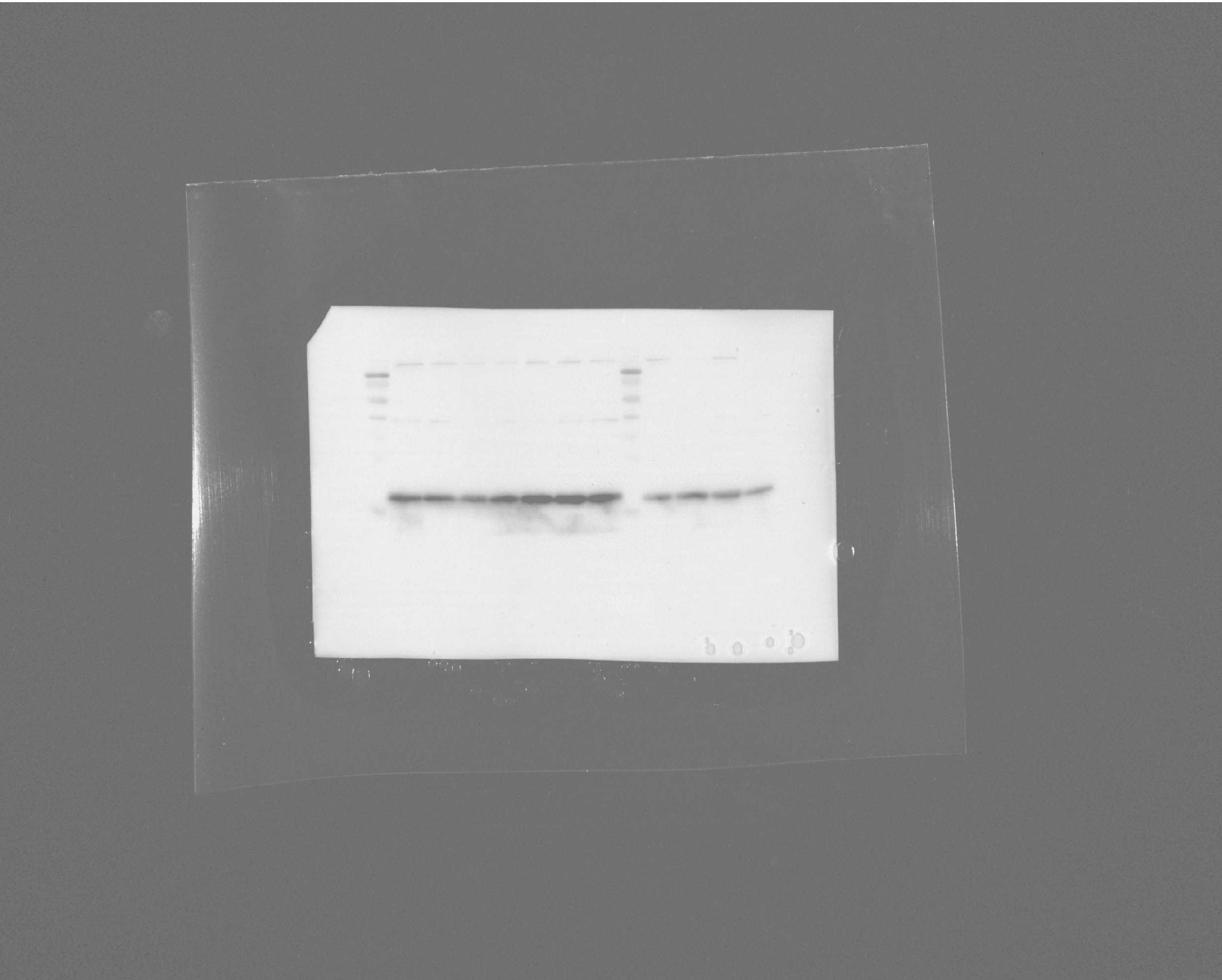

Figure_6B_total cAbl

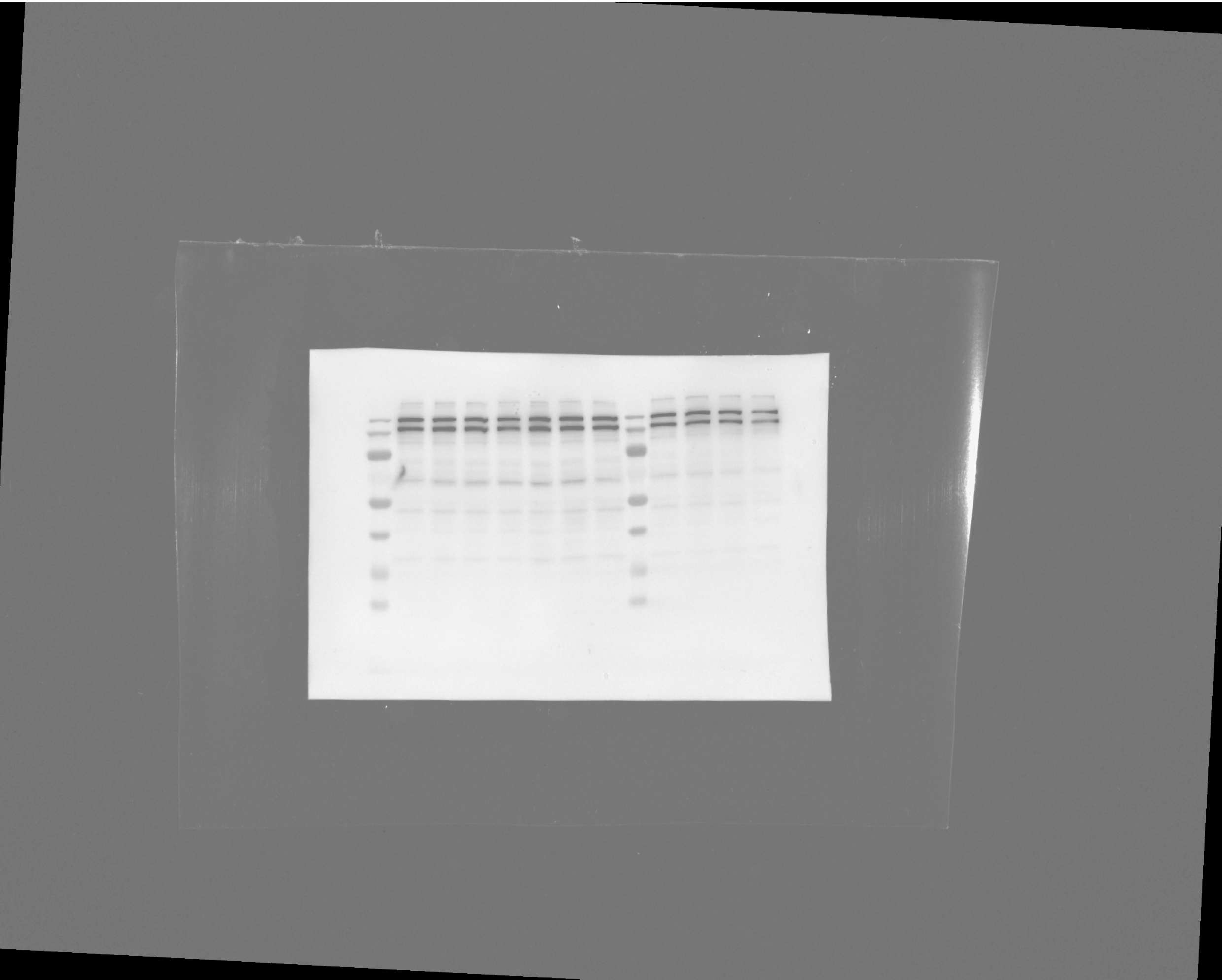

Figure_6B_total GSK3B

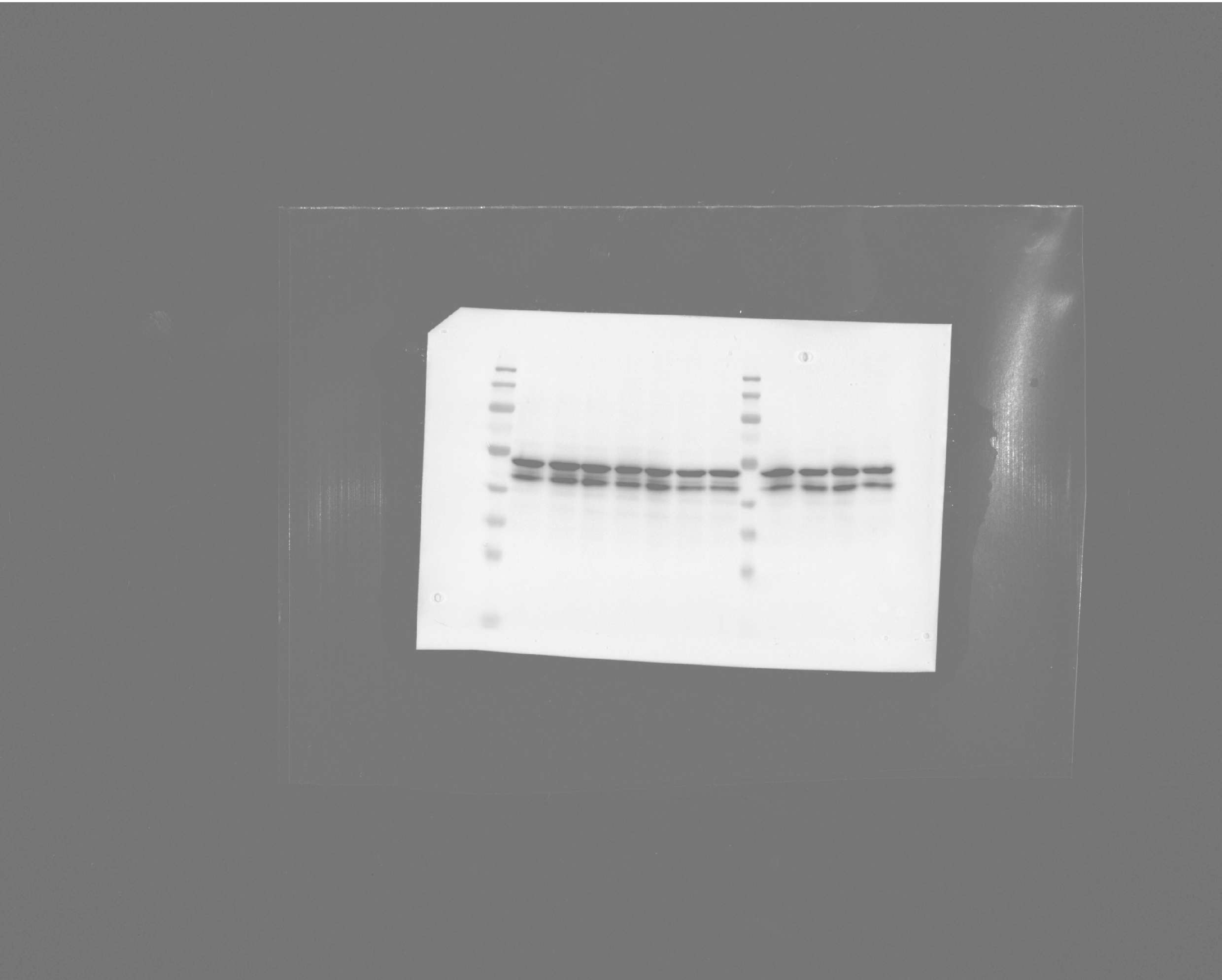

Infection and herbicide exposure implicate c-Abl kinase in α-Synuclein Ser129 phosphorylation

Marzieh Ehsani [1#*], Zeyang Sun [1,2#], Alvaro Quevedo-Olmos [1], Gesa Rösler [1], Mahdi Rasa [3,4], David Holthaus [1,5], Saskia F. Erttmann [1], and Thomas F. Meyer [1,6*]

Description of membranes in detail

Please see the original *.TIF pictures (the full membranes) in the folder.

For all the gels, pager ruller from Thermofischer cat#26616 was used.

SDS-PAGE band profile of the PageRuler Prestained Protein Ladder

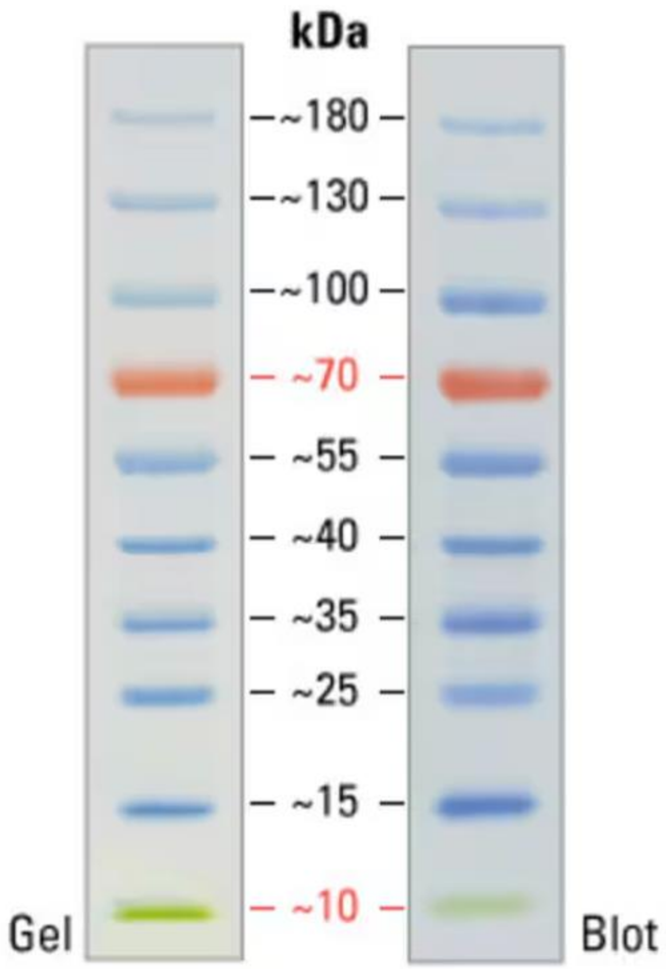

Fig. 1A

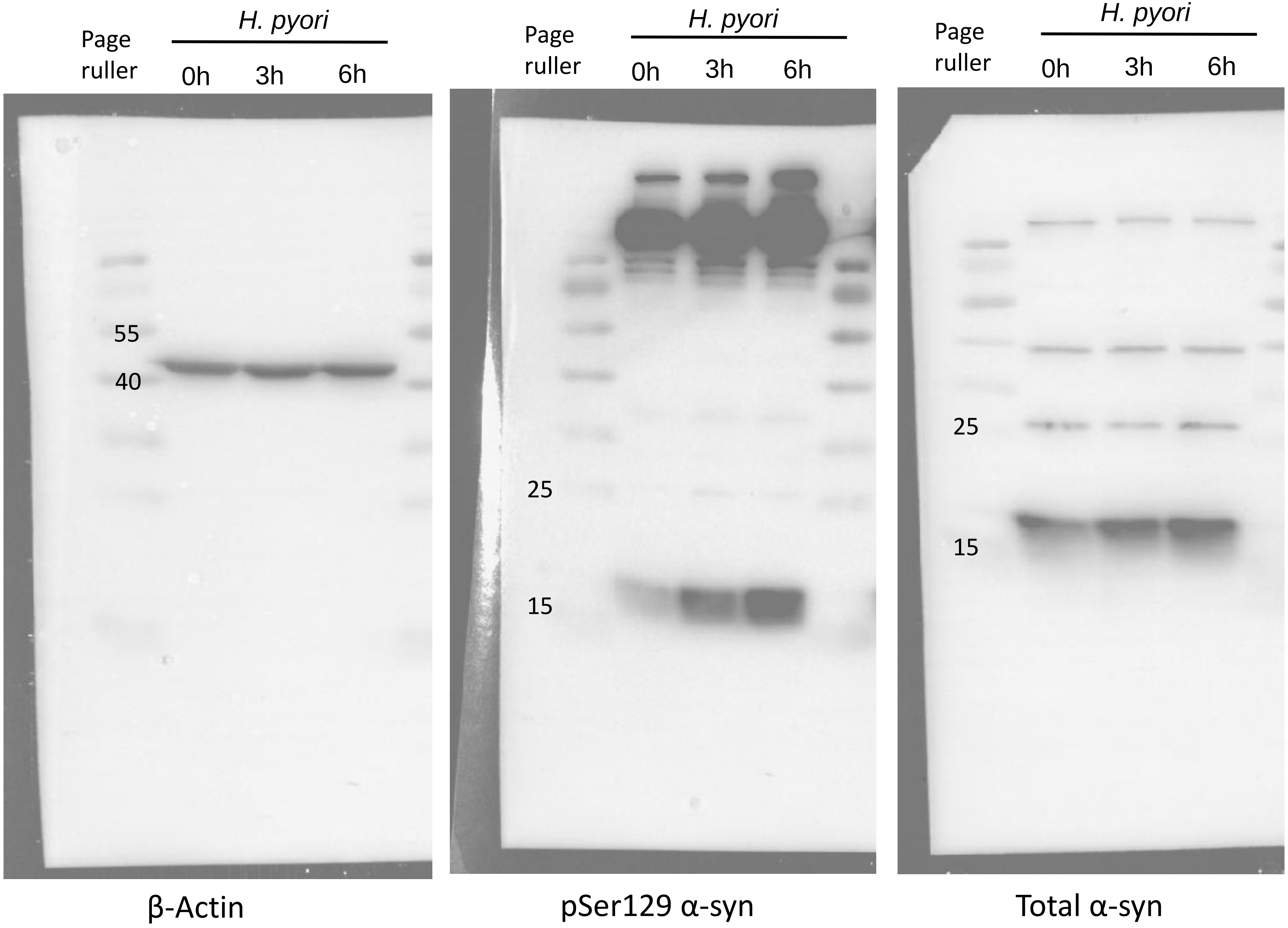

Page ruller
H. pyori
0h
3h
6h
55
40
Page ruller
H. pyori
0h
3h
6h
25
15
Page ruller
H. pyori
0h
3h
6h
25
15
β-Actin
pSer129 α-syn
Total α-syn

Fig. 1C

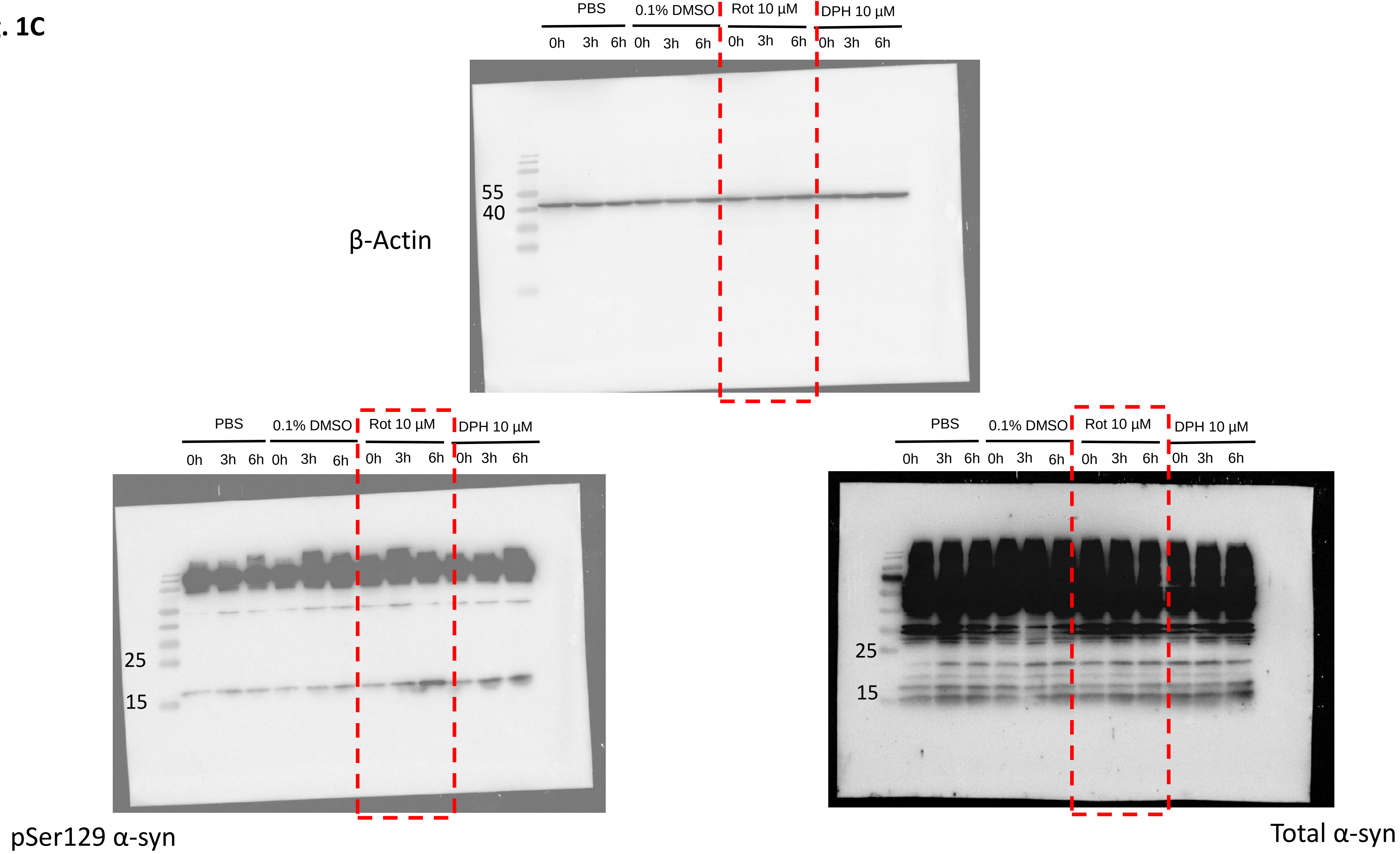

PBS
0.1% DMSO
Rot 10 µM
DPH 10 µM
0h 3h 6h 0h 3h 6h 0h 3h 6h 0h 3h 6h
55
40
β-Actin
PBS
0.1% DMSO
Rot 10 µM
DPH 10 µM
0h 3h 6h 0h 3h 6h 0h 3h 6h 0h 3h 6h
25
15
pSer129 α-syn
PBS
0.1% DMSO
Rot 10 µM
DPH 10 µM
0h 3h 6h 0h 3h 6h 0h 3h 6h 0h 3h 6h
25
15
Total α-syn

Fig. 2B

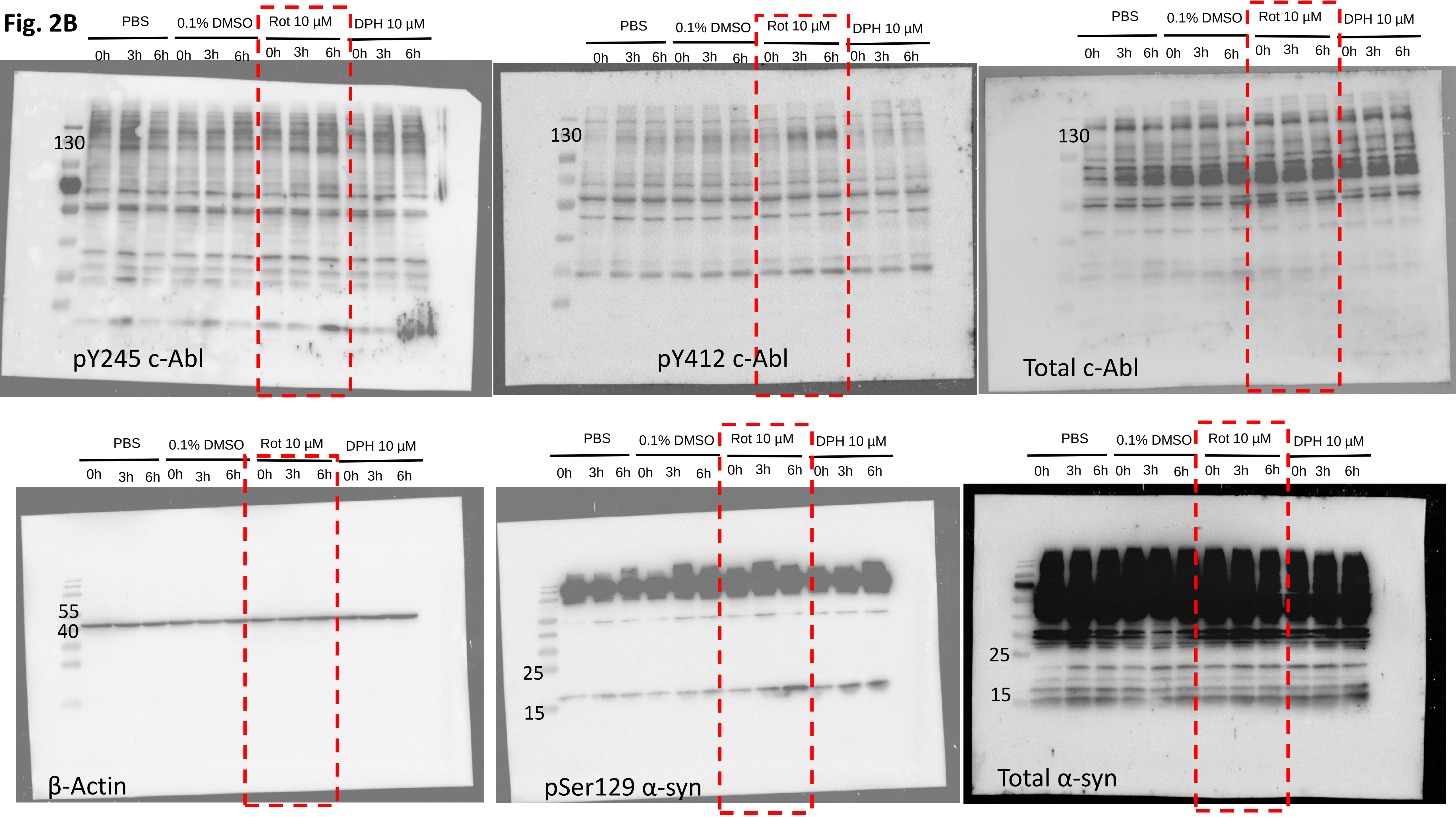
PBS
0.1% DMSO
Rot 10 μM
DPH 10 μM
0h 3h 6h 0h 3h 6h 0h 3h 6h 0h 3h 6h
130
pY245 c-Abl
pY412 c-Abl
Total c-Abl
55
40
β-Actin
25
15
pSer129 α-syn
Total α-syn

Fig. 2C

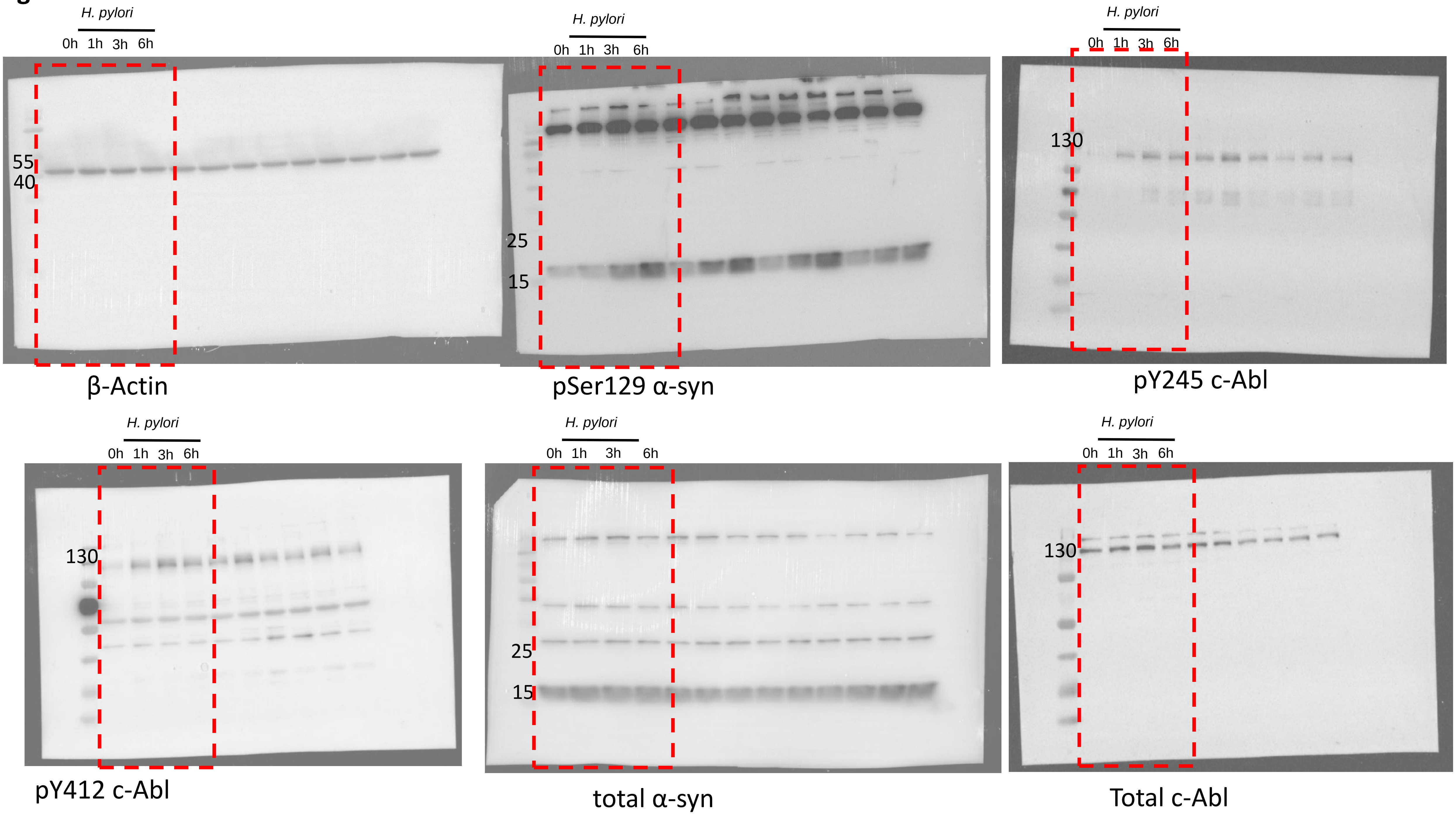
H. pylori
0h 1h 3h 6h
55
40
β-Actin
H. pylori
0h 1h 3h 6h
25
15
pSer129 α-syn
H. pylori
0h 1h 3h 6h
130
pY245 c-Abl
H. pylori
0h 1h 3h 6h
130
pY412 c-Abl
H. pylori
0h 1h 3h 6h
25
15
total α-syn
H. pylori
0h 1h 3h 6h
130
Total c-Abl

Fig. 3A

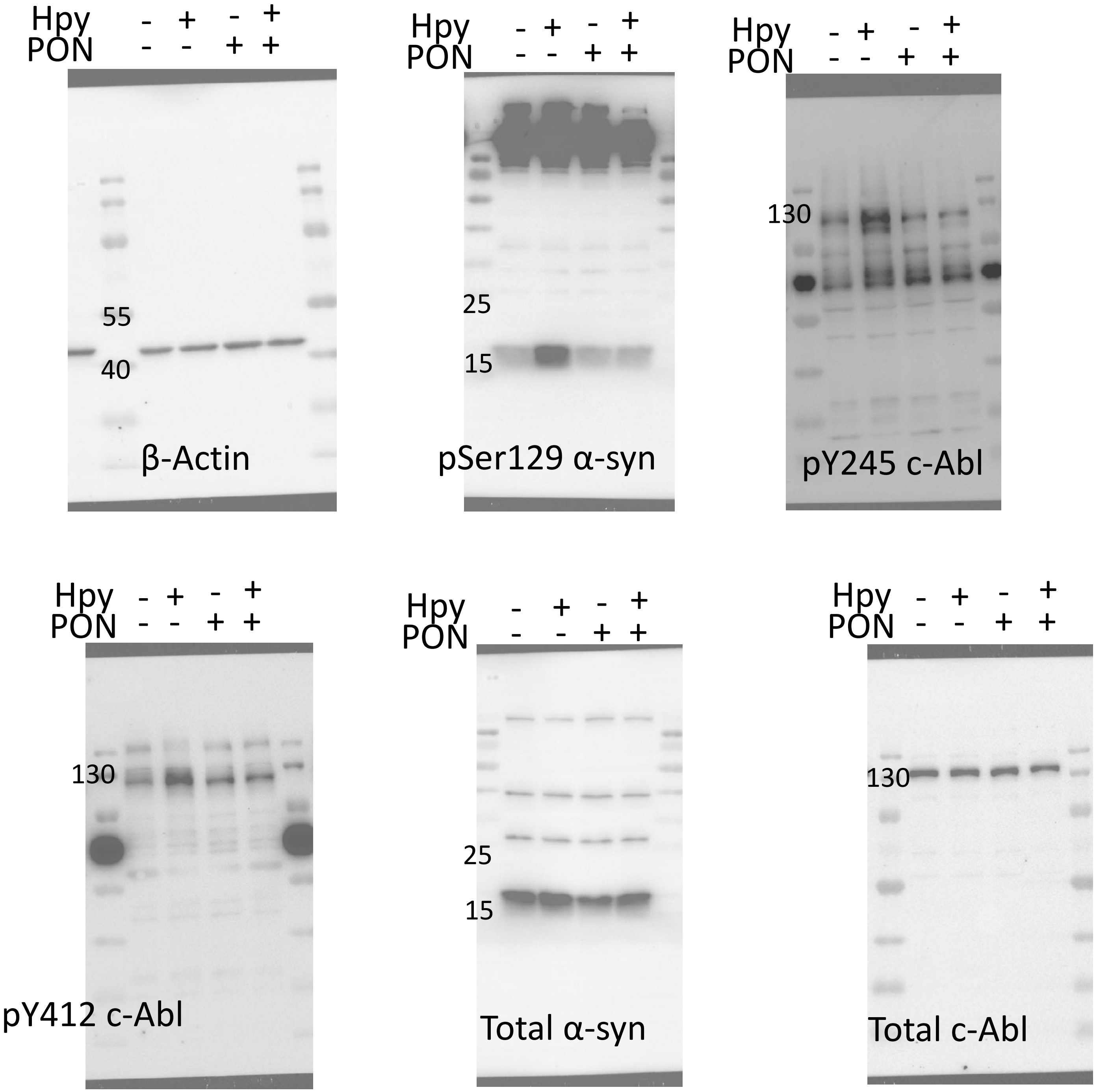

Hpy - + - +
PON - - + +
55
40
β-Actin
Hpy - + - +
PON - - + +
25
15
pSer129 α-syn
Hpy - + - +
PON - - + +
130
pY245 c-Abl
Hpy - + - +
PON - - + +
130
pY412 c-Abl
Hpy - + - +
PON - - + +
25
15
Total α-syn
Hpy - + - +
PON - - + +
130
Total c-Abl

Fig. 3B

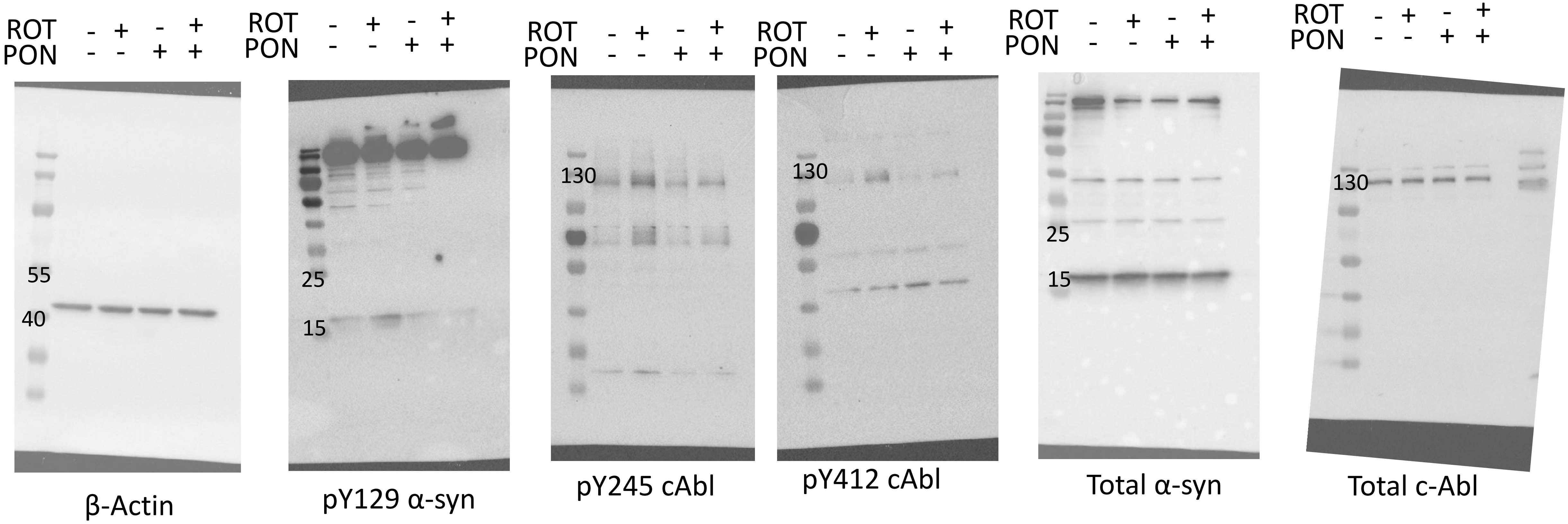
ROT - + - +
PON - - + +
55
40
β-Actin
ROT - + - +
PON - - + +
25
15
pY129 α-syn
ROT - + - +
PON - - + +
130
pY245 cAbl
ROT - + - +
PON - - + +
130
pY412 cAbl
ROT - + - +
PON - - + +
25
15
Total α-syn
ROT - + - +
PON - - + +
130
Total c-Abl

Fig. 5C

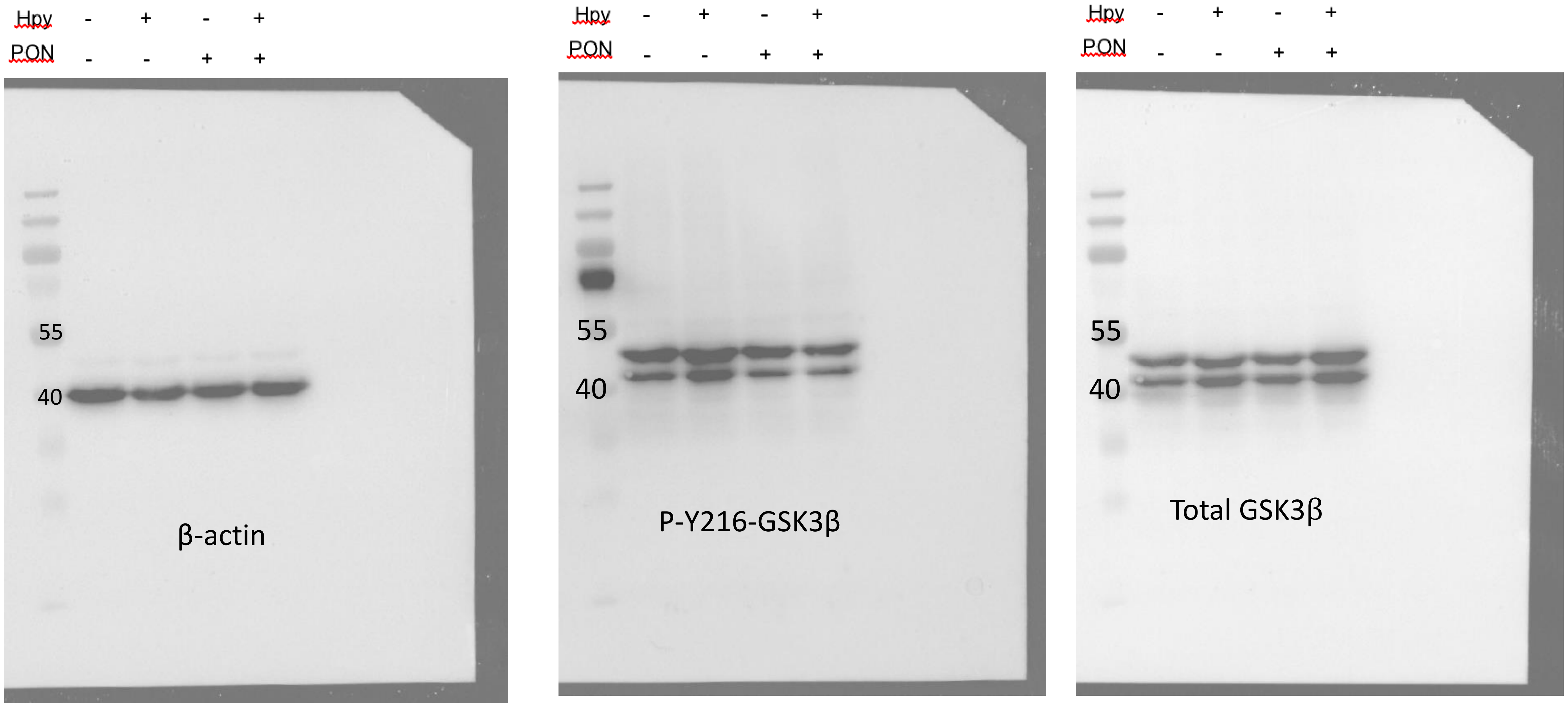

Hpy - + - +
PON - - + +
55
40
β-actin
Hpy - + - +
PON - - + +
55
40
P-Y216-GSK3β
Hpy - + - +
PON - - + +
55
40
Total GSK3β

Fig. 5D

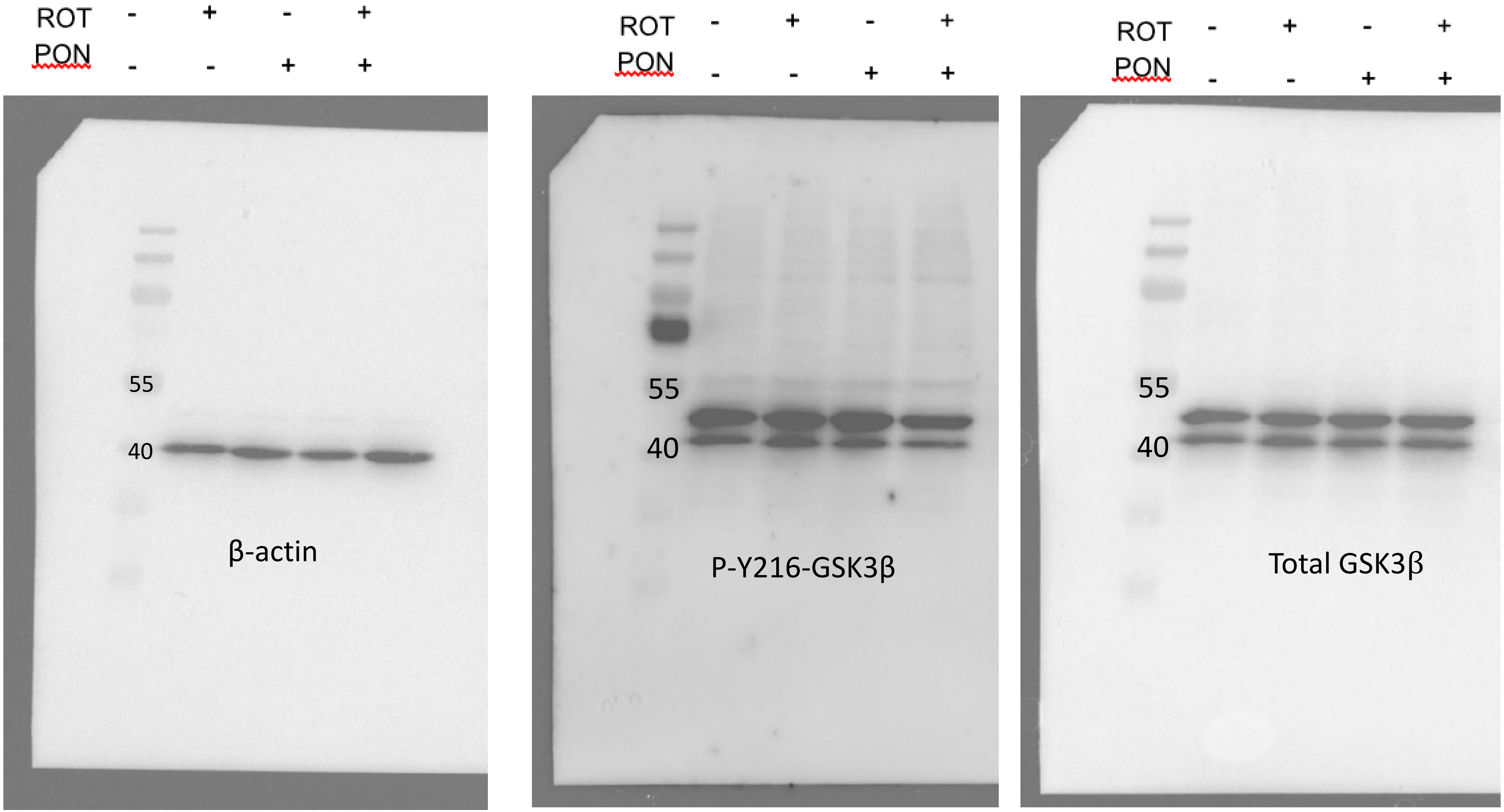
ROT - + - +
PON - - + +
55
40
β-actin
ROT - + - +
PON - - + +
55
40
P-Y216-GSK3β
ROT - + - +
PON - - + +
55
40
Total GSK3β

Fig. 5E

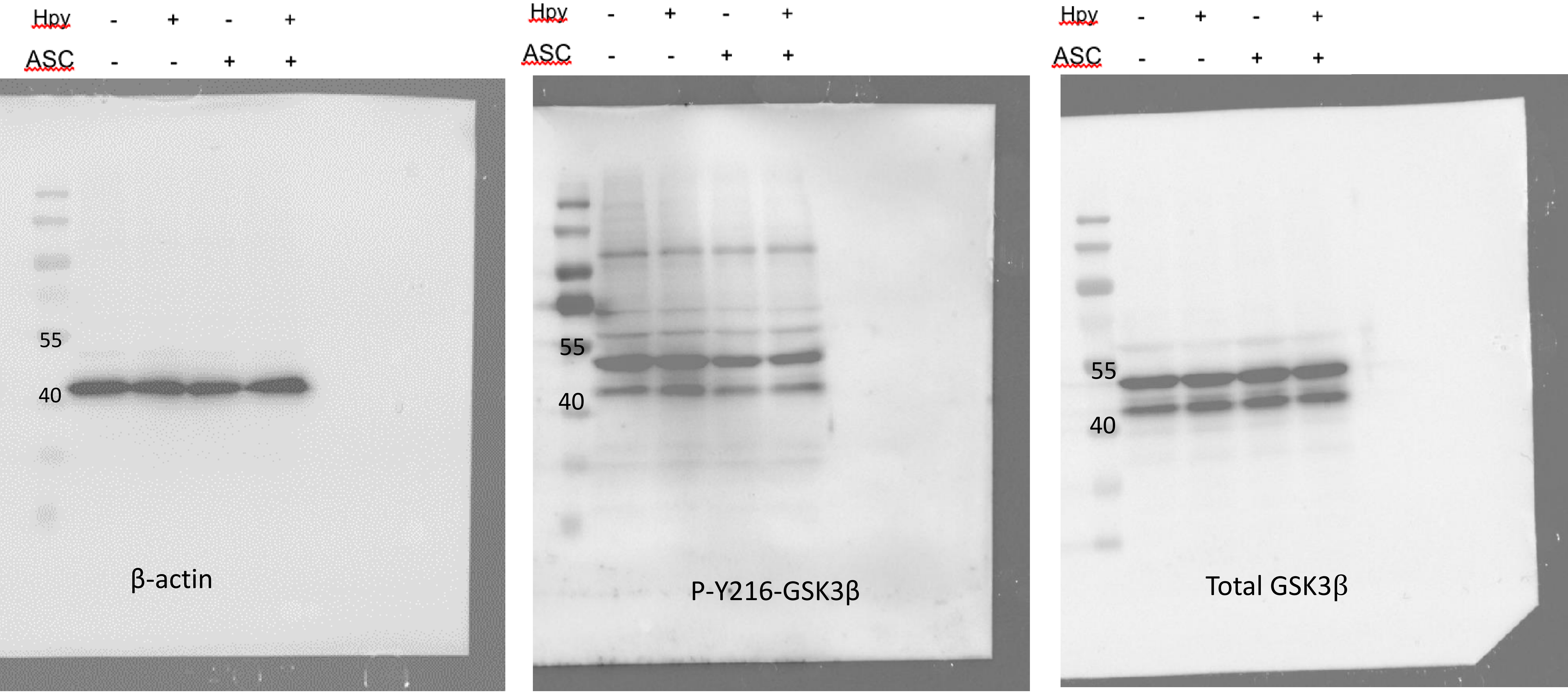
Hpy - + - +
ASC - - + +
55
40
β-actin
Hpy - + - +
ASC - - + +
55
40
P-Y216-GSK3β
Hpy - + - +
ASC - - + +
55
40
Total GSK3β

Fig. 5F

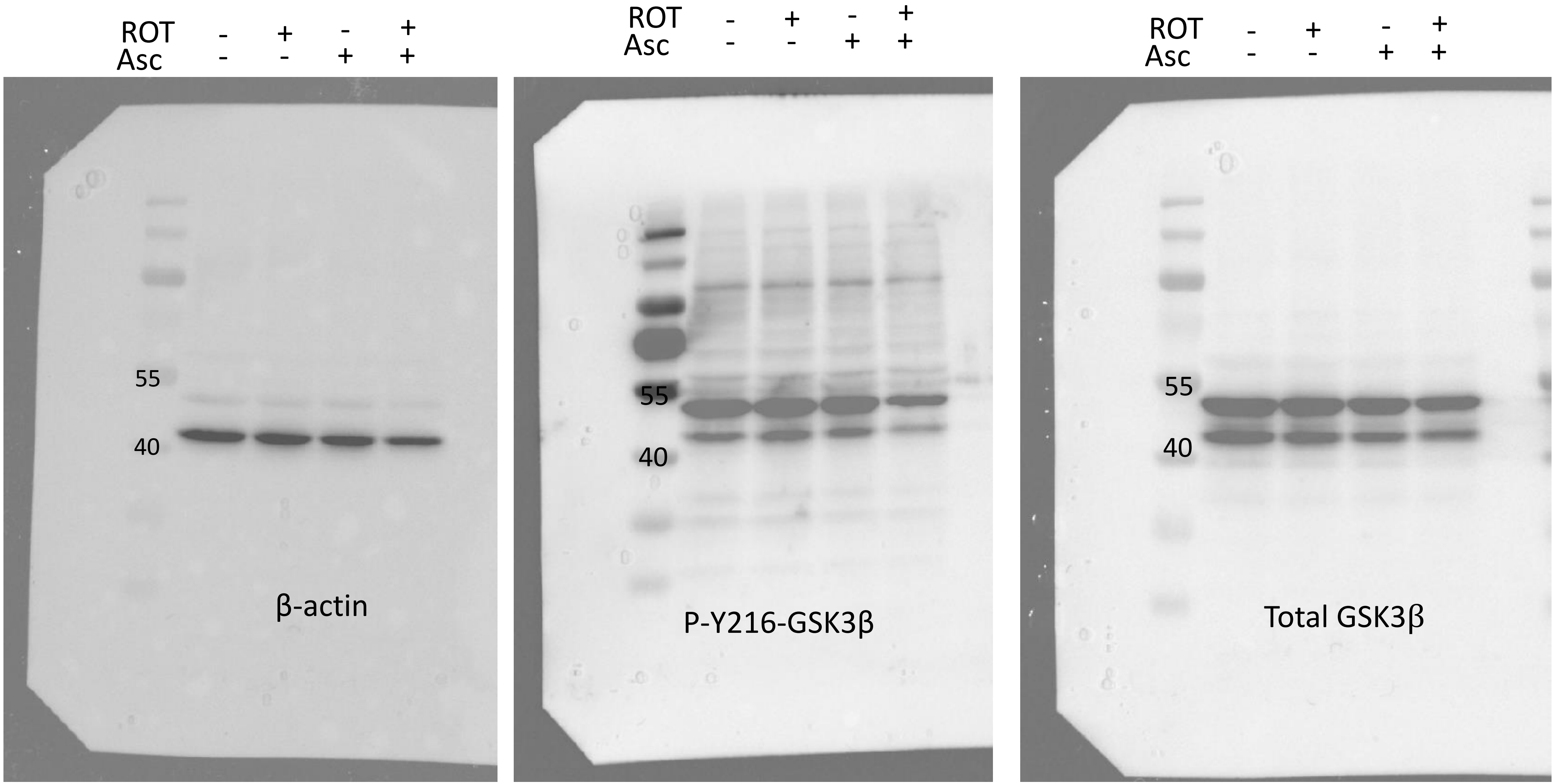

ROT - + - +
Asc - - + +
55
40
β-actin
ROT - + - +
Asc - - + +
55
40
P-Y216-GSK3β
ROT - + - +
Asc - - + +
55
40
Total GSK3β

Fig. 5G

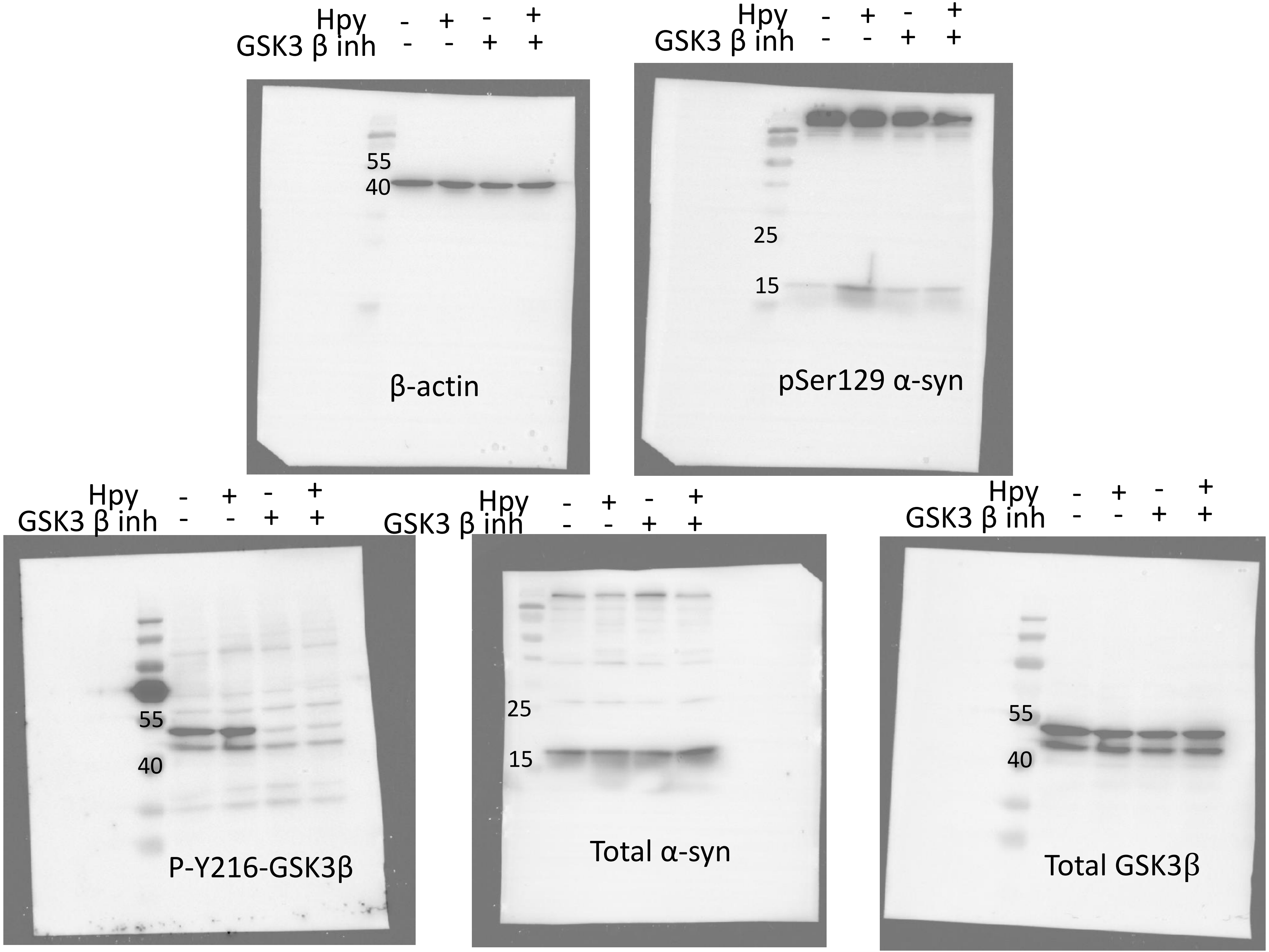
Hpy - + - +
GSK3 β inh - - + +
55
40
β-actin
Hpy - + - +
GSK3 β inh - - + +
25
15
pSer129 α-syn
Hpy - + - +
GSK3 β inh - - + +
55
40
P-Y216-GSK3β
Hpy - + - +
GSK3 β inh - - + +
25
15
Total α-syn
Hpy - + - +
GSK3 β inh - - + +
55
40
Total GSK3β

Fig. 5H

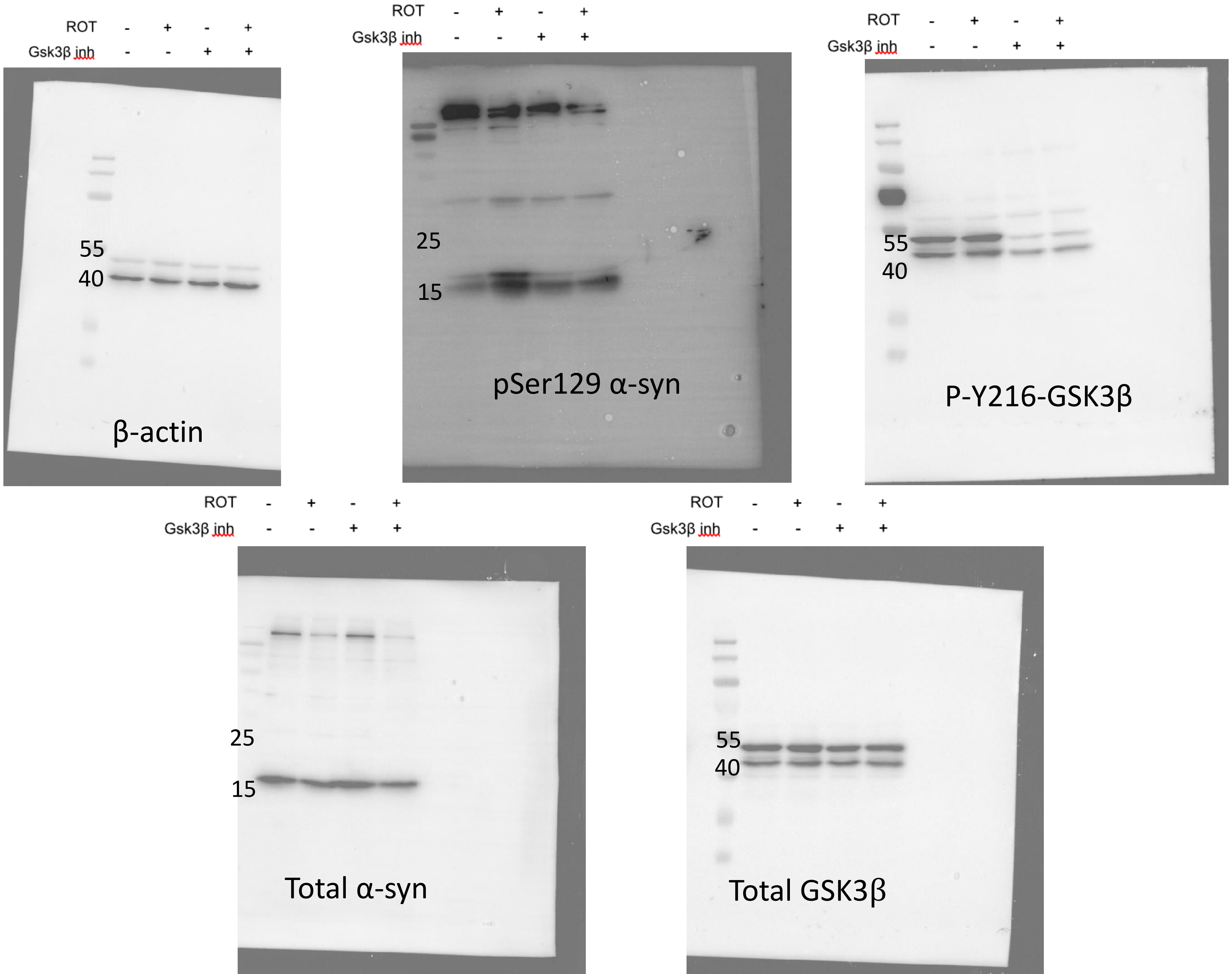
ROT - + - +
Gsk3β inh - - + +
55
40
β-actin
ROT - + - +
Gsk3β inh - - + +
25
15
pSer129 α-syn
ROT - + - +
Gsk3β inh - - + +
55
40
P-Y216-GSK3β
ROT - + - +
Gsk3β inh - - + +
25
15
Total α-syn
ROT - + - +
Gsk3β inh - - + +
55
40
Total GSK3β

Fig. 6A

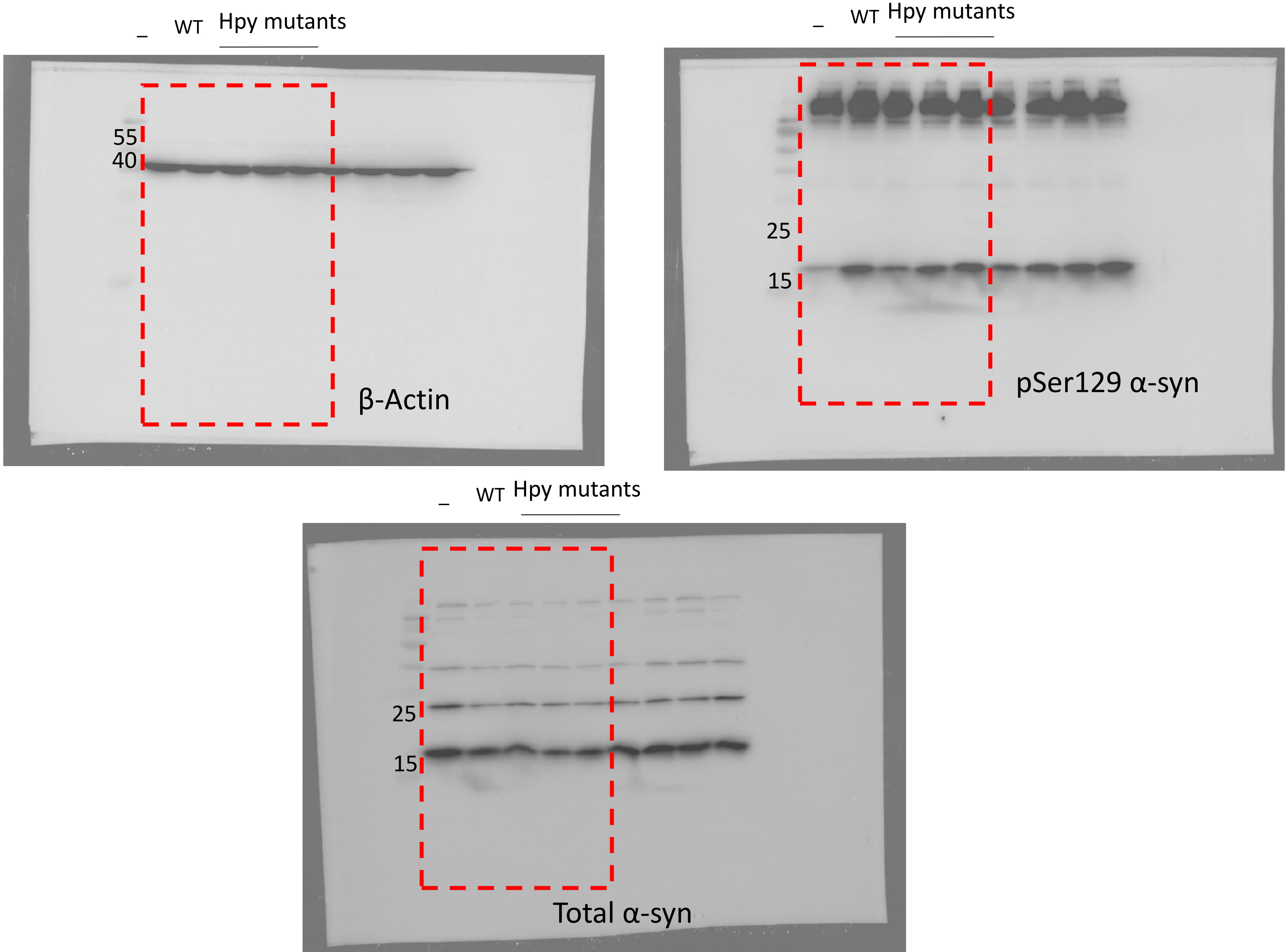

_
WT
Hpy mutants
55
40
β-Actin
_
WT
Hpy mutants
25
15
pSer129 α-syn
_
WT
Hpy mutants
25
15
Total α-syn

Fig. 6B

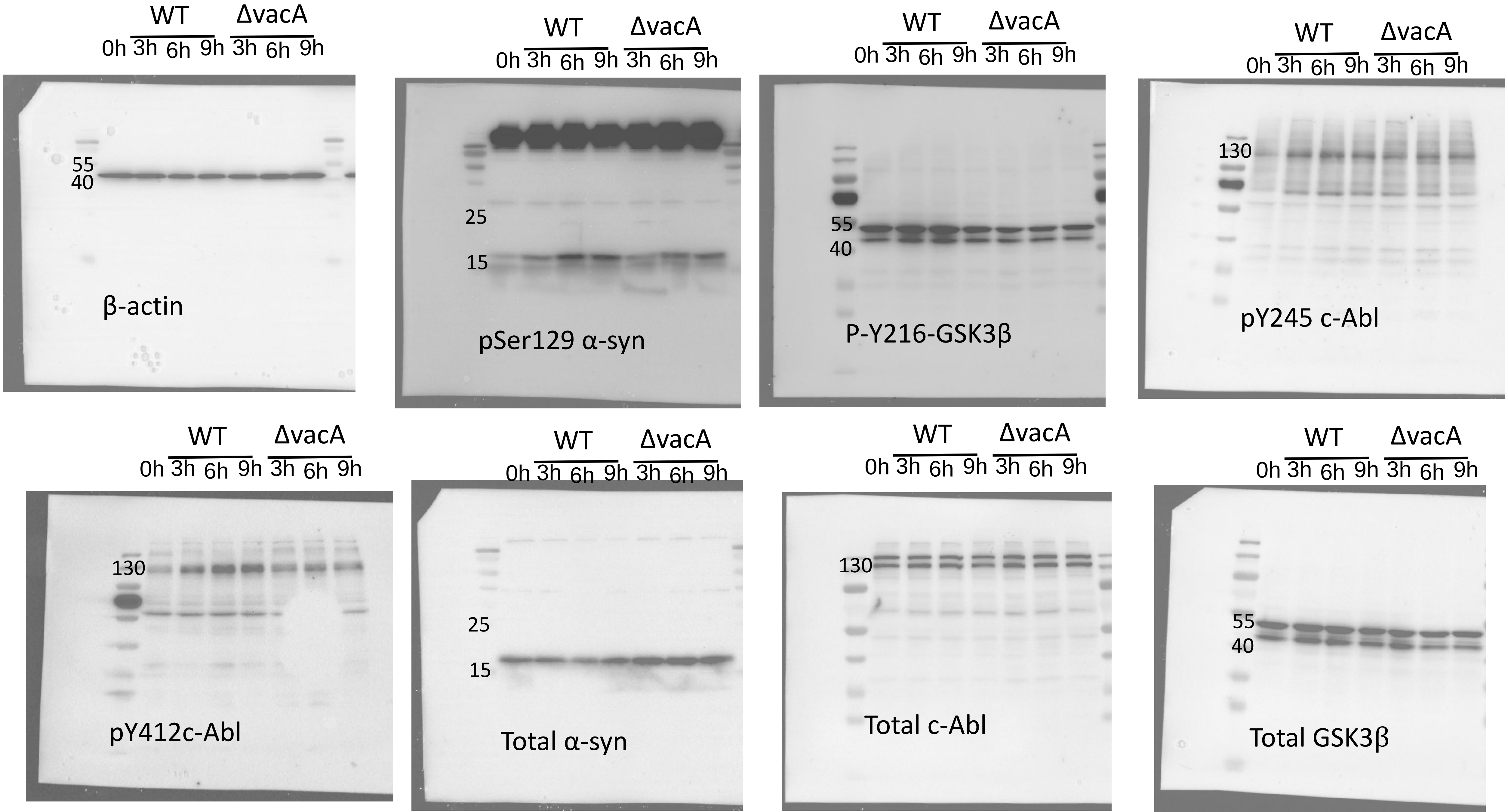
WT
ΔvacA
0h 3h 6h 9h 3h 6h 9h
55
40
β-actin
25
15
pSer129 α-syn
P-Y216-GSK3β
130
pY245 c-Abl
pY412c-Abl
Total α-syn
Total c-Abl
Total GSK3β

- **Thank you very much for your time!**
