# Supplementary material for: Infection and herbicide exposure implicate c-Abl kinase in α-Synuclein Ser129 phosphorylation
Source: Cell Commun Signal. 2025 Sep 23;23:396. doi: 10.1186/s12964-025-02399-2 (PMC12455823; doi:10.1186/s12964-025-02399-2)
Supplement: Supplementary file 2 — Supplementary Material 2: Additional file 2. (A) SH-SY5Y cells were treated with rotenone (ROT) or infected with H. pylori (Hpy) at moi100 for 6 h and Western blotting was performed on the extracted total protein. Some markers for mitochondrial dysfunction such as mitofusin-2 (MFN-2), TOM20 and parkin (PARKN) were evaluated and the intensity of the protein bands were quantified (n=2 replicate). (B-F) SH-SY5Y cells were treated with rotenone (ROT) or (C) infected with H. pylori (Hpy) and total RNA was extracted after 4h. Differentially expressed genes were identified using the DESeq2 R package (v 1.40.2). Gene Set Enrichment Analysis (GSEA) was performed on the gene list ranked by log2 Fold Changes using the fgsea (v 1.26.0) implementation of the algorithm implemented in the clusterProfiler (v 4.8.3) R package. The displayed pathways were filtered based on significance, with an adjusted p-value threshold of <0.05. (B) and (C) represent enrichment of inflammatory pathways. (D) and (E) show activation of cell kinases including tyrosine and serine-threonine kinases. The data represents as n=3 replicates. (F) The identifier of each regulated Wikipathway is represented in the table [file 12964_2025_2399_MOESM2_ESM.pdf]

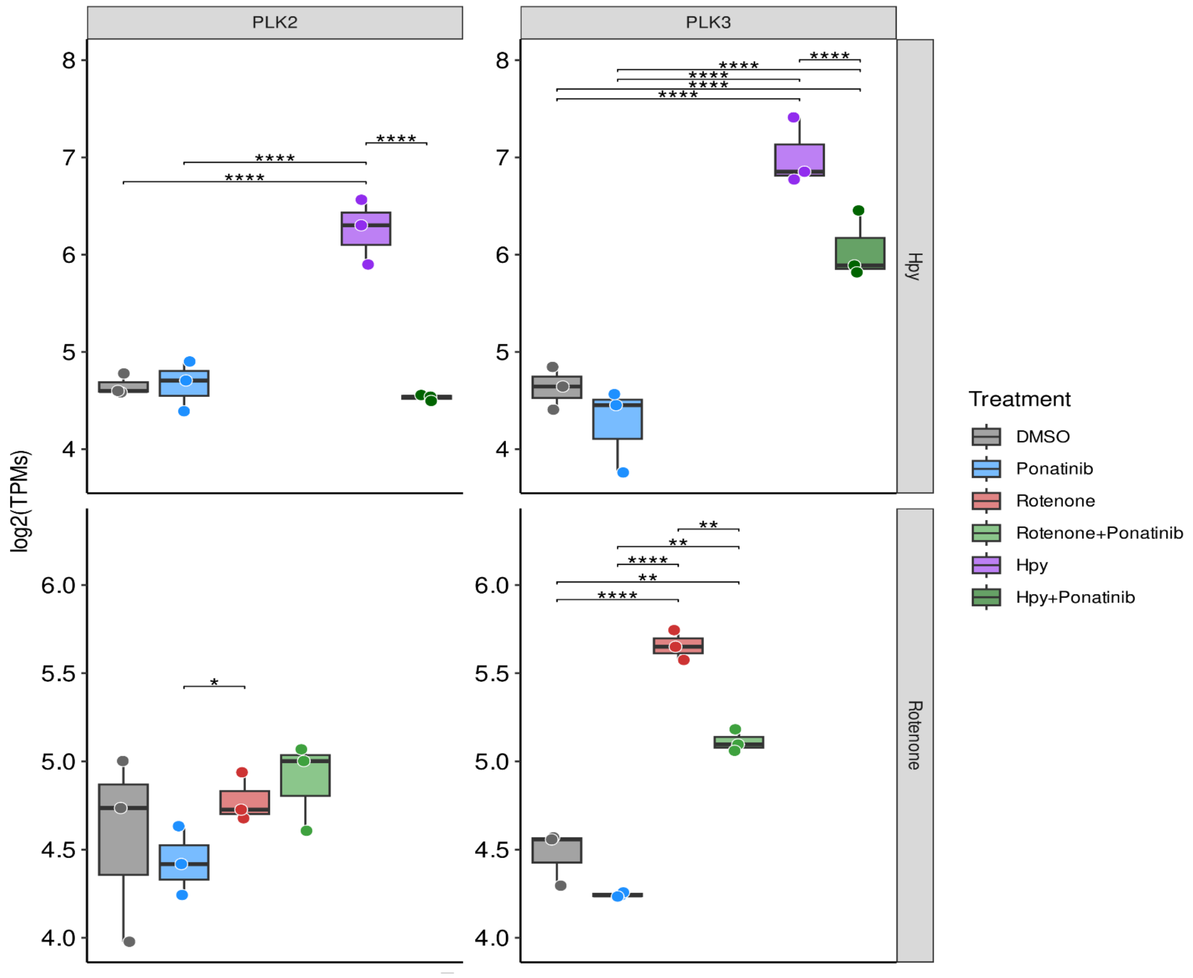

Additional file 5. SH-SY5Y cells were infected with *H. pylori* (Hpy) or treated with rotenone (ROT) and total RNA was extracted after 4h. Differentially expressed genes were identified and RNA counts were shown as log 2 of transcripts per million (TPM). *H. pylori* and rotenone enhanced the gene expression of Polo-like kinases2 (PLK2) and PLK3. Furthermore, *H. pylori*-induced gene expression of PLK2 and PLK3 was rescued by Ponatinib. In contrast, Ponatinib selectively rescued only PLK3 gene expression induced by rotenone. The data represents as n=3 replicates. Means ± SEM are shown. Statistical analysis was performed using Deseq2 R package. ns, not-significant; * $p < 0.05$; ** $p < 0.01$; *** $p < 0.001$.
