# Supplementary material for: Infection and herbicide exposure implicate c-Abl kinase in α-Synuclein Ser129 phosphorylation
Source: Cell Commun Signal. 2025 Sep 23;23:396. doi: 10.1186/s12964-025-02399-2 (PMC12455823; doi:10.1186/s12964-025-02399-2)
Supplement: Supplementary file 3 — Supplementary Material 3: Additional file 3, PDF. (A) Heatmap with Gene Set Enrichment Analysis (GSEA) results using bulk RNAseq data for all 4 conditions. Red colors reflect upregulation of the pathway (GSEA Normalized Enrichment Scores, NES >0). While blue show downregulation (NES < 0) with respect to the control condition indicated at the right side of the “_vs_” Contrast name. Pathways are grouped according to biological function. On the right side, the bar plot represents GSEA FDR adjusted P-values, with vertical red line at FDR=0.20 (-log10 scale). *: FDR < 0.2; ** FDR<0.1, ***: FDR<0.05 [file 12964_2025_2399_MOESM3_ESM.pdf]

Additional file 6. The table represents upstream kinases that show induced activities upon rotenone compared to control treatment. Upstream kinase analysis (UKA) was performed on the raw data obtained from Pamgene Serine-Threonine kinase screening. The preliminary analysis has been performed by UKA algorithm. The row data to draw the kinome tree was further filtered using the threshold cut-off for the median final score (kinase score) > 1.2. The kinase statistic represents the log fold change scaled by the noise. The median kinase statistic<0 is considered as inhibited of activity and median kinase statistic>0 is considered as induced activity.

Kinase Name	Kinase Uniprot ID	Kinase Group	Kinase Family	Max Final score
DAPK3	O43293	CAMK	DAPK	3.673664139
MYLK3	Q32MK0	CAMK	MLCK	3.673664139
DCLK3	Q9C098	CAMK	DCAMKL	2.974694135
AURKA	O14965	CAMK	AUR	2.71942163
RPS6KC1	Q96S38	OTHER	RSKL	2.673664139
RPS6KA3	P51812	AGC-CAMK	RSK	3.673664139
PIM2	Q9P1W9	CAMK	PIM	3.372634143
PKN1	Q16512	AGC	PKN	2.594482893
MAPK7	Q13164	CMGC	MAPK	2.71942163
RPS6KA6	Q9UK32	AGC-CAMK	RSK	3.372634143
COQ8A	Q8NI60	Atypical	ABC1	2.093880542
RPS6KA2	Q15349	AGC-CAMK	RSK	2.527536103
CDKL2	Q92772	CMGC	CDKL	1.910236146
RPS6KA4	O75676	AGC-CAMK	RSK	1.992422902
PIM1	P11309	CAMK	PIM	2.040195683
RPS6KA1	Q15418	AGC-CAMK	RSK	2.060880282
CDK2	P24941	CMGC	CDK	1.910236146
CILK1	Q9UPZ9	CMGC	RCK	1.804432419
ROCK1	Q13464	AGC	DMPK	1.895512889
AURKB	Q96GD4	CAMK	AUR	1.957660795
DYRK1A	Q13627	CMGC	DYRK	1.744245213
RPS6KB1	P23443	AGC	RSK	2.050414849
MAPK12	P53778	CMGC	MAPK	1.974694135
MAPK3	P27361	CMGC	MAPK	2.117361638
CDKL5	O76039	CMGC	CDKL	2.040195683
MAPK11	Q15759	CMGC	MAPK	2.030211463
RPS6KA5	O75582	AGC-CAMK	RSK	2.051195954
CSNK2A1	P68400	CMGC	CK2	1.62834116
CDK5	Q00535	CMGC	CDK	1.563074429
PRKAB1	Q9Y478	NA	NA	1.509311283
PDPK1	O15530	AGC	PKB	1.480539541
NUAK1	O60285	CAMK	CAMKL	1.456180195
MAPK1	P28482	CMGC	MAPK	1.483332441
MAPK9	P45984	CMGC	MAPK	1.477764487
MAPK10	P53779	CMGC	MAPK	1.418391634
MAPK8	P45983	CMGC	MAPK	1.448354857
GSK3A	P49840	CMGC	GSK	1.477764487
MAPK13	O15264	CMGC	MAPK	1.71942163
CDK11A	Q9UQ88	CMGC	NA	1.385862409
CDK9	P50750	CMGC	CDK	1.327311165
PRKY	O43930	AGC	PKA	1.759850287
PRKCZ	Q05513	AGC	PKC	1.816331643
CDK13	Q14004	CMGC	CDK	1.413592751
CDK4	P11802	CMGC	CDK	1.297087182
PRKCH	P24723	AGC	PKC	1.616759288
GSK3B	P49841	CMGC	GSK	1.288057865
MYLK2	Q9H1R3	CAMK	MLCK	1.223356919
CHEK1	O14757	CAMK	CAMKL	1.65247484
CHEK2	O96017	CAMK	RAD53	1.30019695
IKBKE	Q14164	OTHER	IKK	1.556392843

Mean Kinase Statistic	Mean Significance Score	Mean Specificity Score	Mean peptide set size
2.949047483	0.974694135	2.698970004	3
2.995521184	0.974694135	2.598626672	4
2.624403059	0.974694135	1.94367268	3
1.965797985	0.974694135	1.55268161	5
2.415259119	0.974694135	1.614028508	3
1.435955963	0.974694135	1.737602422	21
1.238702052	0.974694135	1.592343954	37
1.787098431	0.974694135	1.435131873	7
1.403928342	0.974694135	1.230517609	13
1.486852078	0.974694135	1.296829781	16
1.271303757	0.883039758	0.779884995	8
1.179874056	0.9124618	0.899264396	23
1.721292922	0.974694135	0.908446264	3
1.50708932	0.974694135	0.790506487	4
1.07620358	0.9124618	0.871911573	60
1.199669427	0.974694135	0.843180389	22
1.229020768	0.974694135	0.827179344	17
1.44230072	0.974694135	0.750335804	3
1.524176419	0.974694135	0.805277119	4
1.495893842	0.974694135	0.783073961	4
1.362841175	0.974694135	0.70965153	3
1.199326659	0.974694135	0.786035083	19
1.218042591	0.974694135	0.707099405	11
1.108310722	0.974694135	0.63600268	20
1.308149609	0.974694135	0.731413132	6
1.191792129	0.974694135	0.65427655	6
1.1078681	0.694648631	0.896456241	44
1.224625474	0.974694135	0.577818379	4
1.136983932	0.974694135	0.553888858	12
1.097392165	0.974694135	0.48769109	4
1.048741592	0.974694135	0.46033466	3
1.055730993	0.974694135	0.457003551	4
1.056725596	0.974694135	0.419995202	18
1.05120283	0.974694135	0.436324133	19
1.013680701	0.974694135	0.376599336	25
1.027245496	0.974694135	0.402489848	26
1.040266061	0.974694135	0.427606934	5
1.037918959	0.974694135	0.437828847	18
0.983495428	0.931107291	0.376659823	4
0.974441283	0.974694135	0.337970916	8
0.9261052	0.788226025	0.344539523	10
1.048266132	0.974694135	0.438413587	9
0.926867494	0.974694135	0.34983534	4
0.91863718	0.943577968	0.291922838	7
1.053521682	0.850229466	0.436394964	13
0.91763082	0.943577968	0.290294466	7
1.064289192	0.694648631	0.528708289	3
1.149896005	0.756880965	0.536929951	9
1.066636324	0.694648631	0.460406396	18
0.987915836	0.850229466	0.338715247	8

Median Final score	Median Kinase Change	Median Kinase Statistic	SD Kinase Statitistic
3.673664139	0.226304279	2.949047483	NA
3.673664139	0.375789851	2.912335109	0.203763436
2.935103512	0.299158222	2.624403059	0
2.594482893	0.325927911	1.965797985	0
2.594482893	0.348389934	2.415259119	0
2.527536103	0.270422884	1.404272006	0.195500943
2.49757288	0.245937412	1.279045885	0.081288801
2.443215218	0.211212879	1.864052792	0.162568733
2.258690791	0.292021707	1.427273142	0.157849637
2.058439678	0.299392405	1.303648659	0.433783417
1.992422902	0.208990028	1.465084958	0.278299447
1.957660795	0.263154738	1.209292637	0.131982872
1.892042289	0.23492923	1.731885582	0.025946613
1.874378307	0.231719882	1.683988617	0.32755385
1.860750782	0.212170872	1.076608856	0.027407606
1.810341279	0.26439416	1.204907341	0.026570064
1.804432419	0.23067854	1.225246746	0.028450958
1.76517912	0.344395778	1.525500259	0.144105828
1.76517912	0.270659498	1.51240199	0.09899656
1.724274132	0.206286107	1.422002688	0.146623212
1.684345665	0.332619022	1.362841175	0
1.660826914	0.235891787	1.18624875	0.09006519
1.65247484	0.285942647	1.199538808	0.092781072
1.640240384	0.250388275	1.126881663	0.098354633
1.638239012	0.289942508	1.319912178	0.068774465
1.633621597	0.279330954	1.199849547	0.225806892
1.601226945	0.246473055	1.136622214	0.045047429
1.546559341	0.29604486	1.219658632	0.014900528
1.524445026	0.246148917	1.136983932	0
1.458820291	0.251159708	1.097392165	0
1.443215218	0.139307436	1.048741592	0
1.433229677	0.142640351	1.055730993	0
1.415985564	0.24887857	1.054587309	0.058708356
1.406492411	0.210267696	1.054902198	0.014546835
1.383629528	0.213735735	1.038604116	0.045051927
1.383629528	0.213735735	1.038604116	0.02683873
1.381408068	0.224071818	1.036109773	0.011755756
1.374811063	0.217983197	1.033474434	0.093741685
1.359796919	0.252775508	1.015977689	0.051158121
1.311936303	0.204913065	0.98442979	0.014982761
1.311936303	0.23106554	0.97586273	0.23254661
1.306308218	0.202185545	0.970278149	0.154752606
1.303530177	0.191862098	0.914113715	0.048312895
1.288057865	0.198890267	0.937280162	0.055928947
1.277464792	0.16134442	1.023420232	0.088949381
1.27572413	0.228153143	0.927225948	0.028785384
1.223356919	0.24974135	1.064289192	NA
1.220432366	0.193439256	1.144560728	0.040899925
1.206097914	0.207181774	1.098412867	0.060631204
1.20090769	0.283931207	0.911833105	0.111303479
