# Supplementary material for: Infection and herbicide exposure implicate c-Abl kinase in α-Synuclein Ser129 phosphorylation
Source: Cell Commun Signal. 2025 Sep 23;23:396. doi: 10.1186/s12964-025-02399-2 (PMC12455823; doi:10.1186/s12964-025-02399-2)
Supplement: Supplementary file 4 — Supplementary Material 4: Additional file 4. (A) SH-SY5Y cells were infected with H. pylori (Hpy) at MOI 100 or pre-treated with 10 μM Asciminib (ASC) and total protein was extracted after 6 h and Western blotting was performed. (B) SH-SY5Y cells were treated either with 10 μM rotenone (ROT) or 10 μM ASC and total protein was extracted after 12 h and Western blotting was performed. (C) SH-SY5Y cells were treated with siRNA (500nM) in combination with Promega HD Fugene reagent for 72 h and protein was extracted and western blotting was performed and the α-syn protein band was quantified (n=2 replicates). (D) K562 (leukemia cell line with constitutive active Bcr-Abl) were treated with 200μM Ponatinib (Pon) in DMEM containing 0% FBS and the total protein protein was extracted in Leammli 2x buffer at different time points (3 h and 6 h) and Western blotting was performed [file 12964_2025_2399_MOESM4_ESM.pdf]

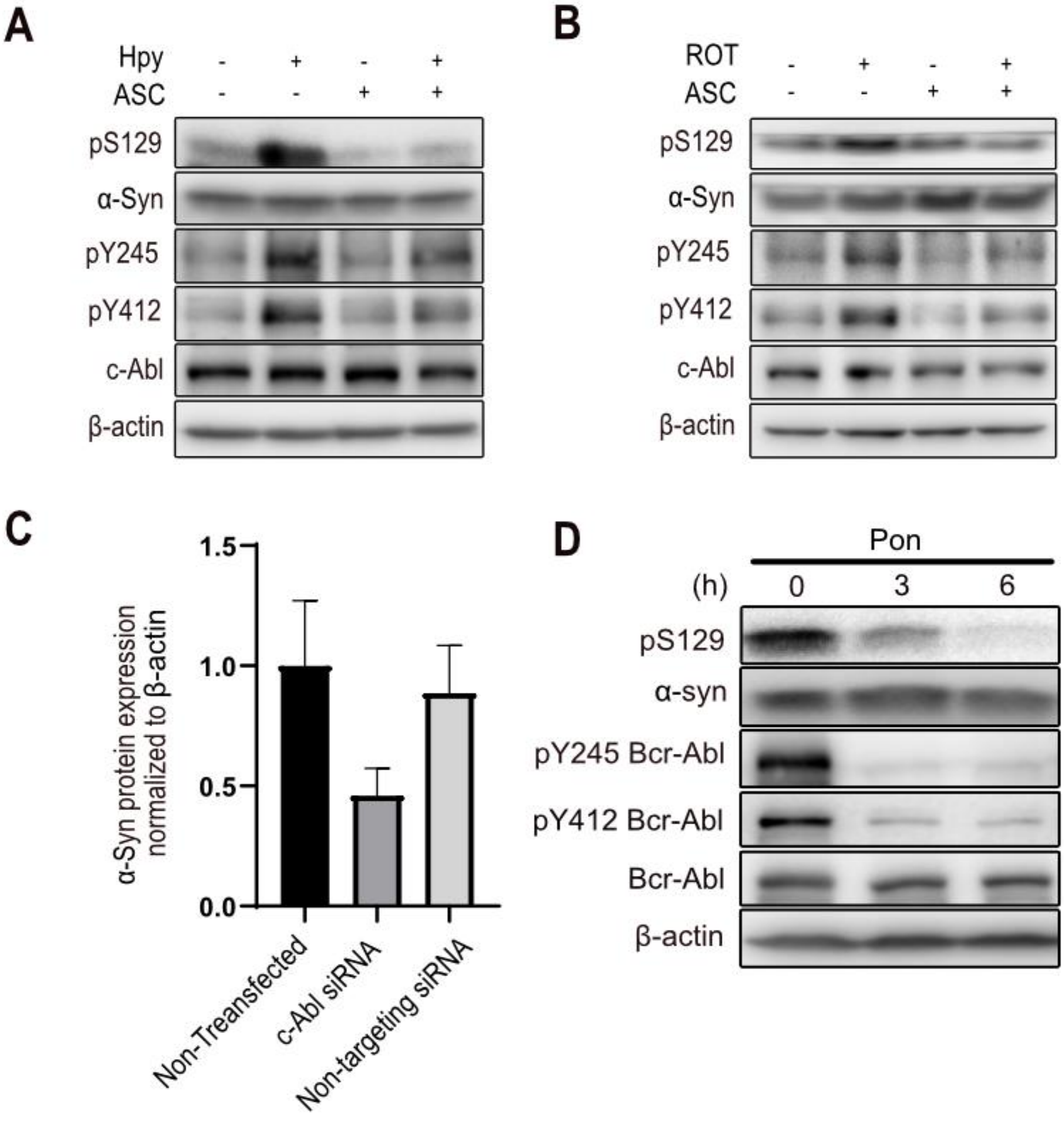

Additional file 4. (A) SH-SY5Y cells were infected with *H. pylori* (Hpy) at MOI 100 or pre-treated with 10 µM Asciminib (ASC) and total protein was extracted after 6h and Western blotting was performed. (B) SH-SY5Y cells were treated either with 10 µM rotenone (ROT) or 10 µM ASC and total protein was extracted after 12 h and Western blotting was performed. (C) SH-SY5Y cells were treated with siRNA (500nM) in combination with Promega HD Fugene reagent for 72 h and protein was extracted and western blotting was performed and the α-syn protein band was quantified (n=2 replicates). (D) K562 (leukemia cell line with constitutive active Bcr-Abl) were treated with 200µM Ponatinib (Pon) in DMEM containing 0% FBS and the total protein protein was extracted in Leammli 2x buffer at different time points (3h and 6h) and Western blotting was performed.
