# Supplementary material for: Infection and herbicide exposure implicate c-Abl kinase in α-Synuclein Ser129 phosphorylation
Source: Cell Commun Signal. 2025 Sep 23;23:396. doi: 10.1186/s12964-025-02399-2 (PMC12455823; doi:10.1186/s12964-025-02399-2)
Supplement: Supplementary file 5 — Supplementary Material 5: Additional file 5. SH-SY5Y cells were infected with H. pylori (Hpy) or treated with rotenone (ROT) and total RNA was extracted after 4h. Differentially expressed genes were identified and RNA counts were shown as log 2 of transcripts per million (TPM). H. pylori and rotenone enhanced the gene expression of Polo-like kinases2 (PLK2) and PLK3. Furthermore, H. pylori-induced gene expression of PLK2 and PLK3 was rescued by Ponatinib. In contrast, Ponatinib selectively rescued only PLK3 gene expression induced by rotenone. The data represents as n=3 replicates. Means ± SEM are shown. Statistical analysis was performed using Deseq2 R package. ns, not-significant; * p < 0.05; ** p < 0.01; *** p < 0.001 [file 12964_2025_2399_MOESM5_ESM.pdf]

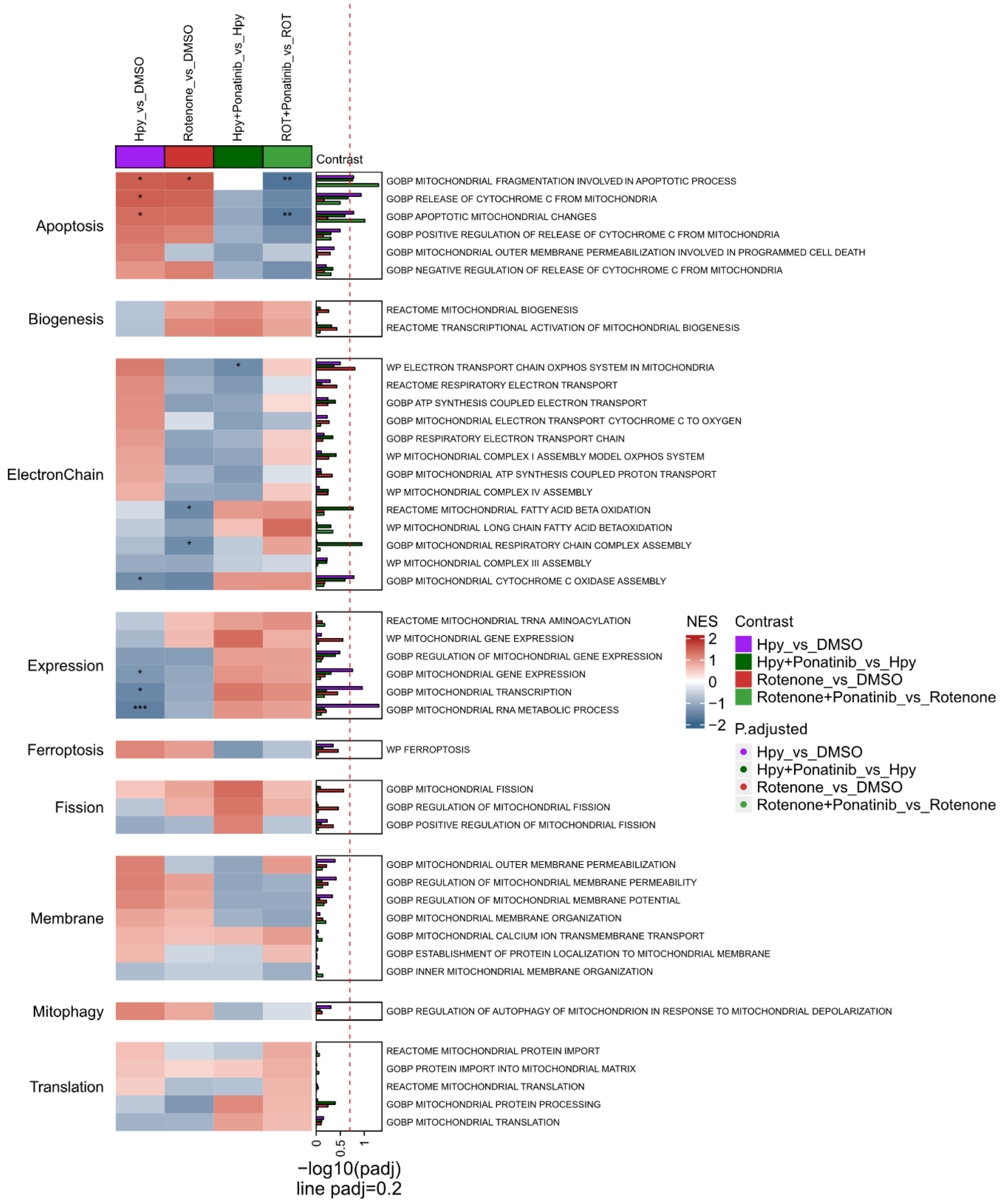

Additional file 3, PDF. (A) Heatmap with Gene Set Enrichment Analysis (GSEA) results using bulk RNAseq data for all 4 conditions. Red colors reflect upregulation of the pathway (GSEA Normalized Enrichment Scores, NES > 0). While blue show downregulation (NES < 0) with respect to the control condition indicated at the right side of the "_vs_" Contrast name. Pathways are grouped according to biological function. On the right side, the bar plot represents

GSEA FDR adjusted P-values, with vertical red line at FDR=0.20 (-log10 scale). *: FDR < 0.2; ** FDR<0.1, ***: FDR<0.05
