# Supplementary material for: Infection and herbicide exposure implicate c-Abl kinase in α-Synuclein Ser129 phosphorylation
Source: Cell Commun Signal. 2025 Sep 23;23:396. doi: 10.1186/s12964-025-02399-2 (PMC12455823; doi:10.1186/s12964-025-02399-2)
Supplement: Supplementary file 6 — Supplementary Material 6: Additional file 6. The table represents upstream kinases that show induced activities upon rotenone compared to control treatment. Upstream kinase analysis (UKA) was performed on the raw data obtained from Pamgene Serine-Threonine kinase screening. The preliminary analysis has been performed by UKA algorithm. The row data to draw the kinome tree was further filtered using the threshold cut-off for the median final score (kinase score) >1.2. The kinase statistic represents the log fold change scaled by the noise. The median kinase statistic<0 is considered as inhibited of activity and median kinase statistic>0 is considered as induced activity [file 12964_2025_2399_MOESM6_ESM.pdf]

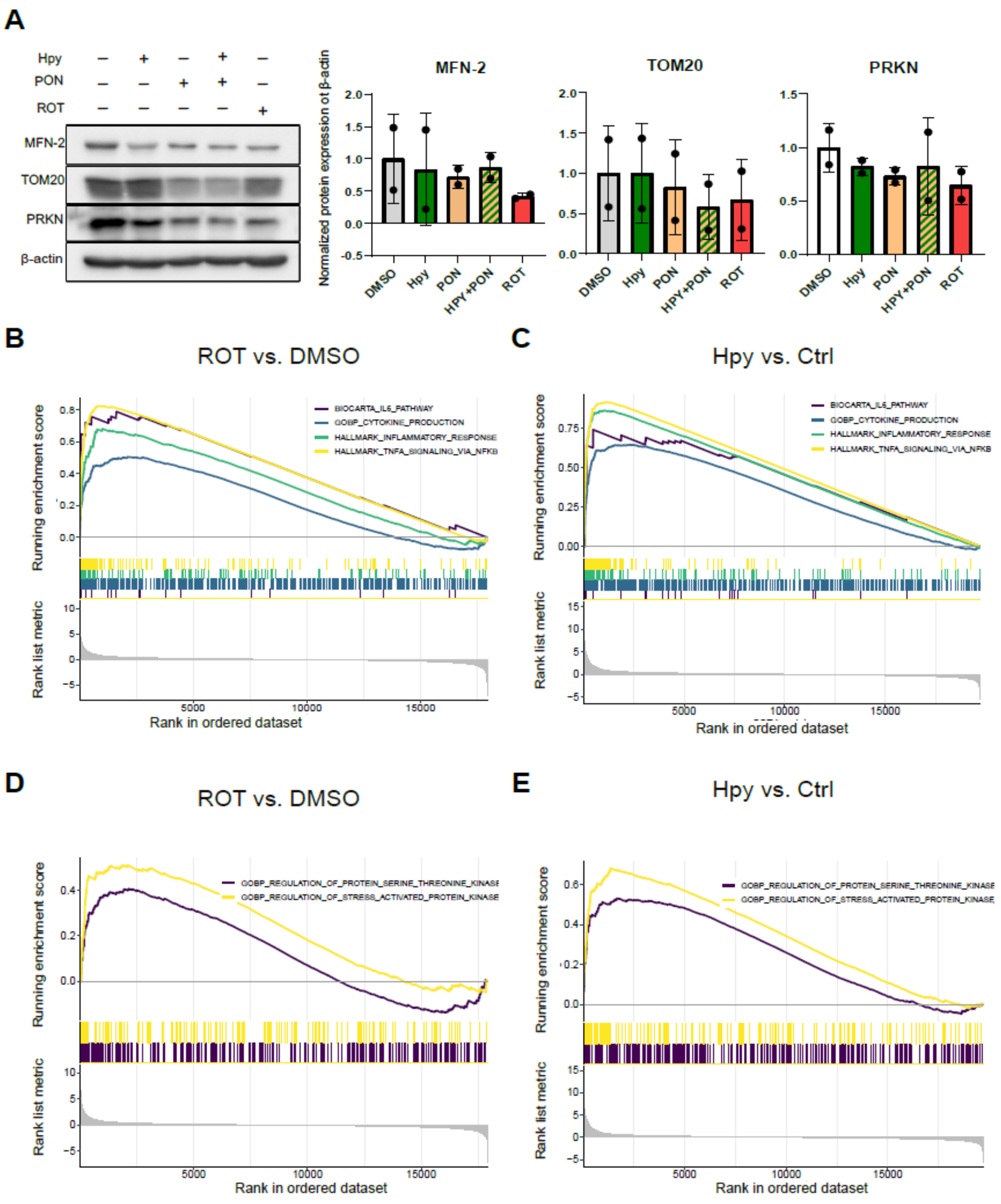

F

ID	Wikipathway
WP254	Apoptosis
WP4754	Il8
WP5083	Neuroinflammation and glutamatergic signaling
WP5504	Disorders of Mitochondrial homeostasis, dynamice, protein import and quality control
WP2884	Nrf2

Additional file 2. (A) SH-SY5Y cells were treated with rotenone (ROT) or infected with *H. pylori* (Hpy) at moi100 for 6h and Western blotting was performed on the extracted total protein. Some markers for mitochondrial dysfunction such as mitofusin-2 (MFN-2), TOM20

and parkin (PARKN) were evaluated and the intensity of the protein bands were quantified (n=2 replicate). (B-F) SH-SY5Y cells were treated with rotenone (ROT) or (C) infected with *H. pylori* (Hpy) and total RNA was extracted after 4h. Differentially expressed genes were identified using the DESeq2 R package (v 1.40.2). Gene Set Enrichment Analysis (GSEA) was performed on the gene list ranked by log2 Fold Changes using the *fgsea* (v 1.26.0) implementation of the algorithm implemented in the clusterProfiler (v 4.8.3) R package. The displayed pathways were filtered based on significance, with an adjusted p-value threshold of <0.05. (B) and (C) represent enrichment of inflammatory pathways. (D) and (E) show activation of cell kinases including tyrosine and serine-threonine kinases. The data represents as n=3 replicates. (F) The identifier of each regulated Wikipathway is represented in the table.
