# Supplementary material for: Infection and herbicide exposure implicate c-Abl kinase in α-Synuclein Ser129 phosphorylation
Source: Cell Commun Signal. 2025 Sep 23;23:396. doi: 10.1186/s12964-025-02399-2 (PMC12455823; doi:10.1186/s12964-025-02399-2)
Supplement: Supplementary file 7 — Supplementary Material 7: Additional file 7. The table represents upstream kinases that show induced activities upon rotenone + ponatinib compared to rotenone treatment. Upstream kinase analysis (UKA) was performed on the raw data obtained from Pamgene Serine-Threonine kinase screening. The preliminary analysis has been performed by UKA algorithm. The row data to draw the kinome tree was further filtered using the threshold cut-off for the median final score (kinase score) >1.2. The kinase statistic represents the log fold change scaled by the noise. The median kinase statistic<0 is considered as inhibited of activity and median kinase statistic>0 is considered as induced activity [file 12964_2025_2399_MOESM7_ESM.pdf]

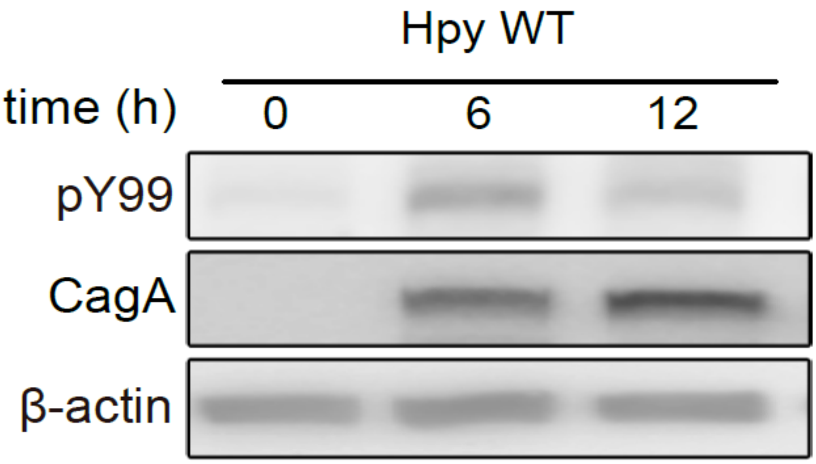

Additional file 1. SH-SY5Y cells were infected with *H. pylori* wild type at MOI 100. Total protein was extracted at different time points and Western blotting was performed to check phosphorylation of CagA at Y99. β-actin is used as loading control.
